# ﻿The genus *Coleus* (Lamiaceae) in Central Africa (Democratic Republic of the Congo, Rwanda, Burundi), with the description of 15 new species

**DOI:** 10.3897/phytokeys.246.129476

**Published:** 2024-09-02

**Authors:** Pierre J. Meerts, Alan J. Paton

**Affiliations:** 1 Meise Botanic Garden, Nieuwelaan 38, BE-1860 Meise, Belgium Meise Botanic Garden Meise Belgium; 2 Université Libre de Bruxelles, Av. F.D. Roosevelt 50 CP244, BE-1050 Brussels, Belgium Université Libre de Bruxelles Brussels Belgium; 3 Fédération Wallonie-Bruxelles, Service général de l’Enseignement supérieur et de la Recherche scientifique, Brussels, Belgium Fédération Wallonie-Bruxelles, Service général de l’Enseignement supérieur et de la Recherche scientifique Brussels Belgium; 4 The Herbarium, Royal Botanic Gardens, Kew, Richmond TW9 3AB, UK The Herbarium, Royal Botanic Gardens Richmond United Kingdom

**Keywords:** Distribution, endemic, flora, identification key, Katanga, new records, taxonomy

## Abstract

The genus *Coleus* is revised for DR. Congo, Rwanda, Burundi, based on herbarium taxonomy. Ninety-five taxa are reported (89 species, 1 subspecies, 5 varieties). Fifteen new species and one new variety are described (*Coleusduvigneaudii*, C.esculentusvar.kolweziensis, *C.hildei*, *C.kaminaensis*, *C.kundelunguensis*, *C.linarioides*, *C.lisowskii*, *C.marunguensis*, *C.minusculus*, *C.mitwabaensis*, *C.mystax*, *C.pengbelensis, C.piscatorum*, *C.pseudoschizophyllus*, *C.ruziziensis* and *C.zigzag*). Fourteen species are newly recorded in DR. Congo and two species are newly recorded in Burundi. Four new combinations are made (Coleusbetonicifoliusvar.kasomenensis, C.esculentusvar.densus, C.esculentusvar.primulinus and *C.parvifolius*). Ten names are lectotypified. One name is neotypified. Thirteen new synonyms are reported. Particular attention is paid to the *Coleusbojeri* complex. Three names are resurrected to accommodate the extensive variation patterns in Central Africa (*C.chevalieri*, *C.collinus* and *C.heterotrichus*); their distribution in Africa is outlined and the circumscription of *C.bojeri* is amended accordingly. Fifteen taxa are endemic to the study region. A determination key is provided.

## ﻿﻿Introduction

The circumscription of the genus *Coleus* Lour. (Lamiaceae) has changed considerably in the last decades, producing a copious amount of synonymy. *Coleus* was transferred to *Plectranthus* L’Hér. by Morton (1962). That concept was followed by Flora of West Tropical Africa (Morton 1963), the Flore du Rwanda (Troupin and Ayobangira 1985) and Lebrun and Stork (1997). Harley et al. (2004) further merged *Solenostemon* Thonn., *Englerastrum* Briq. and *Holostylon* Robyns & Lebrun in *Plectranthus*, whereas *Pycnostachys* Hook. was kept distinct; this concept was followed by Flora of Tropical East Africa (Paton et al. 2009) and Flora Zambesiaca (Paton et al. 2013). However, recent phylogenetic analyses, based on molecular markers, have shown that this expanded *Plectranthu*s is polyphyletic and that *Coleus* and *Plectranthus* should be kept distinct (Paton et al. 2018). The genus *Coleus* thus resuscitated now comprises 294 species, i.e. all the species formerly included in *Pycnostachys*, *Solenostemon*, *Holostylon* and *Englerastrum*, in addition to part of *Plectranthus* sensu Morton (1962) (Paton et al. 2019). Part of the remaining species of *Plectranthus* are now accommodated in a new genus, i.e. *Equilabium* Mwany., A.J.Paton & Culham (Paton et al. 2018). The necessary nomenclatural combinations have been published by Paton et al. (2019). This concept is also followed in the Flore du Gabon (Paton 2022).

For Central Africa (defined here as DR. Congo, Rwanda, Burundi), only local accounts of *Coleus* are available, including Troupin and Ayobangira (1985) for the Flore du Rwanda and Robyns (1947) for the Virunga National Park. Other important references for DR. Congo include De Wildeman (1920), Robyns and Lebrun (1929), Bruce (1940) and Robyns (1943). In order to prepare the treatment of *Coleus* for the Flore d’Afrique Centrale, we have critically revised all the relevant materials from the Democratic Republic of the Congo (DR. Congo), Rwanda and Burundi. During the preparation of this revision, particular attention was paid to the complex of *C.bojeri*, a particularly difficult group which was left incompletely resolved by Paton et al. (2009, 2013). We here present a key to the species, a check-list with a revised synonymy and original descriptions for the species not included in recent floras.

## ﻿﻿Materials and methods

Herbarium specimens of *Coleus* collected in DR. Congo, Rwanda and Burundi were studied in BR which hosts the largest collection for that region (available online [https://www.botanicalcollections.be]), BRLU, K and POZG (available online [https://amunatcoll.pl/]). Additional specimens, in particular from B, BM, COI, HBG, K, LISC, LWI, P, WAG and YBI were investigated using the JSTOR Global Plants facility (https://plants.jstor.org/) and GBIF (https://www.gbif.org) and online databases of the respective herbaria. All cited specimens have been seen (except otherwise indicated).

Plants of the World Online (POWO, http://www.plantsoftheworldonline.org/, consulted 25 June 2023) was used to build up a first checklist. Fifty-seven species of *Coleus* were hitherto accepted for DR. Congo, Rwanda and Burundi.

For the *Coleusbojeri* complex, all the names based on type materials from DR. Congo, Rwanda and Burundi (i.e. *Coleusclaessensii* De Wild., *C.collinus* Robyns & Lebrun, *C.dewevrei* Briq., *C.heterotrichus* Briq., *C.homblei* De Wild., *C.ringoetii* De Wild., *C.platostomoides* Robyns & Lebrun, *C.quarrei* De Wild. and *C.termetophilus* De Wild.), were synonymised by Paton et al. (2009, 2013) as *Plectranthusbojeri* and by Paton et al. (2019) as *Coleusbojeri*. *Coleusdelpierrei* De Wild., synonymised with *C.welwitschii* by Paton et al. (2019) was also included, on account of its close resemblance with *C.bojeri*. The original materials of all these names have been critically revised.

## ﻿﻿Taxonomic treatment

Our revision shows that 95 taxa of *Coleus* (89 species, 1 subspecies, 5 varieties) occur in Central Africa; all taxa occur in DR. Congo, 34 species and 29 species occur in Burundi and Rwanda, respectively. Fifteen species and one variety new to science are described (*Coleusduvigneaudii*, C.esculentusvar.kolweziensis, *C.hildei*, *C.kaminaensis*, *C.kundelunguensis*, *C.linarioides*, *C.lisowskii*, *C.marunguensis*, *C.minusculus*, *C.mitwabaensis*, *C.mystax*, *C.piscatorum*, *C.pengbelensis*, *C.pseudoschizophyllus*, *C.ruziziensis* and *C.zigzag*). Four new combination are made (Coleusbetonicifoliusvar.kasomenensis, C.esculentusvar.densus, C.esculentusvar.primulinus and *C.parvifolius*). Thirteen new synonyms are reported.

In addition to the new species, 14 species are newly recorded in DR. Congo and two species are newly recorded in Burundi. A total of fifteen taxa are endemic to the study area, i.e. all new taxa, except *C.pengbelensis* (also present in Central African Republic) and *C.zigzag* (also present in Uganda), in addition to the previously described *C.globosus*.

Ten names are lectotypified (*C.celsus* A.J.Paton, *C.claessensii* De Wild., *C.delpierrei* De Wild., *C.homblei* De Wild., *C.kasomenensis* De Wild., *C.kisanfuensis* De Wild., *C.seretii* De Wild., *C.termetophilus* De Wild., *Plectranthushockii* De Wild. and *Pycnostachyscongensis* Gürke). One name is neotypified (*Pycnostachysgoetzenii* Gürke).

### ﻿﻿The *Coleusbojeri* complex

Paton et al. (2009, 2013) adopted a very broad circumscription of *Coleusbojeri*, comprising virtually all the annual taxa formerly referred to the genus *Solenostemon* while admitting that variation in DR. Congo was more complex compared to neighbouring regions and in need of revision. Nine names, based on type materials from DR. Congo, were synonymised with *C.bojeri* by Paton et al. (2009, 2013) (*C.claessensii* De Wild., *C.collinus* Lebrun & L. Touss., *C.dewevrei* Briq., *C.heterotrichus* Briq., *C.homblei* De Wild., *C.platostomoides* Robyns & Lebrun, *C.quarrei* De Wild., *C.ringoetii* De Wild. and *C.termetophilus* De Wild.).

Our revision shows that flower morphology is relatively uniform, but with little information value in this group. However, traits of taxonomic value include bract persistence, pubescence pattern of the stem, especially in the inflorescence, verticil spacing, cincinni length and leaf blade size and shape (Table 1). Variation in DR. Congo can be accommodated in four different species. Three binomials are resurrected i.e. *C.chevalieri* Briq., *C.collinus* Lebrun & L.Touss. and *C.heterotrichus* Briq. Accordingly, the circumscription of the binomial *Coleusbojeri* is amended. It is restricted here to plants with early deciduous bracts, stem indumentum (especially in the inflorescence) homotrichous, consisting only of retrorse, more or less appressed hairs, without glandular hairs and long patent hairs and cincinni rachis not exceeding 7 mm long in fruit. See Table 1 for a comparison of the four species. Finally, *C.homblei* De Wild. is resurrected as an earlier synonym of *C.sigmoideus* A.J.Paton.

**Table 1.** Comparison of four taxa in the *Coleusbojeri* species complex. Cincinni length refers to the length of the rachis of the cyme, excluding pedicels and flowers.

	*C.bojeri*	*C.chevalieri*	*C.collinus*	*C.heterotrichus*
Bracts	caducous	persistent	persistent	caducous
Indumentum of stem	Short, retrorse, appressed eglandular hairs	Short, retrorse, appressed eglandular hairs	Long, retrorse and patent eglandular hairs	Very short, often papilliform, patent eglandular hairs, microglandular hairs, and sparse long hairs
Cincinni length at fruiting	1–7(–12) mm	2–3(–6) mm	0–4 mm	5–50 mm
Leaf blade size	1.5–4.5(–7.5) × 1.0–4.0(–6.2) cm	3–10(–11) × 1–5.5 cm	0.9–2.2 × 0.7–2.0 cm	1.0–7.0(–8.5) × 1.0–5.0(–8.5) cm
Verticil spacing	(5–)10–25(–30) mm	5–10 mm	7–15 mm	10–25(–40) mm

### ﻿﻿Key to the species of *Coleus* in DR. Congo, Rwanda, Burundi

**Notes**: 1. *C.tetragonus*, found in Zambia very close to the border of DR. Congo, is included in the key; 2. All measurements of calyx refer to the fruiting state unless otherwise stated.

#### ﻿﻿Key to the groups

**Table:** 

1	Corolla yellow or orange	**Group 1**
–	Corolla white, pink, mauve, purple or blue	**2**
2	Flowers sessile on inflorescence axis, forming a compact spike-like inflorescence; calyx lobes all similar in size and shape, subulate, becoming spinescent after anthesis	**Group 2**
–	Flowers mostly pedicellate; inflorescence mostly lax, more rarely congested; calyx lobes more or less unequal, never all subulate, not spinescent	**3**
3	Inflorescence cylindrical, congested, with axis not visible between the cymes; pedicel 0–3 mm long	**Group 3**
–	Inflorescence lax, with the axis conspicuous between most of the cymes; pedicel (1–)2–12 mm long	**4**
4	Flower solitary in the axil of a bract; 1 or 2 flowers to each inflorescence node	**Group 4**
–	Flowers 2–20 in the axil of each bract	**5**
5	Median lobes of lower lip of calyx fused over most of their length into a bidentate lip, much exceeding the lateral lobes	**Group 5**
–	Median lobes of lower lip of calyx free, not fused into a lip	**Group 6**

#### ﻿﻿Group 1. Corolla yellow or orange

**Table:** 

1	Flowers solitary or in pairs at each inflorescence node	**2**
–	Flowers > 2 at each inflorescence node	**5**
2	Stem with stiff lignified bristles	**3**
–	Stem without stiff lignified bristles	**4**
3	Annual with unbranched shoots < 0.5 m; fruiting calyx 6–10 mm long	**(*C.tetragonus*)**
–	Shrub with branched perennial shoots, up to 2 m; fruiting calyx ca. 4.5 mm long	***C.conglomeratus***
4	Shoot slender, < 40 cm high; leaves (1.0–)1.5–2.7 × (0.1–)0.3–1.0 cm; inflorescence terminal; rootstock a globose tuber ca. 1 cm	***C.mitwabaensis***
–	Shoot robust, 60–200 cm high; leaves 3.0–8.0(–20) × 1.0–3.0(–8) cm; inflorescence of lateral thyrses; rootstock a rhizome sometimes with elongated tubers	***C.esculentus***
5	Geofrutex of burnt savannah, leafless at flowering, < 25 cm high; corolla pale yellow with purple spots	***C.buchananii***
–	Shrub of moist forest, leafy at flowering, > 25 cm high; corolla deep orange yellow	**6**
6	Stem with fusiform propagules in the upper axils; hooked hairs present on propagules and, sometimes, near shoot apices; leaf blade 5–14 × 2–5 cm, not decurrent into the petiole; pedicel 4–10 mm long	***C.melleri***
–	Stem without propagules; hooked hairs lacking; leaf blade 15–30 × 6–10 cm, long attenuate into the narrowly winged petiole; pedicel ca. 4 mm long	***C.decurrens***

#### ﻿﻿Group 2. Inflorescence compact, spike-like; flowers sessile; calyx lobes subulate, spinescent

**Table:** 

1	Leaves sessile or petiole < 0.5 cm, blade occasionally attenuate into a pseudopetiole	**2**
–	Leaves conspicuously contracted into a distinct petiole > (0.5–)1.0 cm	**14**
2	Corolla 4–7 mm long	**3**
–	Corolla ≥ 8 mm long	**6**
3	Floral bracts densely ciliate, with cilia length exceeding bract width	***C.deflexifolius***
–	Floral bracts with cilia shorter than bract width or only sparsely ciliate or not ciliate	**4**
4	Dorsal side of calyx tube curving over to form a hood over the mouth; calyx lobes slightly curving upwards	***C.ruandensis***
–	Calyx tube not as above; calyx lobes straight	5
5	Leaves opposite, without fascicles of young leaves in the axils; inflorescence 5–7 mm broad at anthesis (corolla excluded), 8–12 mm broad in fruit; calyx tube funnel-shaped	***C.stenostachys***
–	Leaves with fascicles of small leaves in the axils; inflorescence > 7 mm broad at anthesis (corolla excluded), 14–23 mm broad in fruit; calyx tube ventrally gibbose	***C.dewildemanianus***
6	Fascicles of small leaves or short branches in the axils of leaves	**7**
–	No fascicles of leaves or short branches in the axils of leaves	**8**
7	Inflorescence 20–45 mm long in fruit; leaf blade 0.5–1.5(–3.0) cm long	***C.parvifolius***
–	Inflorescence 55–140 mm long in fruit; leaf blade 1.5–6.0 cm long	***C.descampsii***
8	Secondary veins diverging at a very open angle (60–90°), fusing into a submarginal vein; mid-vein thickened	***C.stuhlmannii***
–	Secondary veins diverging at a narrow angle (<60°), not joining into a marginal vein; mid-vein not thickened	**9**
9	Leaves heteromorphic, the median and upper ones with blade almost linear, 0.2–0.6 cm wide, the lowermost ones abruptly broader, ovate-elliptic, obovate-elliptic, to broadly elliptic, (0.5–)1.0–1.5 cm wide	***C.lisowskii***
–	Leaves homomorphic, the lowermost ones not abruptly broader	**10**
10	Leaves amplexicaulous, strongly recurving, much longer than the internodes; calyx throat and base of lobes tomentose	***C.pseudospeciosus***
–	Leaves attenuate at base, ascending to spreading, not exceeding internode length by much; calyx throat glabrous to pubescent	**11**
11	Secondary veins almost parallel to mid-vein, prominent on both surfaces; leaf blade narrowly obovate; bracts forming a conspicuous coma; calyx compressed dorsally	***C.prittwitzii***
–	Secondary veins divergent, not prominent; leaf blade narrowly elliptic to linear; bracts not forming a conspicuous coma; calyx not compressed dorsally	**12**
12	Inflorescence 75–115 mm long in fruit; calyx tube ventrally compressed at throat, then gibbous, the dorsal side curving outwards and almost forming a hood over the throat; calyx lobes (7–)10–13 mm long at fruiting, with long eglandular hairs near base	***C.affinis***
–	Inflorescence 30–55 mm long in fruit; calyx tube not compressed at throat; calyx lobes 3–7(–10) mm, with short glandular hairs	**13**
13	Leaves 0.4–2.0 cm broad, > 7× as long as broad, somewhat pubescent beneath; calyx tube somewhat ventrally gibbous, with short glandular hairs; calyx lobes not winged; bracts ca. 3 mm long	***C.scruposus***
–	Leaves 1.4–4.0 cm broad, < 7× as long as broad, densely pubescent beneath; calyx tube funnel-shaped, with eglandular hairs; calyx lobes narrowly winged; bracts 4–8 mm long	***C.sphaerocephalus***
14	Dorsal side of calyx tube curving outwardly to form a hood over calyx mouth; corolla 4–5 mm long; calyx lobes ± 2.5 mm, somewhat upwardly curving	***C.ruandensis***
–	Dorsal side of calyx straight, not forming a hood over the mouth; corolla 6–19 mm long; calyx lobes 3–9 mm, straight	**15**
15	Secondary veins diverging at a very open angle (60–90°), fusing into a marginal vein; leaf blade 0.4–2.2 cm broad, at least 5× as long as broad	***C.stuhlmannii***
–	Secondary veins diverging at a narrower angle (< 60°), not fusing into a submarginal vein; leaf blade 1.5–7.0 cm broad, 2–3× as long as broad	**16**
16	Leaf blade broadly ovate, length/width ratio < 2, almost truncate at base; marginal lobes < 8 on either side	***C.batesii***
–	Leaf blade ovate to elliptic, length/width ratio > 2, base cuneate to attenuate; marginal lobes > 10 on either side	**17**
17	Leaf undersurface mostly with stellate hairs; corolla 15–20 mm long; stamens exserted > 5 mm; calyx lobes 8–13 mm long	***C.elliotii***
–	Leaf undersurface with simple hairs; corolla 6–15(–20) mm long; stamens included in the corolla or exserted over 1–4 mm; calyx lobes 2.5–9 mm long	**18**
18	Calyx lobes 2.5–3.5(–4) mm long	**19**
–	Calyx lobes 4–9 mm long	**20**
19	Calyx throat and base of lobes tomentose; bract cilia somewhat undulate	***C.eminii***
–	Calyx throat and base of lobes not tomentose; calyx lobes very shortly pubescent; bract cilia straight	***C.erici-rosenii***
20	Corolla 6–8(–9) mm long, generally pale-coloured	***C.meyeri***
–	Corolla 12–15(–20) mm long, generally with vivid colours	**21**
21	Calyx lobes narrowly winged; petiole 0–1 cm; leaf blade oblong-elliptic, widest near the middle; leaf blade coriaceous	***C.sphaerocephalus***
–	Calyx lobes not winged; petiole 1–3.5 cm; leaf blade ovate, widest under the middle; leaf blade membranous	**22**
22	Inflorescence 45–80 mm long, distinctly tapering and less dense apically; calyx tube with sessile glands rare; calyx lobes 6–9 mm long, eglandular	***C.goetzenii***
–	Inflorescence 25–45 mm long, not tapering and remaining dense near the apex; calyx tube with many red sessile glands; calyx lobes 4.5–6 mm long, thinly glandular	***C.schliebenii***

#### ﻿﻿Group 3. Inflorescence dense, spiciform, cylindrical; corolla violet, purplish or blue

**Table:** 

1	Calyx hairy inside throat; verticils 6-flowered; annual	**C.caninussubsp.flavovirens**
–	Calyx glabrous inside throat; verticils 6–40-flowered; perennial	**2**
2	Corolla 3–9 mm long; calyx 2–3.5 mm long	**3**
–	Corolla 8–22 mm long; calyx 5–9 mm long	**5**
3	Inflorescences on short lateral shoots; pedicel 0–1 mm long; verticils 6–10-flowered; stamens with filaments fused; leaf blade 1–2 cm long	***C.guerkei***
–	Inflorescence terminal; pedicel 1–3 mm long; verticils 10–40-flowered; stamens free; leaf blade 1.2–6.0 cm long	**4**
4	Leaf petiolate; corolla 5–9 mm long, with tube 3–4.5 mm long	***C.succulentus***
–	Leaf sessile; corolla 3–5 mm long, with tube 2–2.5 mm long	***C.cylindraceus***
5	Calyx upright; calyx lobes arranged in two groups, 3 upper lobes and 2 lower lobes; plant almost leafless at flowering	***C.lactiflorus***
–	Calyx patent; calyx lobes in a 1+4 pattern; plant leafy at flowering	**6**
6	Upper lobe of calyx forming a hood over the throat, the other four lobes much smaller	***C.engleri***
–	Upper lobe of calyx not forming a hood over the throat, the other four lobes less different	**7**
7	Calyx with pale sessile glands; inflorescence terminal, often subtended by subsessile lateral inflorescences, greenish, villose, with bracts much exceeding flowers; verticils 12–20-flowered	***C.stachyoides***
–	Calyx with red sessile glands; inflorescence terminal not subtended by lateral inflorescences, purplish, not villose, with bracts inconspicuous; verticils 6–12-flowered	***C.betonicifolius***

#### ﻿﻿Group 4. Inflorescence lax with the axis conspicuous between most of the single flowered cymes; flowers solitary; corolla violet, purplish or blue

**Table:** 

1	Petiole 2–7 cm; leaf blade 4–10 cm broad; calyx bearded inside throat; plant leafy at flowering, in evergreen forest	***C.longipetiolatus***
–	Petiole shorter, leaf blade narrower; calyx throat glabrous inside; plant with or without leaves at flowering, in dry woodland and savannah	**2**
2	Leaves linear, 1.5–3.5 × 0.1–0.4 cm, margin strongly revolute, upper surface glabrous; internodes ca. 1 cm	***C.linarioides***
–	Leaves different or absent at flowering; margin not strongly revolute, upper surface usually with hairs; internodes much longer	**3**
3	Annual, with weak roots; calyx not caducous at fruiting	**4**
–	Perennial, with thickened rootstock; calyx caducous or persistent	**8**
4	Pedicel 6–20 mm long, much exceeding calyx	***C.gracillimus***
–	Pedicel 1–6 mm long, shorter or equalling calyx	**5**
5	Upper calyx lobe ovate, very different in shape from the other four lobes, these narrowly triangular, acute to acuminate; calyx 5–10 mm long in fruit	***C.efoliatus***
–	Upper calyx lobe triangular, similar in shape to the others; calyx 2.5–4 mm long in fruit	**6**
6	Pedicel and calyx with patent 2–5 mm long smooth cilia	***C.mystax***
–	Pedicel shortly pubescent or glabrous	**7**
7	Plant leafless at flowering; stem with 3 to 5 pairs of opposite branches, diverging at a very open angle, almost horizontal, each with 1 or 2 dichotomous ramifications; corolla ca. 10 mm long; inflorescence 3–7 mm long	***C.piscatorum***
–	Plant leafy at flowering; branching pattern different; corolla 4–5 mm long; inflorescence 3–12 cm long	***C.rhodesianus***
8	Dwarf plant < 10 cm, leafless at flowering, glutinous; rootstock a globose to elliptic tuber	***C.minusculus***
–	Plant > 10 cm high, leafy or not at flowering, not glutinous; rootstock a rhizome	**9**
9	Pedicel jointed near the middle	**10**
–	Pedicel jointed near tip or without a joint	**11**
10	Plant robust, 100–150 cm high; leaves petiolate; leaf blade broadly ovate; thyrses ca. 1.5 cm long, with flowers solitary, arranged helicoidally	***C.celsus***
–	Plant more slender, 30–50 cm high; leaves sessile; leaf blade narrowly ovate-elliptical; thyrses 4–20 cm long, with flowers mostly opposite	***C.kundelunguensis***
11	Pedicel 5–25 mm long, filiform; rachis slightly zigzagging near apex; inflorescence a large diffuse panicle	**12**
–	Pedicel 3–14 mm long, not filiform; rachis straight near apex; inflorescence different	**13**
12	Leaves subsessile; leaf blade elliptic to ovate-elliptic, at least 4× as long as broad; anthers not forming a pouch; style not divided or shallowly lobed; calyx lobes subulate	***C.kaminaensis***
–	Leaves petiolate; leaf blade broadly ovate, ca. 2 × as long as broad; anther forming a pouch, opening near apex; style with two branches; calyx lobes deltate	***C.gracilipedicellatus***
13	Main shoot ending in a sterile leafy twig; inflorescence lateral, often exceeding main shoot; calyx in fruit 5–7 mm long; pedicel 4–6 mm long, attached almost centrally on calyx; leaf blade rounded at apex	***C.modestus***
–	Main shoot ending in flowering axis; inflorescence terminal; calyx in fruit 9–12 mm long; pedicel 3–14 mm long, attached asymmetrically in front of upper calyx lobe; leaf blade acute to rounded at apex	**14**
14	Plant leafless at flowering; pedicel jointed, with the distal part more pubescent than the proximal part, with eglandular hairs	***C.articulatus***
–	Plant leafy at flowering; pedicel without a joint, uniformly pubescent, with glandular and eglandular hairs	**15**
15	Leaves 3-whorled; mid-leaves with blade narrowly elliptic, oblong-elliptic to almost linear, 6–9 × 0.5–1.3 cm, cuneate at base, acute at apex	***C.duvigneaudii***
–	Leaves almost always opposite; mid-leaves with blade ovate-elliptic, 2–7 × 0.5–2 cm, base rounded to almost clasping stem, mostly obtuse at apex	***C.foliatus***

#### ﻿﻿Group 5. Calyx with two lower lobes fused

**Table:** 

1	Leaf blade pinnatifid to deeply toothed	***C.pseudoschizophyllus***
–	Leaf blade crenate or serrate	**2**
2	Plant prostrate, rooting at nodes, forming carpets; leaf blade almost round, 1–2.5 × 1–2.5 cm; inflorescence of one or two verticils	***C.repens***
–	Plant ascending to erect, not forming carpets; leaf blade ovate, acute to obtuse at apex; inflorescence with more than 2 verticils	**3**
3	Upper lip of calyx at least 3× longer than the other lobes, lanceolate and acuminate; nutlets verrucose	***C.pengbelensis***
–	Upper lip of calyx not much longer than the other lobes, ovate, rounded, obtuse to subacute, never acuminate; nutlets smooth	**4**
4	Lower lip of calyx broadly ovate-elliptic, strongly curving upwards and almost closing the throat; lateral lobes of calyx much longer than broad, acute, convergent, inconspicuous	**5**
–	Lower lip of calyx linear to oblong, not curving upwards except near tip; lateral lobes of calyx as long as broad, truncate to rounded, not convergent, conspicuous	**8**
5	Corolla 4–15 mm long; pedicel 2–4 mm long; verticils in fruit 12–15 mm broad	**6**
–	Corolla 10–22 mm long; pedicel 4–10 mm long; verticils in fruit 20–45 mm broad	**7**
6	Perennial with lower part of shoot lignified; stem prostrate to ascending or erect; corolla (8–)10–15 mm long, with lower lip 5–8 mm long; leaf blade subentire to shallowly crenate	***C.calaminthoides***
–	Annual; stem mostly erect; corolla 4–7 mm long, with lower lip 2–4 mm long; leaf blade crenate to serrate	**C.monostachyussubsp.monostachyus**
7	Lower lip of calyx curving upwards at a right angle to the tube, tightly appressed on the upper lip and closing the throat; calyx tube without long hairs	***C.mannii***
–	Lower lip of calyx curving upwards at an obtuse angle, not touching the upper lip and not closing the throat; calyx tube with long patent hairs	***C.shirensis***
8	Pedicel (4–)5–10 mm long at fruiting	**9**
–	Pedicel 1–5 mm long at fruiting	**11**
9	Shrub with persistent lignified, branching shoots; rootstock without tubers	***C.autranii***
–	Herb, mostly with a single short-lived shoot; rootstock with tubers (but these rarely collected)	**10**
10	Cymes pedunculate, 7–11(–19)-flowered, with two divergent cincinni with rachis elongating to 20 mm in fruit; leaf blade 6–8.5 cm long, margin crenate	***C.hildei***
–	Cymes sessile, ca. 5-flowered; rachis ca. 2 mm long, not divided in two cincinni; leaf blade 1.5–4.0 cm long, margin sharply serrate	***C.homblei***
11	Cymes with a 5–15 mm peduncle; cincinni diverging at a right angle, with rachis zigzagging in fruit; inflorescence axis subglabrous; leaf blade almost rounded, reniform, obtuse to rounded at apex	***C.zigzag***
–	Cymes sessile or with peduncle 1–2 mm; cincinni less strongly diverging, straight to slightly undulate; inflorescence axis pubescent to puberulent; leaf blade variable in shape, but neither rounded nor reniform	**12**
12	Perennial, with a rhizome (this sometimes thin) or tubers; lower part of stem lignified	**13**
–	Annual, stem not lignified, with fibrous root, lacking a rhizome or tubers	**16**
13	Flower bud and calyx beige tomentose; leaf blade truncate at base; stem hairs antrorse	***C.marunguensis***
–	Flower bud and calyx pubescent, not tomentose; leaf blade attenuate at base; stem hairs retrorse or spreading	**14**
14	Plant often not forming an inflorescence or this ill-developed; tubers plentiful; cultivated or escaped	***C.rotundifolius***
–	Inflorescence normally developed; tubers lacking or scarce; native species of natural vegetation	**15**
15	Rhizome thin, not lignified; stem slender, < 2 mm thick at base, often rooting at nodes near base, mostly unbranched; lower part of stem and petiole villose, with patent hairs > 2 mm long; leaf blade membranous, ovate, shortly decurrent on petiole; cincinni rachis up to 6 mm long in fruit; on moist soil in W DR. Congo	***C.brazzavillensis***
–	Rhizome thick, lignified; stem > (2–)3 mm thick at base, not rooting at nodes, often branching from base; lower part of stem base and petiole with hairs retrorse to spreading < 2 mm long; leaf blade thick, trullate, decurrent over most of petiole length; cincinni rachis up to 20 mm long in fruit; on dry soil, widespread	***C.welwitschii***
16	Bracts persisting at all inflorescence nodes	**17**
–	Bracts caducous or, rarely, persisting at lower nodes	**18**
17	Leaf blade 3–10 cm long, mostly obtuse at apex; inflorescence with 15–30 verticils; bracts longer than broad, 2–3 mm broad	***C.chevalieri***
–	Leaf blade 0.9–2.2 cm long, acute at apex; inflorescence with 4–11 verticils; bracts as long as broad, 3–5 mm broad	***C.collinus***
18	Inflorescence axis with indumentum of appressed retrorse hairs only, without glandular hairs or long patent hairs; cincinni rachis elongating to 1–12 mm in fruit	***C.bojeri***
–	Inflorescence axis with indumentum comprising short papilliform hairs, short microglandular hairs and sparse long multicellular patent hairs; cincinni rachis elongating to 5–50 mm in fruit	***C.heterotrichus***

#### ﻿﻿Group 6. Corolla blue, purple, mauve, pink or white, not yellow; inflorescence lax at least in lower half, the axis conspicuous between most of the cymes; if condensed, then only in upper half or globose, not a dense conical spike; flowers pedicellate; calyx with upper lobe narrowly triangular, ovate to obovate, often broader and differing in shape and size from lower four lobes, lobes not all subulate and spinescent; lower calyx lobes free, not fused into a lip; cymes of two flowers or more

**Table:** 

1	Inflorescence of 1–2 globose very congested verticils; leaf blade 0.6–0.9 cm broad	***C.globosus***
–	Inflorescence verticils more numerous, less congested; leaf blade (0.3–) 0.9–12 cm broad	**2**
2	Upper lobe of calyx not very different in size and shape from the other lobes, all narrowly triangular-lanceolate, often curving upwards	**3**
–	Calyx upper lobe ovate, elliptic, obovate, oblong, very different in size and/or shape from the other four lobes	**7**
3	Calyx with yellow sessile glands; sinus between lower calyx lobes deeper than sinus between the other lobes; corolla 3–9 mm long	**4**
–	Calyx with red sessile glands; sinus between the lower calyx lobes no deeper than the other sinuses; corolla 8–25 mm long	**5**
4	Leaf petiolate; corolla 5–9 mm long	***C.succulentus***
–	Leaf sessile; corolla 3–5 mm long	***C.cylindraceus***
5	Leaf blade broadly ovate to almost round, rounded at apex, succulent; calyx tube markedly curving upwards, with all lobes pointing upwards	***C.tetradenifolius***
–	Leaf blade ovate-elliptic, acute at apex, not succulent; calyx tube almost straight or slightly curving, with lobes not all pointing upwards	**6**
6	Calyx 8–11 mm long, at least 2× longer than the pedicel, with red glands; plant leafy at flowering; inflorescence with adjacent verticils almost touching	***C.mirabilis***
–	Calyx 4–6 mm long, slightly exceeding pedicel, with pale glands; plant mostly leafless at flowering; inflorescence with adjacent verticils widely spaced	***C.defoliatus***
7	Fruiting calyx bearded inside throat, with nutlets obscured; calyx pointing downwards after anthesis; pedicel 3–7 mm long	**8**
–	Fruiting calyx glabrous to slightly pubescent inside throat, not bearded; calyx patent or downwardly pointing at fruiting; pedicel length variable	**9**
8	Corolla 5–7 mm long; calyx contracted at throat and gibbose ventrally; annual	***C.kivuensis***
–	Corolla 8–22 mm long; calyx neither contracted nor gibbose; perennial	***C.barbatus***
9	Fruiting pedicel (5–)7–15 mm long, as long as or longer than calyx (consider the longest pedicels)	**10**
–	Fruiting pedicel 1–5(–6) mm long, shorter than or equalling calyx	**16**
10	Plant with at least a few leaves at flowering	**11**
–	Plant wholly leafless at flowering	**14**
11	Leaf blade 0.7–2.5 cm broad; petiole 0–0.6 cm long	**12**
–	Leaf blade 1–7 cm broad; petiole (0.3–)0.7–7 cm long	**13**
12	Leaf axils with globose propagules; leaf blade narrowly elliptic, acute at apex; verticils 2–6-flowered	***C.kapatensis***
–	Leaf axils lacking propagules; leaf blade elliptic to obovate, obtuse at apex; verticils 8–16-flowered	***C.decimus***
13	Calyx 4–5(–6) mm long, with red sessile glands; verticils (4)12–20-flowered	***C.alpinus***
–	Calyx 6–7(–10) mm long, with yellow sessile glands; verticils 4–6(12)-flowered	***C.sylvestris***
14	Calyx tube strongly curving, with lobes pointing upwards; shrub > 1.2 m high	***C.penicillatus***
–	Calyx tube straight to slightly curving, with lobes not all pointing upwards; herbaceous perennial < 1.2 m high	**15**
15	Verticils 2–6-flowered; pedicel 6–10 mm long	***C.kapatensis***
–	Verticils 10–16-flowered; pedicel 10–15 mm long	***C.buchananii***
16	Cymes pedunculate (at least lowermost ones); pedicel not inserted on inflorescence axis	**17**
–	Cymes sessile; pedicels generally inserted on nodes of main inflorescence axis or on a very short cyme rachis	**21**
17	Calyx 8–11 mm long, with lobes subequal, all acute; leaves markedly discolorous	***C.mirabilis***
–	Calyx 4–8.5 mm long, with upper lobe different in shape from the other four lobes, rounded to obtuse; leaf blade not discolorous	**18**
18	Stem with stiff spinescent bristles; corolla 7–9 mm long	***C.seretii***
–	Stem lacking stiff spinescent bristles; corolla 8–18 mm long	**19**
19	Lower calyx lobes oblong, obtuse-rounded at tip; leaf margin irregularly sharply serrate	***C.thyrsoideus***
–	Lower calyx lobes acute; leaf margin regularly serrate	**20**
20	Corolla ca. 18 mm long; verticils ca. 10-flowered; leaf blade cordate at base	***C.frederici***
–	Corolla 8–14 mm long; verticils 10–25-flowered; leaf blade obtuse to rounded at base	***C.tenuicaulis***
21	Upper half of inflorescence spike-like, with verticils touching; corolla 3–5 mm long; sinus between the lower middle calyx lobes markedly deeper than the other sinuses	***C.cylindraceus***
–	Upper half of inflorescence not spike-like, with verticils not touching (except uppermost ones); corolla > 5 mm long; sinus between the lower middle calyx lobes no deeper than the other sinuses	**22**
22	Pedicel 1–2 mm long; upper calyx lobe horizontal, cucullate, not decurrent, oblong, rounded to apiculate at apex; corolla 4–9 mm long; calyx with pale sessile glands	***C.amboinicus***
–	Pedicel (1–)3–6(–9) mm long; upper lobe of calyx variable in shape, but never horizontal and cucullate; corolla 6–22 mm long; calyx with red sessile glands	**23**
23	Plant almost leafless at flowering; calyx upright, with all lobes narrowly triangular, acute, the lateral lobes closer to the upper lobe, forming a 3+2 pattern; bracts strikingly large (5–25 mm long) and conspicuous near inflorescence apex at anthesis	***C.lactiflorus***
–	Plant leafy at flowering; calyx patent to deflexed, with lateral lobes closer to the lower lobes, forming a 1+4 pattern; upper calyx lobe elliptic, generally curving upwards; bracts < 12 mm long	**24**
24	Leaves in 2–4 pairs, grouped in lower third of shoot, almost forming a rosette; leaf blade rounded at apex	***C.ruziziensis***
–	Leaves more numerous, more regularly spread along the shoot, not forming a rosette; leaf blade acute to rounded at apex	**25**
25	Petiole 0–1(–2) cm long, < 20% of leaf blade length; stem often purple-spotted; fruiting calyx downwards pointing	***C.maculosus***
–	Petiole 1–5 cm long, > 25% of leaf blade length; stem not spotted; fruiting calyx patent to deflexed	**26**
26	Calyx (3–)6–7 mm long, often downwards pointing, villose, with hairs more or less purplish tinged; upper lobe of calyx 2–3 mm wide	***C.lanuginosus***
–	Calyx 3–6 mm long, patent, pubescent, not villose, with white hairs; upper lobe of calyx 1–2 mm wide	**27**
27	Leaf blade membranous; base of leaf blade broadly obtuse to rounded and then shortly decurrent on the petiole; apex of leaf blade generally acute; bracts 3–12 mm long; in dense forest and savannah on moist soil	***C.alpinus***
–	Leaf blade succulent; base of leaf blade truncate to subcordate, not decurrent on petiole; apex of leaf blade obtuse to rounded; bracts 1–3 mm long; in savannah on dry soil	***C.hadiensis***

### ﻿﻿Annotated checklist and descriptions

In the following checklist, synonym citation is limited to: i) the names used in the Flore du Rwanda (Troupin and Ayobangira 1985) and the Flore du Parc national Albert (Robyns 1947), ii) names based on type materials collected in DR. Congo, Rwanda, Burundi, iii) new synonyms and iv) newly-lectotypified names; for a full account of synonymy in the genus *Coleus*, see Paton et al. (2019). For each taxon, specimens are cited according to the phytogeographic regions of Central Africa, as used in the Flore d’Afrique centrale. For the species of the *Coleusbojeri* complex, which were sunk into *C.bojeri* in earlier floras, the overall distribution in Africa has been explored and, when necessary, new country records are testified by one voucher specimen per country. When our taxonomic treatment departs from recent floras, a concise justification is provided.

Extensive descriptions are included only for: i) the new taxa, ii) the taxa not included in recent floras (Paton et al. 2009, 2013; Paton 2022) (*C.brazzavillensis*, *C.celsus*, *C.conglomeratus*, *C.frederici, C.globosus* and *C.tenuicaulis*), iii) the taxa in the *C.bojeri* complex, the circumscription of which is amended here. In the descriptions, the length of cincinni refers to the rachis of the cyme, excluding pedicels and flowers.

#### ﻿ Coleus affinis

Taxon classificationPlantaeLamialesLamiaceae

(Gürke) A.J.Paton, Phytokeys 129: 14. 2019.

68585888-09D9-51C7-BFF7-3D04415107FD

= Pycnostachysspeciosa Gürke, in H.G.A.Engler, Pflanzenw. Ost-Afrikas, C: 345. 1895., non Coleusspeciosus Baker f. Type: Kenya/Tanzania, east shore of Lake Victoria, *A.Fischer 499* (lectotype B designated by Bruce (1940), destroyed).

##### Type.

Tanzania, Muansa [Mwanza], May 1892, *F.Stuhlmann 4693* (holotype B destroyed; isotype K [K000405978] fragment).

##### Description.

Paton et al. (2009: 397), as *Pycnostachysspeciosa* Gürke.

##### Distribution.

Rwanda to E Tropical Africa.

##### Habitat and ecology.

Savannah, ca. 1350 m elev. in Rwanda (1150 -1750 m elev. elsewhere).

##### Additional specimen.

Rwanda, Akagera, environs du lac Ihema, Jun. 1960, *G.Bouxin & M.Radoux 1959* (BR).

##### Note.

This species was not recorded in Rwanda by Troupin and Ayobangira (1985).

#### ﻿ Coleus alpinus

Taxon classificationPlantaeLamialesLamiaceae

Vatke, Linnaea 37: 322. 1872.

5ED759F0-B702-5FFB-84AE-AA246959CB11

≡ Plectranthusalpinus (Vatke) Ryding, Bot. Jahrb. Syst. 121: 147. 1999. Type: Ethiopia, Amara, Edda Jesus near Dawra Tabor [Debra Tabor], 25 Sep 1863, *G.W.Schimper s.n*. (lectotype BM [BM000513299], designated by Ryding (1999b); isolectotype JE, US, W).
= Coleusassurgens Baker in D.Oliver & auct. suc. (eds.), Fl. Trop. Afr. 5: 428. 1900.
≡ Plectranthusassurgens (Baker) J.K.Morton, J. Linn. Soc., Bot. 58: 267. 1962. Type: Ethiopia, Begemder, Aug 1863. *G.W.Schimper s.n.* (holotype K [K000431867]).
= Coleuslebrunii Robyns, Bull. Jard. Bot. État Bruxelles 17: 73. 1943. Type: DR. Congo, Ruwenzori, Butagu Valley, Nov 1931. *J.Lebrun 4579* (holotype BR [BR0000008910035], [BR0000006262631]; isotype K [K000431884]).
= Coleuswittei Robyns Bull. Jard. Bot. État Bruxelles 17: 74. 1943. Type: DR. Congo, Kamatembe, R. Bishkishaki, 16 Apr 1934. *G.F.de Witte 1551* (holotype BR [BR0000006262914], [BR0000006262631]).

##### Description.

Paton et al. (2009: 296), Paton et al. (2013: 245), as *Plectranthusalpinus* (Vatke) Ryding.

##### Distribution.

Nigeria to Ethiopia and south to Malawi.

##### Habitat and ecology.

Savannah, river banks, marshland, mountain forest, *Erica* shrubland, fallow fields, 1300–2700 m elev.

##### Additional specimens.

DR. Congo, ***Lacs Edouard et Kivu***, Ruwenzori, Lamia, 14 May 1914, *J.Bequaert 4262* (BR); Ruwenzori, 20 Dec 1949, *de Wilde 439* (BR); Masisi, Bohenda, 7 May 1957, *R.Gutzwiller 904* (BR); Kabare, Birava, 20 Aug 1959, *Meurillon 791*; Lwiro, marais Lushala, 14 Jun 1958, *G.Troupin 7495* (BR); Nyamuragira, N-hang, 21 Aug 1954, *H.U.Stauffer 106* (BR).

Rwanda, Entre Mutura et Kanama, 14 Jun 1984, *J.Lejoly 84/353* (BRLU); Préfecture Gisenyi, route Muramba-Rutsiro, Ramba, 14 Jun 1974, *C.Nuyt 292* (BR); Gisovu, 15 Jun 1978, *J.Raynal 20580* (BR); Rangiro, Kirambo, 4 Jun 1981, *G.Troupin 16268* (BR); R. Mukungwa, waterfalls near Rwaza, 23 Feb 1972, *P.Van der Veken 9510* (BR).

Burundi, Nkaka, 1 Mar 2003, *E.Bizuru 728* (BRLU).

##### Note.

New species record for Burundi.

#### ﻿ Coleus amboinicus

Taxon classificationPlantaeLamialesLamiaceae

Lour., Fl. Cochinch.: 372. 1790.

CB47FB9E-705D-5E90-90AE-52EED6198403

≡ Plectranthusamboinicus (Lour.) Spreng., Syst. Veg. 2: 690. 1825. Type: Rumphius plate in Herb. Amb. 5, t. 102/2. 1750 (lectotype designated by Cramer [1978]); Thailand, Pai District, Mae Hong Son, 25 May 1921. *F.Kerr s.n*. (epitype BM, designated by Suddee et al. [2004]).
= Coleusamboinicusvar.violaceus Gürke, Bot. Jahrb. Syst. 19: 210. 1894. Type: Tanzania, Moshi District, Dschallasee [Lake Chala], Jun 1893, *G.Volkens 321* (syntype B destroyed; isosyntype BM, K [K000431981]), & Pangani River, Jul 1893, *G.Volkens 487* (syntype B destroyed; isosyntype BM, K).

##### Description.

Paton et al. (2009: 320), Paton et al. (2013: 262), as *Plectranthusamboinicus* (Lour.) Spreng.

##### Distribution.

Kenya to South Africa, Arabian Peninsula, India.

##### Habitat and ecology.

Steppe, ca. 925 m elev.

##### Additional specimens.

DR. Congo, ***Lacs Edouard et Kivu***, Kabare, bords du Lac Kivu, Sep 1914, *J.Bequaert 5522* (BR).

##### Note.

Apparently, a rare species in Central Africa, known from a single collection. Other collections previously reported to this species were errors.

#### ﻿ Coleus articulatus

Taxon classificationPlantaeLamialesLamiaceae

(I.M.Johnst.) A.J.Paton, Phytokeys 129: 21. 2019.

BB77BC05-AF0C-587A-8B94-DB22DCC7EAC8

≡ Symphostemonarticulatus I.M.Johnst., Contr. Gray Herb., n.s., 73: 38. 1924. Type: Angola, east of Cuanza [Coanza] R., 23 Sep 1923, *A.G.Curtis* 309 (holotype GH [GH00002146]).
= Plectranthushockii De Wild., Repert. Spec. Nov. Regni Veg. 11: 542. 1913. Type: DR. Congo, Plateau de Shinkwari (Manika), 1911, *A.Hock s.n.* (lectotype BR [BR0000006263287], designated here).

##### Description.

Paton et al. (2009: 285), Paton et al. (2013: 233), as *Plectranthushockii* De Wild.

##### Distribution.

SW Tanzania, DR. Congo, Malawi, Zambia, Angola.

##### Habitat and ecology.

Savannah and steppic savannah, often in highlands and in frequently burnt places, miombo woodlands, occasionally on copper rich soil, 1480–1810 m elev.

##### Additional specimens.

DR. Congo, ***Haut-Katanga***, Kansenia, 13 Aug 1933, *H.Lynes s.n*. (BR); Upemba, entre Mabwe et Mukana, 1 Oct 1948, *G.F.de Witte 4402* (BR); Upemba, env. Mukana, 1949, *G.F.de Witte 7025* (BR); Env. Lubudi, 1937, *D.Cabu 49* (BR, WAG); Upemba, entre Masombwe et Lusinga, 20 Sep 1948, *W.Robyns 3638* (BR); Env. Fungurume, Kwatebala, 22 Oct 2006, *E.Kisimba*, *L.Saad & F.Malaisse 50* (BR); Entre Nzilo et Kansenia, 6 Sep 1956, *P.Duvigneaud & J.Timperman 2626* (BRLU).

##### Note.

Lectotypification of *Plectranthushockii* De Wild. De Wildeman (1913b) cited two syntypes, i.e. *A.Hock s.n.* (syntype BR [BR0000008109453]; isosyntype K), DR. Congo, Haut-Katanga, bords de la Dilemba, 1911 & *A.Hock s.n.* (syntype BR [BR0000006263287]), Plateau de Shinkwari (Manika), 1911. The specimen [BR0000008109453] departs somewhat from the protologue in having the pedicel glabrous under the joint (protologue: “breviter tomentoso”), while [BR0000006263287] is slightly puberulous below the joint; moreover, [BR0000006263287] has a thick woody rootstock, quite typical of the species; it is selected as the lectotype.

#### ﻿ Coleus autranii

Taxon classificationPlantaeLamialesLamiaceae

Briq., Bull. Herb. Boissier 2: 129. 1894.

32FAABDF-CAA2-5737-B244-C389689C29B9

≡ Calchasautranii (Briq.) P.V.Heath, Calyx 5: 160. 1996.
≡ Solenostemonautranii (Briq.) J.K.Morton, Novon 8: 266. 1998.
≡ Plectranthusautranii (Briq.) Erhardt, Götz & Seybold, Grosse Zander 2: 1825. 2008. Type: Ethiopia, 6 Sep 1952, *G.W.Schimper 693* (holotype G [G00435189]; isotype K, P [P00450781], [P00450782], [P00450783]).
= Coleussilvaticus Gürke, Bot. Jahrb. Syst. 19: 219. 1894.
≡ Solenostemonsilvaticus (Gürke) Agnew, Upland Kenya Wild Fl.: 640. 1974. Type: Tanzania, W Usambara Mts, Shagai Forest, near Sunga, *R.B.Drummond & J.H.Hemsley 2593* (neotype K, designated by Paton et al. [2009]).

##### Description.

Paton et al. (2009: 326), Paton et al. (2013: 265), as *Plectranthusautranii* (Briq.) A.J.Paton.

##### Distribution.

Ethiopia, Central Africa, East Africa to South Africa.

##### Habitat and ecology.

Mountain forests, clearings with *Hagenia*, scrub, *Senecio-Philippia* vegetation, riparian forest, 1000–3200 m elev.

##### Additional specimens.

DR. Congo, ***Forestier central***: Route Baudouinville [Moba] à Katele (?), Sep 1922, *S.de Giorgi 67* (BR). ***Lacs Edouard et Kivu***, Karisimbi, versant sud, riv. Bikuri, 27 Feb 1935, *G.F.de Witte 2265* (BR); Entre Kasindi et Lubango, W du Lac Edouard, Jan 1932, *J.Lebrun 4737* (BR); Wimbi, 26 km S Lubero, 22 Jul 1937, *J.Louis 4658* (BR). ***Haut-Katanga***, Upemba, 16 Apr 1949, *G.F.de Witte 6141* (BR); Marungu, Mashini, 28 Jun 1957, *P.Duvigneaud 3750Co* (BRLU).

Rwanda, Kareba, versant sud du Karisimbi, 10 Oct 1974, *P.Auquier 4513* (BR); Dorwa, Dec 1932, *A.Becquet 194*; Route Pindura-Ibigugu, km 88, 9 Jan 1980, *D.Bridson 158* (BR, K, WAG); Route Astrida [Butare]-Shangugu, km 71, crête Congo-Nil, 9 Sep 1959, *M.Reynders 403* (BR); Forêt de Nyungwe, env. Uwinka, 26 May 1981, *G.Troupin 16256* (BR, WAG).

Burundi, Bukeye, Mt Teza, 19 Jun 1971, *J.Lewalle 6007* (BR); Bubanza, Mugomero, 12 Jun 1981, *M.Reekmans* 10601 (BR, WAG).

#### ﻿ Coleus barbatus

Taxon classificationPlantaeLamialesLamiaceae

(Andrews) Benth. ex G.Don in J.C.Loudon, Hort. Brit.: 483. 1830.

9BC81FCE-4273-5F2E-85C6-F864A4E087CB

≡ Plectranthusbarbatus Andrews, Bot. Repos. 10: t. 594. 1810. Type: Illustration of cultivated material in Bot. Rep. 9, t. 594. 1809 (lectotype); ERITREA, Dekemehare, 5 Sep 1954, *J.W.Colville 47* (epitype K [K000431890], designated by Ryding [1999a]).

### ﻿﻿Key to the varieties of *Coleusbarbatus*

**Table:** 

1	Trailing, ascending or erect perennial, < 0.6(–1.5) m high; leaf blade elliptic, 0.9–7 × 0.3–3 cm, cuneate at base	**C.barbatusvar.barbatus**
–	Shrub up to 4.5 m high; leaf blade ovate, 1.5–20 × 0.8–11 cm, base rounded to subcordate	**C.barbatusvar.grandis**

#### ﻿ Coleus barbatus var. barbatus

Taxon classificationPlantaeLamialesLamiaceae

150767BE-5C43-599B-ACF3-8D9D3671735C

##### Description.

Paton et al. (2009: 338), as PlectranthusbarbatusAndrewsvar.barbatus.

##### Distribution.

Eritrea to N Tanzania and E DR. Congo, Arabian Peninsula, Indian Subcontinent to SC China.

##### Habitat and ecology.

Savannah, 1000–1400 m elev.

##### Additional specimens.

DR. Congo, ***Lacs Edouard et Kivu***, Rutshuru, haute vallée de la Fuku, Dec 1937, *J.Lebrun 9133* (BR).

Rwanda, Mohasi-See [Lac Mohazi], Aug 1907, *J.Mildbraed 671* (B destroyed); territ. Miumba, Mutara, near Mimuli, *G.Troupin 2781* (BR).

##### Notes.

1. New record to DR. Congo.

2. The type variety is apparently rare in Central Africa.

#### ﻿ Coleus barbatus var. grandis

Taxon classificationPlantaeLamialesLamiaceae

(L.H.Cramer) A.J.Paton, Phytokeys 129: 24. 2019.

8F0B60D5-3C20-55EF-B581-A6409EB2B3D4

≡ Coleusgrandis L.H.Cramer, Kew Bull. 32: 556. 1978.
≡ Plectranthusbarbatusvar.grandis (L.H.Cramer) Lukhoba & A.J.Paton, Kew Bull. 58: 915. 2003. publ. 2004. Type: Sri Lanka, Sita Eleiya. 3 Oct 1972, *L.H.Cramer 3869* (holotype PDA; isotype K [K000820136], US).
= Coleuskilimandschari Gürke, Abh. Königl. Akad. Wiss. Berlin 1891: 359. 1892.
≡ Plectranthuskilimandschari (Gürke) H.I.Maass in R.Mansfeld, Verz. Landwirtsch. Gärtn. Kulturpfl. 3: 1136. 1986. Type: Tanzania, Kilimanjaro, Marangu, Jun 1893, *G.Volkens 427* (neotype K [K000975992] designated by Paton et al. [2009]; isoneotype G [G00435190]).
= Plectranthusneochilus sensu Troupin & Ayobangira, Fl. Rwanda 3: 339. 1985. non (Schltr.) Codd.

##### Description.

Paton et al. (2009: 339), Paton et al. (2013: 273), as Plectranthusbarbatusvar.grandis (L.H.Cramer) Lukhoba & A.J.Paton.

##### Distribution.

NE & E Tropical Africa to DR. Congo.

##### Habitat and ecology.

Savannah, rock outcrops, fallow fields, often planted in villages for hedgerows, 800–2250 m elev.

##### Additional specimens.

DR. Congo, ***Lac Albert***, Mont Aboro (Djugu), 26 Feb 1958, *D.Froment 348* (BR); Mt Ota, NE of Gote (Mahagi), 12 Jul 1945, *R.Germain 3975* (BR); ***Lacs Edouard et Kivu***, Beni, 7 Apr 1914, *J.Bequaert 3447* (BR); Masisi, Bugobe, 18 Jan 1957, *R.Gutzwiller 702* (BR, WAG); Mulungu, 20 Sep 1940, *F.L.Hendrickx 1393* (BR); Entre Walikale et Kalihe, May 1932, *J.Lebrun 5349* (BR).

Rwanda, Kigali, Buliza, Nov 1932, *A.Becquet 222* (BR); Butare, Muhura, 17 May 1972, *L.Van Puyvelde & A.Kayonga 4* (BR); Rubona, 17 Apr 1958, *G.Michel 5283* (BR); Route Usa-Astrida [Butare], km 72.5, 11 Feb 1960, *F.L.Hendrickx 7776* (BR, BRLU, LSHI). BURUNDI, Bukeye, Mt Teza, 26 Jun 1969, *J.Lewalle 3839* (BR); *M.Reekmans 3060* (BR); Ngozi, Remera, 26 Feb 1976, *M.Reekmans 4808* (BR); Irubura, 31 May 1926, *W.Robyns 2394* (BR).

#### ﻿ Coleus batesii

Taxon classificationPlantaeLamialesLamiaceae

(Baker) A.J.Paton, Phytokeys 129: 25. 2019.

AC670DAC-11EB-5849-B4A3-4CF0589D4026

≡ Pycnostachysbatesii Baker in D.Oliver & auct. suc. (eds.), Fl. Trop. Afr. 5: 386. 1900. Type: Cameroon, Efulen, 11 Sep 1885, *G.L.Bates 372* (holotype K [K000405951]; isotype BM [BM000884040]).

##### Description.

Paton et al. (2009: 406), as *Pycnostachysbatesii* Baker.

##### Distribution.

Cameroon to SW Uganda and DR. Congo.

##### Habitat and ecology.

Rainforest, riparian forest, 750–1300 m elev.

##### Additional specimens.

DR. Congo, ***Forestier Central***, Riv. Mangbana, afffl. Djuma-Semliki, 14 Mar 1955, *G.F.de Witte 12002* (BR); Confluent Mamudjoma-Djuma, 7 Mar 1955, *G.F.de Witte 12042* (BR, K); Urega (Maniema), Jun 1932, *J.Lebrun 5604* (BR, K); Ituri, between Lodjo and Ituri River crossing, 27 Jan 2011, *B.Bytebier et al. 3403* (BR, EA, EPU, K); ***Lac Albert***, Ituri, Mont Hoyo, route forestière vers le poste de Hoyo, 2 Aug 1975, *S.Lisowski 40875* (POZG).

#### ﻿ Coleus betonicifolius

Taxon classificationPlantaeLamialesLamiaceae

(Baker) A.J.Paton, Phytokeys 129: 26. 2019.

B04BFE07-BFB3-5463-A5E8-274B1CDCB4EB

≡ Plectranthusbetonicifolius Baker, Bull. Misc. Inform. Kew 1895: 72. 1895. Type: Zambia, Fwambo, *A.Carson 79* (lectotype K [K000431993], designated by Pollard and Paton [2009]).

##### Description.

Paton et al. (2009: 307) as *Plectranthusbetonicifolius* Baker.

##### Note.

*C.betonicifolius* is variable in leaf shape; variation is clearly bimodal; varietal rank is proposed here.

### ﻿﻿Key to the varieties of *Coleusbetonicifolius*

**Table:** 

1	Leaf blade ovate, abruptly contracted at base	**C.betonicifoliusvar.betonicifolius**
–	Leaf blade narrowly elliptic to almost linear, progressively attenuated at base	**C.betonicifoliusvar.kasomenensis**

#### ﻿ Coleus betonicifolius (Baker) A.J.Paton var. betonicifolius

Taxon classificationPlantaeLamialesLamiaceae

9A82346E-A316-5B06-83FA-64E0DBBC8E6F

##### Description.

Paton et al. (2009: 307), Paton et al. (2013: 252), as *Plectranthusbetonicifolius* Baker, restricted to specimens with leaf blade ovate, 1–3 cm wide, abruptly contracted into the petiole.

##### Distribution.

Tanzania, DR. Congo, Zambia, Malawi and Angola.

##### Habitat and ecology.

Steppic savannah, on seasonally flooded soil (dilungu and dambo), often on highlands, from 1100 to 1880 m elev.

##### Additional specimens.

DR. Congo, ***Haut-Katanga***, Upemba, vallée de la Musibari (?), 25 Apr 1959, *J.de Wilde 738* (BR); Upemba, Mukana, 14 Apr 1947, *G.F.de Witte 2494* (BR, WAG); Upemba, tête de source de la Mubale, 16 Jan 1948, *G.F.de Witte 3247* (BR); Muhila, riv. Mutungulu, 16 May 1971, *F.Malaisse 1355* (BR); N’konda, piste Nasondoye-Kasofu, 4 Jan 1983, *M.Schaijes 1774* (BR).

Burundi, Mosso Urundi, Butetsi (?), 9 May 1952, *G.Michel & J.Reed 1905* (BR).

#### ﻿ Coleus betonicifolius (Baker) A.J.Paton var. kasomenensis

Taxon classificationPlantaeLamialesLamiaceae

(De Wild.) Meerts & A.J.Paton, comb. et stat. nov.

B89A13EB-9025-524C-B045-A3C0D4DAD064

urn:lsid:ipni.org:names:77347688-1

Coleus kasomenensis (in protologue as “*kasonememsis*”) De Wild., in Rep. Spec. Nov. Reg. Veg. 11: 515. 1913. Type: DR. Congo, Elisabethville [Lubumbashi], Mar. 1912, *H.Homblé 246* (in protologue “*206*”) (lectotype BR [BR0000006258283], designated here). (Basionym)
= Coleushockii De Wild., Repert. Spec. Nov. Regni Veg. 11: 514. 1913. Type: DR. Congo, Haut-Katanga, vallée de la petite Luembe, lieux humides, Feb1910, *A.Hock s.n.* (holotype BR [BR0000008907790]), syn. nov.
= Coleusbaumii Gürke, in O. Warburg (ed.), Kunene-Sambesi Exped.: 357. 1903. Type: Angola, Cuito, 14 Dec 1899, *H.Baum 544* (holotype B destroyed; isotype BM [BM000910139], E, G [G00435191], HBG [HBG518672], K [K000431990], M [M0104724], S [S-G-1537], W [W1901-0006713]), syn. nov.
= ?Leocuslyratus A.Chev., J. Bot. (Morot) 22: 126. 1909. Type: Guinea, Fouta-Djalon, Mt. Tinka, near Dalaba. Sep-Oct 1907, *A.Chevalier 18824* (holotype P [P00541249], syn. nov.

##### Description.

Differs from the type variety by the leaf blade narrowly elliptic to almost linear (< 1 cm wide), progressively attenuate at base (vs. ovate, abruptly contracted at base).

##### Distribution.

DR. Congo, Angola, Zambia.

##### Habitat and ecology.

Savannah, on seasonally flooded soil, marshland, often on sand, ca. 1050–1300 m elev.

##### Additional specimens.

DR. Congo, ***Kasaï***, Kapanga, 1933, *F.Overlaet 915* (BR); ***Bas-Katanga***, Route Kaniama-Kamina, 85 km from Kamina, 21 Dec 1957, *J.Brynaert 670* (BR); Kelambwe, 8 Dec 1948, *G.Kevers 228* (BR); ***Haut-Katanga***, Dilolo, dilungu de la Mangoa, 1 May 1959, *S.Risopoulos 1032* (BR); Kasumbalesa, 26 Mar 1951, *A.Schmitz 3509* (BR); Kipopo, 13 Mar 1960, *A.Schmitz 6890* (BR).

Angola, **Lunda Norte**, S of River Luovwa [Lovua?], 18 Jan 1938, *E.Milne-Redhead 4205* (BR, K); **Bié**, Serpa Pinto, 26 Apr 1906, *J.Gossweiler 2679* (COI).

Zambia, District Chingola, 19 Apr 1954, *D.B.Fanshawe 1128* (K); Kawambwa District, Kalungweshi River, 26 Apr 1957, *H.M.Richards 9446* (K).

##### Notes.

1. *Coleushockii* De Wild. was wrongly interpreted as a synonym of *Coleusbuchananii* (Baker) Brenan by Paton et al. (2019), due to confusion with *A.Hock s.n.* (BR [BR0000008109613]), which is the type specimen of *C.kisanfuensis* De Wild. (= *Coleusbuchananii* (Baker) Brenan). The protologue and the original materials of *Coleushockii* De Wild. clearly refer to *C.betonicifolius.* See also note under *C.buchananii*.

2. The spelling of the epithet in the protologue is “*kasonememsis*”, obviously a typo; the locality cited in the protologue is “Kasomenia” and on the specimen label “Kasonema”. The current name of the locality is Kasomeno (10°45'S, 28°16'E); therefore, we correct the epithet to “*kasomenensis*” following Art. 60.1. and Rec. 60D.1. of the ICN.

3. Lectotypification of *Coleuskasomenensis.*De Wildeman (1913a) cited four syntypes: *T.Kassner 2555* (syntype BR; isosyntype E, K), DR. Congo, Haut-Katanga, Kasomenia (in protologue; Kasonema on specimen label) [Kasomeno], 10 Mar 1908, & *H.Homblé 246* (in protologue “*206*”) (syntype BR [BR0000006258283] & [BR0000006258917]), Elisabethville [Lubumbashi], Mar 1912, & *J.Bequaert 382* (syntype BR [BR0000009824553] with a collecting label, [BR0000009824577] without a collecting label), Welgelegen, 2 May 1912 and *J.Bequaert 564* (syntype BR [BR0000009824553]), Welgelegen, dembo, 2 May 1912. All these specimens match the protologue; *H.Homblé 246* [BR0000006258283] is selected as the lectotype because it is the only specimen with De Wildeman’s handwriting on the label.

4. Material of *C.betonicifolius* from Guinea and Sierra Leone is similar to the type of Leocuslyratus A.Chev. and resembles var. kasomenensis, but the leaves are much larger than material from the remainder of the distribution (Pollard and Paton 2009); further work is needed.

#### ﻿ Coleus bojeri

Taxon classificationPlantaeLamialesLamiaceae

Benth., Labiat. Gen. Spec.: 52. 1832.

CB15AEF4-78DB-56DD-8D6A-84ECEFBA0F16

Figs 1A, B , 2B

= ?Coleusdewevrei Briq., Bull. Soc. Roy. Bot. Belgique 37: 71. 1899. Type: DR. Congo, s.l., s.d. (see note), *A.Dewèvre 1092A* (holotype BR [BR0000006262211], [BR0000006262525]).

##### Type.

Madagascar, Emirna, Betani-Mena, *W.Bojer s.n.* (lectotype P [P00541359], designated by Hedge et al. (1998); isolectotype W [W0002279]).

##### Description.

Annual herb, 0.3–0.9(–1.2) m high, more or less aromatic; rootstock fibrose, without tubers. Stem erect or, rarely, ascending, quadrangular, branched, indumentum mostly of retrorse and patent eglandular hairs, in the inflorescence only of retrorse, appressed hairs, without glandular hairs or long patent hairs. Leaves opposite, spreading, petiolate, occasionally with small leaves in the axils; petiole (0.5–)1.0–4.0 cm long, ciliate, more or less flat and narrowly winged in the upper half; blade ovate-triangular to narrowly ovate, 1.5–4.5(–7.5) × 1.0–4.0(–6.2) cm, base broadly cuneate, then shortly attenuate into the petiole, rarely subcordate, apex rounded to obtuse or subacute, ca. 4–5 pairs of secondary veins, occasionally impressed on upper surface, sparsely pilose to shortly pubescent on the upper surface, pubescent on veins on the lower surface (either spreading pilose or with antrorse appressed hairs), margin crenate or rarely undulate and subentire. Inflorescence terminal, lax, (4–)12–42 × 1.2–2.5 cm in fruit, with 9–30 verticils spaced 5–25(–30) mm, cymes sessile, ascending, ca. (7–)9–25-flowered, cincinni ca. 1–2 mm long at anthesis, elongating in fruit to 1–7(–12) mm, pedicels 1–3(–4) mm long, inserted eccentrically in front of calyx upper lobe, curved at tip, bracts ovate, 2–4 mm long, acute to acuminate, cucullate, forming an inconspicuous coma, early caducous. Flower: calyx shortly pubescent, with red sessile glands, 1.5 mm long at anthesis, 3–5 mm long in fruit, tube shortly cylindrical, truncate, slightly constricted at throat, upper lip often purplish tinged, ovate to obovate, curved upwards, obtuse to rounded, occasionally subacute, apiculate, 1.5–2 mm long, lateral lobes of lower lip truncate, ca. 1 mm, middle lobes of lower lip fused in a linear bidentate lobe 2–3 mm long. Corolla blue or violet, rarely white, with red sessile glands, 8–9 mm long, tube 3–4 mm, sigmoid, lower lip 4–6 mm long, 1.5–2.5 mm deep, enclosing stamens and style, stamens fused, style bifid. Nutlets globose, brown, red speckled, 0.8–1 mm.

**Figure 1.**
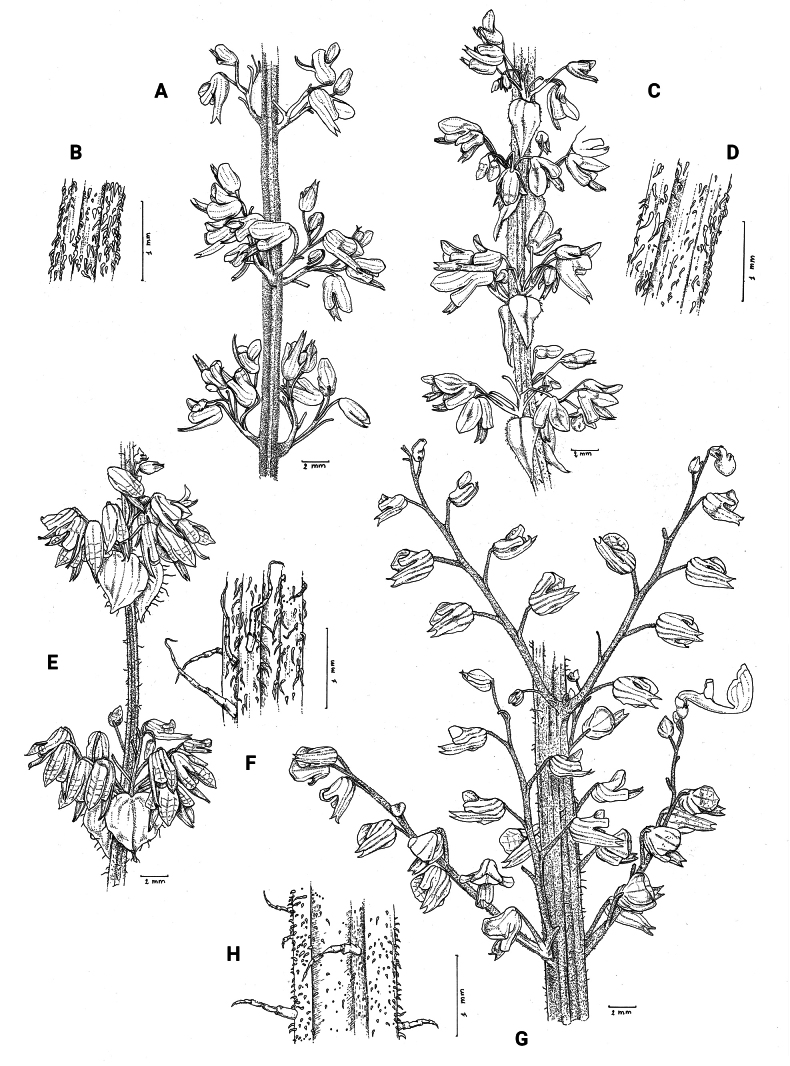
*Coleusbojeri* complex, details of inflorescence architecture and stem pubescence in four species **A, B***Coleusbojeri* Benth. (*T.Sperry 179*) **C, D***Coleuschevalieri* Briq. (*P.Gérard 4297*) **E, F***Coleuscollinus* Robyns & Lebrun (*J.Lebrun 9041*) **G, H***Coleusheterotrichus* Briq. (*M.Micha 300*). Drawn by Hilde Orye.Scale bars: 1 mm (**B, D, F, H**); 2 mm (**A, C, E, G**).

##### Distribution.

Senegal, Mali, Sierra Leone, Ivory Coast, Ghana, Togo, Benin, Nigeria, Cameroon, Congo, Gabon, Sudan, South Sudan, Ethiopia, Uganda, Kenya, Tanzania, Mozambique, Malawi, Zambia, Zimbabwe, Angola, Madagascar.

##### Habitat and ecology.

Rainforest, dry woodland, savannah, fallow fields, rock outcrops, occasionally an epiphyte, 370–1900 m elev.

##### Additional specimens.

DR. Congo, ***Bas-Congo***, 15 km N Kisantu, 5 Apr 1944, *R.Germain 2043* (BR); **Kasaï**, Bilala, 22 Feb 1937, *J.Gillardin 210* (BR); Route Kindu-Katako Kombe (km 64), 14 Apr 1959, *P.Bamps 475* (BR); ***Bas-Katanga***, Lofu, 26 Mar 1947, *L.Van Meel 1283* (BR); ***Forestier Central***, Namoya, near Shapandi, 15 Apr 2008, *B.Bytebier & W.R.Q.Luke 2965* (BR); Secteur Bangengele, Lomami, 3.9 km NNE Katopa, 6 Apr 2015, *R.Gereau et al. 7407* (BR, MO); ***Ubangi-Uele***, Dendu, Sep 1921, *J.Claessens 1708* (BR); Bambesa, 31 Oct 1956, *P.Gérard 2392* (BR); ***Lac Albert***, Nioka, 1934, *F.Jurion in J.Claessens 215* (BR); Kasengi, Oct 1931, *J.Lebrun 4100* (BR); ***Lacs Edouard et Kivu***, Riv. Kahekavitiri, affl. de rive droite de la Mukandwe, près de Mutsora, 12 Jun 1954, *G.F.de Witte 10529* (BR); Ruwenzori, entre Mutwanga et le gîte Kalonge, 28 Dec 1977, *J.Lejoly 2233* (BR); ***Haut-Katanga***, Env. Elisabethville [Lubumbashi], 1923, *S.de Giorgi s.n.* (BR); Kumanua, 4 Feb 1976, *F.Malaisse 8963* (BR); Marungu, ravin de Kafwampa, May 1945, *P.J.J.Vanden Brande 279* (BR).

**Figure 2.**
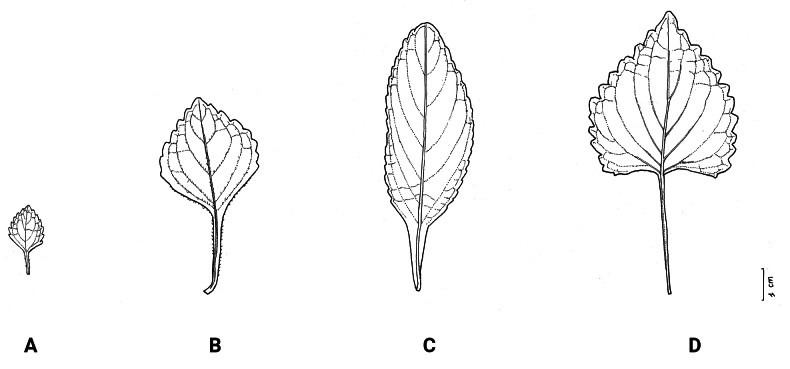
*Coleusbojeri* complex, leaf shape in four species **A***Coleuscollinus (J.Lewalle 1936)***B***Coleusbojeri (T.Sperry 179)***C***Coleuschevalieri (P.Gérard 4297)***D***Coleusheterotrichus* (*P.Quarré 3159*). Drawn by Hilde Orye. Scale bar: 1 cm.

Rwanda, Lac Kivu, route Nyamasheke-Kibuye, km 19, 29 Mar 1972, *G.Bouxin & M.Radoux 1526* (BR).

Burundi, Mosso, 7 Jun 1952, *G.Michel 2584* (BR); Bururi, Resha, 26 May 1981, *M.Reekmans 10379* (BR, MO, US).

Senegal, Massif de Kita, 7 Oct 1943, *P.Jaeger 32* (K).

Mali, Kayes Region, Kenieba Cercle, Falea Rockgate Mine, 24 Oct 2012, *W.R.Q. Luke & Sanogo 15850* (EA, K, IER).

Sierra Leone, West of Kambia N.P. 3 Nov 1963, *J.K.Morton SL74* (SL, GC, FHI, K, WAG).

Ivory Coast, Northern part of Bouna Reserve, Téhini, about 40 km E of Ouangofétini, 24 Aug 1963, *W.J.J.O.de Wilde 752a* (K, WAG).

Ghana, Gambaga Scarp, grown from seed at Legon, 3 Apr 1961, *J.K.Morton s.n.* (GC, K).

Togo, 15 km S of Dapaon, 10°44'N, 0°12'E, 11 Oct 1977, *Ern et al*. *1546* (B, K). Benin, Borgou, Tchaourou, Ouari-Maro, 9°00'N, 2°15'E, 24 Aug 1999, *B.Sinsin 2900* (K, WAG). Nigeria, NE State, Mambilla Plateau, 27 May 1972, *J.D.Chapman* 2835 (FHI, K). Cameroon, Balowa, Balikambat, 13 Sep 1960, *S.Gillett 2* (K).

Congo (Brazzaville), Lekoumou Préfecture, Zanaga Project, near Yakatopema/MPD Congo SA camp. 2°46'40"S, 13°35'52"E, 4 Oct 2009, *M.Cheek 15095* (IEC, K).

Gabon, Haut-Ogooué, Plateaux Batéké National Park, Mpassa River Drainage, Harunga forest, 2°12'36"S,14°01'56"E, 2 Mar 2003, *G.Walters & R.Niangadouma 1194* (K, MO).

Sudan, Jebel Marra, Colol, 23 May, 1968, *A.A.Kamil 1088* (K).

South Sudan [Bongo Land], Oct 1877, *G.Schweinfurth 2490* (K; type of *Plectranthusbongensis* Baker). Ethiopia, Joje, 10 km S of middle Abay Bridge, 10°12.70'N, 36°59.29'E, 11 Oct 2010, *I.Friis, W. Abebe, E.Getachew 13412* (AAU, K).

Uganda, Ajai Wildlife Reserve, 10 Dec 2001, *J. Kalema JK 1094*, (K).

Kenya, 4 km from Webuye on road to Kitale, 0°38'N, 34°46'E, 10 Oct 1981, *M.G.Gilbert & M.Tadessa 6583* (EA, K).

Tanzania, Kagera, Bukoba Rural District, Minziro Forest Reserve, Nyokabanga, kaiyamba Hill, 01°02'20"S, 31°32'04"E, 18 May 2001, *L.Festo, W, Bayona, & W.Wibard 1485* (K, MO).

Mozambique, Sussundenga Distr., E slopes of Chimanimani Mts, West of Dombe, Mevumosi River Valley, streamside, 27 Apr 1974, *G.Pope & T.Müller 1330* (K).

Malawi, Rumphi Distr., Rukuru Bridge, junction to Rumphi to Livingstonia, 29 Apr 1971, *Pawek 4756* (K, MAL).

Zambia, Chingola, 5 Aug 1955, *D.B.Fanshawe 2411* (K, NDO).

Zimbabwe, Mrewa, Shawanae River, 23 Apr 1956, *H.Wild 4806* (K, SRGH).

Angola, Lunda Norte, near Kasamba Village ca.59 km NNW of Capaia on road to Carumbo, 7°50'15"S, 20°01'46"E, 4 Apr 2013 *D.J.Goyder & I.Darbyshire 7209* (K).

Madagascar, N of Ankazobe, Mar 1930, *M.R.Decary 7364* (K, P).

##### Notes.

1. *C.bojeri* is defined here in a narrower sense compared to recent floras (Paton et al. 2009, 2013, 2022), excluding *C.chevalieri*, *C.collinus* and *C.heterotrichus*. It is restricted to specimens without persistent bracts (or rarely persistent at the two lowermost nodes), with short cincinni and stem indumentum mostly of retrorse hairs, without long patent hairs in the inflorescence.

2. *C.bojeri* is a polymorphic taxon possibly still in need of further splitting. One of the most distinctive morphs was named *Coleusplatostomoides*Robyns and Lebrun (1928) 362. (Type: *W.Robyns 2482* (holotype BR; isotype P), DR. Congo, Lac Kivu, baie de Sake, plaine de lave de Kateruzi, 10 Jun 1926). It differs in the shoot much branched from base, the longer cincinni (rachis up to 12 mm long in fruit) with closely spaced pedicels (ca. 15 flowers/cincinni) distichously arranged in a fish-bone pattern at fruiting; it has a distinctive ecogeographic distribution, restricted to volcanic lava in Kivu (e.g. Parc National Albert [Rumoka], 21 Apr 1945, *R.Germain 3785* & 3786 (BR, L); Territ. Beni, Kusolu, Oct 1938, *P.Gille 129* (BR); Beni, 31 Jul 1914, *J.Bequaert 5140* (BR); Parc National Albert [Virunga], Kahodju, Aug 1937, *J.Lebrun 6925* (BR, P); Entre le volcan Rumoka et la baie de Sake, Aug 1937, *J.Louis 4861* (BR, P); Plaine de lave près de Ngoma [Goma], Apr-May 1929, *H.Humbert 7901* (P). However, specimens showing one or several of these traits are occasionally found elsewhere (e.g. Kasaï, Territ. Dibaya, riv. Mwanzangoma, 9 Jan 1957, *L.Liben 2220* (BR); Uganda, *A.D.Poulsen, G.Eilu & D. Hafashimana 1260* [C, K, MHU]) and intermediates with typical *C.bojeri* are not rare in Kivu. Evidence from molecular markers is needed to further resolve the taxonomy of this difficult group.

3. The specimen *A.Léonard 2040* (Mikwati, territ. Walikale, 16 Dec 1958 [BR]), a subglabrous plant with very small (ca. 10 × 5 mm), acute leaves and a long calyx (6 mm in fruit), is probably a different taxon, but more materials are needed.

4. The specimen *Yona Mleci 19* (BRLU), collected on termite mounds in miombo woodland near Lubumbashi, has roots with numerous clavate tubers; it approaches *Coleusbotryosus*, differing in the caducous bracts; it could be a different taxon, but more materials are needed.

5. In Burundi, a few specimens are more or less intermediate between *C.bojeri* and *C.homblei* (e.g. Bururi, Lac Nyanza, route de Makamba, 19 Mar 1967, *J.Lewalle 1698* [BR]) and between *C.bojeri* and *C.collinus* (e.g. Munvugo, 3 km N Nyanza, 16 Apr 1978, *M.Reekmans 6920* (BR, LG).

6. The type specimen of *Coleusdewevrei* De Wild. is possibly a perennial plant, more or less intermediate between *C.bojeri* and *C.welwitschii* or *C.brazzavillensis*.

7. See observations under *C.homblei*.

#### ﻿ Coleus brazzavillensis

Taxon classificationPlantaeLamialesLamiaceae

A.Chev., Veg. Ut. Afr. Trop. Franç. 1: 124. 1905.

F54FD323-E443-5BA2-895B-030D1D384468

##### Type.

Republic Of Congo (Brazzaville), 1904, *A.Chevalier 11154* (holotype P [P00450785]).

##### Description.

Perennial herb 0.30–0.75 m high, not reported to be aromatic, with a thin creeping rhizome, tubers not collected in DR. Congo (present in Rep. Congo). Stem ascending, rooting at lower nodes, simple or more rarely sparingly branched, quadrangular, more or less villous, with long patent flexuous hairs up to 2 mm long, these occasionally sparse or restricted to nodes and short retrorse hairs, in the inflorescence with shorter patent or antrorse hairs (0.5 mm), intermingled with sparse much longer hairs and also with red sessile glands. Leaves opposite, petiolate, ascending to spreading, occasionally with smaller leaves in the axils; petiole 0.5–2.5 cm long, with flexuous patent hairs 2–3 mm long, these sometimes sparse, and short retrorse hairs, narrowly winged in upper half, blade ovate to ovate-elliptic, 1.5–8 × 1–3 cm, apex obtuse to acute, base cuneate and shortly attenuate, ca. 5 pairs of secondary veins, margin crenate, narrowly recurved, both surfaces pubescent with long erect to appressed hairs, especially on veins beneath and with red sessile glands beneath. Inflorescence lax, (4–)9–18 cm long, with 7–15 verticils spaced (5–)10–25(–45) mm, bracts ovate, ca. 5 × 3 mm, acuminate, forming an apical coma, deciduous, except often at 1–2 lowermost nodes where they are somewhat foliaceous and crenate; cymes sessile to very shortly pedunculate (1 mm), ascending, ca. 13-flowered, cincinni up to 6 mm long in fruit, pedicels 1–4 mm, inserted eccentrically behind calyx upper lobe. Flower: calyx shortly pubescent and with red sessile glands, ca. 1.5 mm long in flower, up to 4.5 mm long in fruit, tube shortly cylindrical, constricted at throat, truncate, upper lobe ovate, recurved, subacute, purplish tinged, ciliolate, lateral lobes truncate, middle lobes of lower lip fused in a linear lip ca. 3 mm long, with two subulate points; corolla ca. 9–14 mm long, tube ca. 4 mm long, strongly sigmoid, lower lip ca. 5–7 mm long, 2.5–3 mm deep, cucullate, upper lip 2 mm long, with a broad gap between the two lips. Nutlets pale brown, globose, ca. 0.9 mm diam., smooth.

##### Distribution.

Republic of Congo (Brazzaville), DR. Congo.

##### Habitat and ecology.

Marshland, wet savannah, grassy clearings, *Sphagnum* bogs, dry woodlands; 300–750 m elev.

##### Additional specimens.

DR. Congo, ***Bas-Congo***, Léopoldville [Kinshasa], 15 Apr 1915, *J.Bequaert 7341* (BR); Boko Mfumu Disu, 15 Nov 1948, *H.Callens 1911* (BR); Madimba, 2 Apr 1948, *P.Duvigneaud 609Ca* (BRLU); Entre Ngidinga et Kimvula, 12 Apr 1948, *P.Duvigneaud 703C* (BRLU); Boko sur chemin de fer, Apr 1932, *H.Vanderyst 29736, 29747, 29751* (BR); ***Kasaï***, Kwango, Mela, *H.Callens 1677* (BR); Panzi, 8 Feb 1950, *H.Callens 2185* (BR); Region of Luebo, s.d., *L.Achten 415a&b*; ***Forestier Central***, Entre Bokatola et Bikoro, Lac Léopold II [Lac Mai Ndombe], Sep 1930, *J.Lebrun 1445* (BR).

##### Notes.

1. New species record for DR. Congo.

2. *C.brazzavillensis* was hitherto a poorly-known species. It is superficially similar to *C.bojeri*, differing in being perennial. *C.brazzavillensis* is closely related to and, arguably, only a variety of *C.welwitschii*, differing in the much more slender rhizome, longer hairs on shoot and petiole, shorter cincinni, persistent bracts at lowermost verticils and its occurring in moist habitats; blade length/width ratio also tends to be greater than in *C.welwitschii*. Its range is restricted to W DR. Congo and Congo (Brazzaville).

3. *H.Callens 2185* (BR) has the indumentum of the stem much sparser than usual. *J.Lebrun 1445* (BR) is unusual in having persistent bracts at all verticils.

#### ﻿ Coleus buchananii

Taxon classificationPlantaeLamialesLamiaceae

(Baker) Brenan, Mem. New York Bot. Gard. 9: 43. 1954.

77EDAFA9-A6CB-582C-9BD5-CB7073034EB8

≡ Plectranthusbuchananii Baker in D.Oliver & auct. suc. (eds.), Fl. Trop. Afr. 5: 402. 1900. Type: Malawi, Shire Highlands, Nakajumbu, 15 Oct 1881, *J.Buchanan 365* (holotype K [K000431996]; isotype E [E00193487]).
= Coleuskisanfuensis De Wild., Contr. Fl. Katanga: 174. & Ann. Soc. Sci. Bruxelles 41(2): 48. 1921. Type: DR. Congo, Elisabethville [Lubumbashi], Sep 1911, *A.Hock s.n.* (lectotype BR [BR0000008730572], designated here), syn. nov.

##### Description.

Paton et al. (2009: 324), Paton et al. (2013: 263), as *Plectranthusbuchananii* Baker.

##### Distribution.

Tanzania to S Tropical Africa.

##### Habitat and ecology.

Dry woodlands, wooded savannah, steppic savannah, often on rocky soil, occasionally on copper-rich soil; 950–1550 m elev.

##### Additional specimens.

DR. Congo, ***Haut-Katanga***, Upemba, près de la Lufira, 30 Aug 1948, *G.F.de Witte 4222* (BR); Vallée de la Lofoi, près du village Nkonko, 3 Oct 1970, *S.Lisowski 23358* (POZG); Luiswishi, 21 Sep 1984, *F.Malaisse 13191* (BR); Région de Fungurume, colline “monde arabe”, 24 Jul 2007; *B.Senterre 4676* (BR); Tantara, autour de la mine, 3 Sep 1977, *R.Wechuysen 865, 866, 867* (BR).

Burundi, Bururi, 1951, *A.Becquet 2149* (BR); Bururi, route Makamba-Dunga, km 20, 21 Sep 1977, *M.Reekmans 6378* (BR).

##### Notes.

1. *Coleuskisanfuensis* De Wild. was unplaced in Paton et al. (2019), because the original materials had not been found. The materials were misfiled in BR as “*Coleushockii* De Wild.” (which is Coleusbetonicifoliusvar.kasomenensis (De Wild.) Meerts & A.J.Paton). The specimen [BR0000008109613] indeed bears a label handwritten by De Wildeman as “*Coleushockii* De Wild.”, but this is obviously a mistake, because the protologue of *C.kisanfuensis* clearly refers to *C.buchananii*, not to *C.betonicifolius*.

2. Lectotypification of *Coleuskisanfuensis* De Wild. De Wildeman (1921b) cited two syntypes, i.e. *A.Hock s.n.* (syntype BR [BR0000008109613]), DR. Congo, Kisanfu, Sep 1911 and *A.Hock s.n.* (syntype BR [BR0000008732835] and [BR0000008730572]), Elisabethville [Lubumbashi], Sep 1911. The three sheets match the protologue. We designate [BR0000008730572] as the lectotype because it does not bear the erroneous name *Coleushockii* De Wild.

#### ﻿ Coleus calaminthoides

Taxon classificationPlantaeLamialesLamiaceae

(Baker) A.J.Paton, Phytokeys 129: 30. 2019.

7DA35854-10D6-5B4A-A54F-62ACCD39689D

≡ Solenostemoncalaminthoides Baker in D.Oliver & auct. suc. (eds.), Fl. Trop. Afr. 5: 421. 1900. Type: Gabon, Gabon River, *G.Mann s.n*. (holotype K [K001615425]).

##### Description.

Paton (2022: 44). The materials from DR. Congo differ from the materials in the rest of the range in having the hairs on the stem either retrorse or antrorse (always antrorse out of DR. Congo).

##### Distribution.

Cameroon, Equatorial Guinea, Gabon, DR. Congo.

##### Habitat and ecology.

Coastal scrub on sandy soil; 0–100 m elev.

##### Additional specimens.

DR. Congo, ***Côtier***, Plateau de Tshikai, route Boma-Banana, 13 Apr 1960, *Compère 1863* (BR); Route Boma-Banana, entre Malemba et Matamba ma Kanzi, 13 Mar 1959, *J.Wagemans 2230* (BR); ***Mayumbe***, Luki, 5°34'50.73"S, 13°9'34.16"E, 6 Apr 2010, *S.Dessein 3222* (BR); Forêt de Moba [entre Luki et Boma], route de Boma, 5 Dec 1948, *C.Donis 2233* (BR); without locality, Aug-Sep 1899, *Tilman* in *Cabra 67* (BR).

##### Notes.

1. The species was identified in DR. Congo for the first time by A.J.Paton in 2021; it had been hitherto misidentified as *C.monostachyus*. The Congolese localities represent the southernmost limit of the species’ distribution range.

2. Paton et al. (2022) consider that *C.calaminthoides* differs from *C.monostachyus* in having antrorse hairs on the stem. However, this character is variable in DR. Congo, with *C. Donis 2233* having antrorse hairs, while the other specimens have retrorse hairs.

3. The specimen *Tilman* in *Cabra 67* (BR) is close to *C.calaminthoides*, except for the yellow corolla; it could be a different taxon.

#### ﻿ Coleus caninus subsp. flavovirens

Taxon classificationPlantaeLamialesLamiaceae

(Gürke) A.J.Paton, Phytokeys 129: 32. 2019.

09649D17-39E8-5180-B81D-737C2B7F928C

≡ Coleusflavovirens Gürke in H.G.A.Engler, Pflanzenw. Ost-Afrikas, C: 347. 1895.
≡ Plectranthuscaninussubsp.flavovirens (Gürke) A.J.Paton, Fl. Trop. E. Afr., Lamiac.: 345. 2009. Type: Tanzania, Dschallasee [Lake Chala], 1893, *G.Volkens 1771* (holotype B destroyed; isotype BR [BR0000021453960]).

##### Description.

Paton et al. (2009: 345), Paton et al. (2013: 274), as PlectranthuscaninusRothsubsp.flavovirens (Gürke) A.J.Paton.

##### Distribution.

Ethiopia to South Africa.

##### Habitat and ecology.

Savannah, often on dry or rocky soil; 780–1590 m elev.

##### Additional specimens.

DR. Congo, ***Lacs Edouard et Kivu***, Kabare, Lac Kivu, 21 Aug 1914, *J.Bequaert 5384* (BR); May ya Moto, 14 Nov 1934, *G.F.de Witte 2037* (BR); Plaine de la Ruzizi, Apr 1950, *R.Germain 6851* (BR); Ruindi, Sep 1937, *J.Lebrun 7750* (BR). Rwanda, Akagera, Rwisirabo, 21 Jan 1980, *D.Bridson 255* (BR, K, WAG); Kabare, Gabiro, May 1933, *A.Becquet 607* (BR); Rwabiega, Jan 1938, *J.Lebrun 9824* (BR); Akagera, Rwisirabo, 2 Jul 1978, *J.Raynal 20794* (BR, P); Utara, env. Nyagatare, 1 May 1958, *G.Troupin 7240* (BR).

Burundi, Rugombo, plaine de la Ruzizi, 20 May 1969, *J.Lewalle 3590* (BR); Plaine de la Ruzizi, 13 May 1978, *M.Reekmans 6988* (BR, WAG).

#### ﻿ Coleus celsus

Taxon classificationPlantaeLamialesLamiaceae

A.J.Paton, Phytokeys 129: 34. 2019.

154FB6DF-C794-5097-AFE5-3C2BA280474D

≡ Solenostemonrobustus Hiern, Cat. Afr. Pl. 1: 864. 1900., non Coleusrobustus (Hook.f.) A.J.Paton.
≡ Plectranthusrobustus (Hiern) A.J.Paton in Fl. Zambesiaca 8,8: 234. 2013. Type: Angola, Pungo Andongo, from Lombe to Condo, Mar 1857, *F.Welwitsch 5538* (lectotype BM [BM00564035] designated here; isolectotypes [BM 00564268], LISU [LISU220992], [LISU220993]).

##### Description.

Woody perennial herb, ca. 1.0–1.5 m high, more or less leafless at flowering; rootstock with tubers up to 10 × 5 cm. Stem erect, up to 1 cm thick, woody and cylindrical in lower half, striate, lenticellate, puberulous with short eglandular hairs, obtusely quadrangular in upper half, densely white pubescent with short antrorse hairs, branching only in the inflorescence. Leaves opposite, petiolate, ascending, beginning to fall off at time of flowering; blade yellowish-green, coriaceous to somewhat crassulescent, broadly ovate, 4.5–8.5(–14.0) × 3.5–6.0(–9.0) cm long, base broadly cuneate, obtuse to rounded and then shortly attenuate into the blade, apex rounded to acute or shortly acuminate, margin crenate, not recurved, upper surface subglabrous to puberulous, lower surface shortly pubescent on veins, ca. 6–8 pairs of secondary veins; petiole 0.5–2.0(–3.0) cm long. Inflorescence 20–30 cm long, pyramidal-paniculiform, with 5–7 pairs of opposite ascending branches ca. 1.5–7 cm long, spaced ca. 5 cm, each terminating in 1–4 condensed ca. 1.5 cm long racemes, with tomentose to villous rachis; flowers solitary in the axil of each bract, arranged helicoidally around axis, bract ovate, ca. 2 mm long, villous on back, pedicel in fruit (8–)18 mm long, jointed ca. 2–5 mm above base and breaking at joint at maturity, leaving persistent base on rachis, pubescent, adnate eccentrically to calyx; calyx campanulate, beige to fulvous villous at first, ca. 5 mm long at anthesis, fruiting calyx (9–)13–14 mm long, coriaceous, tube campanulate, pubescent, with pale sessile glands, throat slightly oblique, lobes ciliate, upper lobe ovate-triangular, slightly curved, not decurrent, acute, ca. 2 mm long, lateral lobes of lower lip triangular, ca. 3 mm long, middle lobes of lower lip narrowly triangular ca. 4 mm long. Corolla purplish-pale blue with pale sessile glands, tube strongly sigmoid 7–8 mm long, upper lip ca. 3 mm long separated from lower one by a 3 mm gap, lower lip 9–11 mm long, 4–5 mm deep, cucullate, with two small auricles near base, anther pouch-like, style entire. Nutlets light brown, red speckled, smooth, broadly ovoid, ca. 2 mm.

##### Distribution.

Angola, DR. Congo.

##### Habitat and ecology.

Wooded savannah, steppe; 1000–1670 m elev.

##### Additional specimens.

DR. Congo, ***Kasaï***, Kwango, Village Shamafuka (15 km S de Bwana Mutombo), 1 Apr 1948, *P.Duvigneaud 970E* (BRLU).

##### Notes.

1. New species record for DR. Congo. The Congolese locality represents the northernmost limit of the species’ distribution range.

2. Lectotypification. *Solenostemonrobustus* Hiern was described most likely based on Welwitsch’s materials at BM. BM holds two sheets of *F.Welwitsch 5538*. We designate the sheet with complete collecting data on the label as the lectotype.

#### ﻿ Coleus chevalieri

Taxon classificationPlantaeLamialesLamiaceae

Briq., Mém. Soc. Bot. France 8: 287. 1917.

0836D976-E9D8-5E58-B6CD-CB8B60C89736

Figs 1C, D , 2C

= Coleusdelpierrei De Wild., Bol. Soc. Ibér. Ci. Nat. 19: 119. 1920. Type: DR. Congo, Ubangi-Uele, Van Kerkhovenville [Watsa], 1904, *A.Delpierre s.n*. (lectotype BR [BR0000006261559], designated here), syn. nov.

##### Type.

Central African Republic, Haut-Oubangui, Krébédjé (Fort Sibut), vallée de la moyenne Tomi, bord d’un sentier sur un plateau ferrugineux, 8 Nov 1902, *A.Chevalier 5662* (holotype P [P00450788], [P00450786]; isotype BR [BR0000006245498], G [G00437733]).

##### Description.

Annual herb, 0.15–0.70 m high, rootstock fibrose, without tubers. Stem erect, quadrangular, generally much branched from base, with short appressed retrorse hairs and red sessile glands, often purplish. Leaves opposite, ascending to spreading, petiole (0.2–)0.8–3.5(–4.5) cm long, narrowly winged in upper half, blade occasionally red spotted in the middle, ovate, narrowly ovate, 3–10(–11) × 1–4(–5.5) cm, mostly 2–3 times as long as broad, apex generally obtuse, rarely acute, base cuneate, shortly attenuate in the petiole, 4–8 pairs of secondary veins, occasionally impressed, margin crenate to obtusely serrate (teeth obtuse to rounded), subglabrous to very shortly pubescent above, shortly pubescent on veins beneath (antrorse or retrorse hairs). Inflorescence spiciform, (3–)5–12 cm long, 8 mm wide at anthesis (corolla excluded), congested, occasionally more lax, moderately dense at fruiting, up to 32 cm long, 10–15(–20) mm wide at fruiting, with (5–)15–30 verticils typically spaced 5–10 mm, the lowermost ones occasionally up 25 mm, bracts persistent, at first erect and cucullate, soon reflexed, ca. (1.5–)4–6 × (0.5–)2–3 mm long, ovate-triangular, sessile, acute to acuminate, occasionally almost caudate, ciliate, upper surface glabrous, lower surface sparsely pubescent and with red glands, forming a short apical coma; cymes sessile, ca. 11(–17)-flowered, cincinni ascending, elongating to 2–3(–6) mm, pedicels ca. 2–3 mm long. Flower: calyx shortly pubescent, with red sessile glands, ca. 2 mm long at anthesis, fruiting calyx subglabrous to shortly pubescent, 3.5–4 mm long, narrowly tubular, slightly constricted a throat, upper lip often purplish tinged, ovate-elliptic to obovate-elliptic, ca. 2.5 mm long, acute to more rarely rounded, recurving, not decurrent, lateral lobes truncate, ca. 1 mm long, median lobes of lower lip fused, linear, ca. 2 mm long, with two acute points, slightly curving upwards; corolla blue to pale mauve, with or without red sessile glands, ca. 8–12 mm long, tube 2.5–3 mm long, sigmoid, lower lip 3–7 mm long. Fruit: nutlets yellowish-brown with red speckles, globose ca. 0.8 mm diam., smooth.

##### Distribution.

Cameroon, Central African Republic, DR. Congo.

##### Habitat and ecology.

Savannah, dry woodland, fallow fields, often on lateritic crust, 300–1000 m elev.

##### Additional specimens.

DR. Congo, ***Bas-Katanga***, Haut-Lomami, Kaniama, 20 Apr 1947, *W.Mullenders 295* (BR); ***Forestier Central***, Eala, 28 Sep 1937, *G.Couteaux 338* (BR); Ikulu, 14 Feb 1940, *Freyne 4* (BR); Bambesa, 1936, *Pittery 424* (BR); Route Niangara-Kisangani, 55 km d’Isiro, vers Wamba, 20 Apr 1936, *J.Louis 1738* (BR); Asaka, 30 Oct 1923, *Nannan 623* (BR); ***Ubangi-Uele***, Doruma, 10 Sep 1933, *A.M.De Graer 101* (BR); Garamba, 10 Sep 1951, *H.De Saeger 1396* (BR, K); Uele-Nipoko, entre Niangara et Wamba, Jun 1931, *J.Lebrun 3199* (BR); Yakuluku, 29 Sep 1953, *P.Gérard 790* (BR).

Central African Republic, ***Haut-Oubangui***, Yalinga, 18 Sep 1921, *G.Le Testu 3256* (P); 60 km N of Bambari, 26 Nov 1928, *Ch.Tisserant 2296* (P); Oubangui-Chari, Koukourou, Sep. 1957, *J.Trochain 10648* (P).

Cameroun, Sabal Maba, 60 km NNE Tibati, 23 Sep 1963, *R.Letouzey 5920* (P).

##### Notes.

1. Within the *C.bojeri* complex, this species is easily recognised on account of the persistent bracts, long spiciform inflorescence with congested, closely-spaced verticils; the leaves tend to be obtuse, with blade more than 2× as long as broad, with numerous secondary veins, but these traits are more variable.

2. Robyns and Lebrun (1928) had already synonymised *C.delpierrei* De Wild. and *C.chevalieri* Briq.

3. Near the southern margins of the species’ distribution range, specimens intermediate between *C.chevalieri* and other species occur: e.g. *L.Pynaert 1702* (intermediate with *C.bojeri*); *H.Vanderyst 34394* (intermediate with *C.welwitschii*); *S.Risopoulos 146* (intermediate with *C.botryosus*); *L.Pynaert 1092, G.Couteaux 338* (BR) (intermediate with *C.brazzavillensis*).

4. Lectotypification of *Coleusdelpierrei* De Wild. De Wildeman (1920) cited two syntypes: *A.Delpierre s.n*. (BR [BR0000006261559]), DR. Congo, Vankerkhovenville [Watsa], 1904 & *A.Delpierre s.n*. (BR [BR0000006261887]), Niangara, 1904. Both match the protologue; [BR0000006261559] is selected because its inflorescence is better developed.

5. The most typical specimens of *C.chevalieri* occur in the Sudanian part of DR. Congo, in the region of Ubangi-Uele, where they grow on lateritic crust. In other regions of DR. Congo, *C.chevalieri* is often less typical and intermediates with other species of the *C.bojeri* complex occur.

#### ﻿ Coleus collinus

Taxon classificationPlantaeLamialesLamiaceae

Lebrun & L.Touss., Bull. Jard. Bot. État Bruxelles 17: 81. 1943.

0940B424-4993-5795-9676-D9539F996BDD

Figs 1E, F , 2A

≡ Solenostemoncollinum (Lebrun & L.Touss.) Troupin, Bull. Jard. Bot. Natl. Belg. 55: 299. 1985. Type: DR. Congo, Kivu, Rutshuru, Nov 1937, *J.Lebrun 8232* (holotype BR [BR0000006262990], [BR0000006262983]; isotype P).

##### Description.

Annual herb, aromatic, 0.15–0.40(–0.60) m, often in groups, rootstock fibrose, without tubers. Stem prostrate or ascending, more rarely erect, slender, occasionally rooting at lower nodes, quadrangular, with long patent multicellular eglandular hairs (ca. 1.5 mm long) and short appressed retrorse hairs, with red sessile glands, simple or much branched. Leaves opposite, spreading to ascending, petiole 0.2–1.1(–1.5) cm long, ciliate; blade ovate to trullate, 0.9–2.2 × 0.7–2.0 cm, base obtuse and then shortly attenuate into the petiole, apex acute, margin crenate, 4–6 teeth on either side, with sparse long hairs on both surfaces, dense red sessile glands on the lower surface, ca. 3 pairs of secondary veins. Inflorescence dense at anthesis, then lax at fruiting, simple, 2–5 cm long, ca. 6 mm wide (corollas excluded) at anthesis, elongating to 5–8 cm long, ca. 1 cm wide in fruit, with 4–11 verticils spaced 7–15 mm in fruit, cymes ca. 9-flowered, peduncle lacking, cincinni 0–4 mm long, ascending, pedicels 2–4 mm long, ascending, with short papilliform hairs, bracts broadly ovate, subsessile, 3–6 × 3–5 mm, foliaceous, the lowermost ones often serrate, persistent, reflexed, margin long ciliate, upper surface glabrous to papillate, lower surface pubescent, with red sessile glands. Flower: calyx shortly pubescent, with red sessile glands, 1–1.5 mm long at anthesis, fruiting calyx ca. 4 mm long, tube shortly campanulate, throat truncate, upper lobe obovate-elliptic, ca. 2 mm long, more or less acute, margin shortly ciliate, recurving, not decurrent, lateral lobes rectangular, truncate to rounded, median lobes of lower lip fused in a linear lip, straight, ca. 3 mm long, with two acute points. Corolla pale blue (rarely white), lower lip deep blue, ca. 2–4.5 mm long, tube 1–1.5 mm long, slightly curved, lower lip ca. 2 mm long, stamens included or occasionally exserted, anthers ca. 0.3 mm long. Fruit: nutlets yellowish to pale brown speckled with red, globose, ca. 0.8 mm diam., smooth.

##### Distribution.

Cameroon, DR. Congo, Rwanda, Burundi, Uganda, Kenya, Tanzania, Malawi, Zambia.

##### Habitat and ecology.

Savannah, fallow fields, rocky steppe, pastures, gravel, river banks; 1200–1800 m elev.

##### Additional specimens.

DR. Congo, ***Lacs Edouard et Kivu***, Luberizi, 1953, *A.Gilon 346* (BR); Rutshuru, Mt. Katale, Dec 1937, *J.Lebrun 9154* (BR, P); Rutshuru, 15 Apr 1937, *J.Ghesquière 4291* (BR); Mumosho, 8 May 1951, *J.F.Laurent 223* (BR).

Rwanda, Kibungo, Gahororo, environs de Zaza, 27 May 1970, *G.Bouxin & M.Radoux 2211* (BR, WAG); Rubona, Inéac, 28 Apr 1958, *G.Michel 5332* (BR, WAG).

Burundi, Bururi, Mosso, Bugiga, 21 May 1980, *M.Reekmans 9219* (BR, WAG); Ruyigi, Kitaba, 15 May 1981, *M.Reekmans 10333* (BR, MO, US, WAG); Gitega, Karuzi, 31 May 1981, *M.Reekmans 10517* (BR, WAG); Kitega Chefferie: Bweru, Environs Karuzi, colline Nyarusange, 16 May 1958, *van der Ben 2091* (BR).

Cameroon, Bamenda, Bambili, Bafut-Ngema, 19 Aug 1951, *E.Ujor FHI 29985* (FHI, K). Uganda, Serere Teso, Dec 1931, *Chandler 211* (K).

Kenya, Trans-Nzoia Distr., Kitale, 19 Sep 1961, *Verdcourt 3211* (EA, K).

Tanzania, Ngara distr., Nyakiziba, 26 Apr 1960, *R.E.S.Tanner 4890* (BR, K).

Malawi. Kondowe to Karonga, Jul. 1896, *A.Whyte s.n.* (K).

Zambia, By Katete River, where Great East Road crosses it, 16 Jan 1957, *J.M.Wright 124* (K).

##### Notes.

1. This species differs from *C.bojeri* in having persistent bracts, smaller corolla, narrower inflorescence. It often has ascending, somewhat flexuose shoots and smaller leaves, but these traits are more variable.

2. *C.collinus* is most likely a self-pollinating species, with corollas often not exceeding 1.5 mm long, often cleistogamous and anthers much smaller than in most *Coleus* species in Central Africa.

3. New species record for Cameroon, Uganda, Kenya, Tanzania, Zambia and Malawi.

#### ﻿ Coleus conglomeratus

Taxon classificationPlantaeLamialesLamiaceae

(T.C.E.Fr.) Robyns & Lebrun, Ann. Soc. Sci. Bruxelles, Sér. B 49: 105. 1929.

AF3D8F02-1FCC-5EBA-817E-8B4B219BCBC8

≡ Englerastrumconglomeratum T.C.E.Fr., Notizbl. Bot. Gart. Berlin-Dahlem 9: 72. 1924.
≡ Plectranthusconglomeratus (T.C.E.Fr.) Hutch. & Dandy, Bull. Misc. Inform. Kew 1926:481. 1926. Type: Togo, Sokode, Dec 1904, *H.Kersting 93* (holotype B destroyed; isotype K [K000431858] fragment).

##### Description.

Shrub or perennial woody herb, up to 2 m high, almost leafless at flowering, rootstock with tubers. Stem erect, sparingly branching, rounded in the lower part, quadrangular upwards, striate, lenticellate, purplish, densely covered with ca. 4 mm-long patent stiff bristles and with very short appressed hairs, young growth beige tomentellose, with dense retrorse hairs and more sparse patent hairs, these turning into bristles in older growth. Leaves opposite, patent, petiole 0.5–1.0 cm (up to 5.0 cm outside Central Africa), blade ovate to ovate-elliptic, 1–3 × 1–2 cm (up to 16 × 8 cm outside Central Africa), apex acute to subobtuse, base cuneate and shortly attenuate into the petiole, margin crenate, upper surface puberulous and with pale sessile glands, lower surface shortly pubescent on veins and with pale sessile glands. Inflorescence in short lateral and terminal subsessile panicles, each comprising 3 to 5 branches 2–5 cm long; rachis densely covered with short glandular hairs, flowers solitary in the axil of a bract, helicoidally arranged on the rachis, distally often subopposite; bracts narrowly elliptic, ca. 1 mm long, pedicels ascending, with thin patent eglandular hairs, ca. 3 mm long, eccentrically inserted on calyx; calyx tubular, with dense short glandular hairs and pale sessile glands, 2 mm long at anthesis, ca. 4.5 mm in fruit, tube slightly curved, upper lobe broadly ovate to obovate, subobtuse, somewhat curving upwards, slightly decurrent, lobes of the lower lip narrowly triangular, 1–1.5 mm long, the median ones slightly longer; corolla ca. 6 mm long (up to 10 mm outside Central Africa), yellow inside, suffused with purple outside, with pale sessile glands, tube ca. 4 mm long, funnel-shaped, lower lip equalling the tube, upper lip shorter, with a broad emarginate median lobe and two smaller rounded lateral lobes, stamens with filaments free for most of their length, anthers golden; style golden, stigma bifid. Nutlets somewhat compressed, brown, shiny, red speckled, ca. 1 mm.

##### Distribution.

Togo, Benin, Sierra Leone, DR. Congo.

##### Habitat and ecology.

Savannah on lateritic crust; 100–500 m elev.

##### Additional specimens.

DR. Congo, ***Ubangi-Uele***, Entre Businga et Banzyville [Mobayi-Mbongo], Jan 1931, *J.Lebrun 2061* (BR).

##### Notes.

1. New species record for DR. Congo.

2. The record in NW DR. Congo is remarkably disjunct, ca. 2000 km east of the nearest previously known locations in Togo. It had been misidentified in collections as *C.tetragonus*, on account of the bristles on the stem. It differs from the latter in the shorter pedicel (ca. 3 mm vs. 5–8 mm), shorter calyx (4.5 mm vs. 6–10 mm) and corolla suffused with purple.

#### ﻿ Coleus cylindraceus

Taxon classificationPlantaeLamialesLamiaceae

(Hochst. ex Benth.) A.J.Paton, Phytokeys 129: 37. 2019.

E6F4741E-5D11-54F7-AFD3-3422DC180D61

≡ Plectranthuscylindraceus Hochst. ex Benth. in A.P.de Candolle, Prodr. 12: 60. 1848. Type: Ethiopia, near Gapdia, ad rupes, 29 Nov1838, *G.W.Schimper II. 1113* (holotype K [K000431901], [K000431904], [K000431900]; isotype BM [BM000564023], E, FI [FI011097], [FI000844], G [G00435204], [G00435206], [G00435205], HAL [HAL0114463], M [M0104743], [M0104744], MPU [MPU015435], P [P00450713], [P00450711], [P00450712], PRE [PRE0235122-0], TUB [TUB009112], UPS, W).
= Plectranthusmontanus Benth. in N.Wallich, Pl. Asiat. Rar. 2: 17. 1830., non Coleusmontanus Hochst. ex Ces. Type: India, Deccan Peninsula, exact locality unknown (“Peninsula India Orientalis”), Herb. Wight in Wall. Cat. 2747B (lectotype K [K000820120]), designated by Suddee et al. (2004); isolectotype K-W [K001117007]).

##### Description.

Paton et al. (2009: 318), Paton et al. (2013: 260), as *Plectranthusmontanus* Benth.

##### Distribution.

Tropical and S Africa, Arabian Peninsula, S India.

##### Habitat and ecology.

Wooded savannah, rock outcrops, marshland, fallow fields; 1000–1400 m elev.

##### Additional specimens.

DR. Congo, ***Lacs Edouard et Kivu***, May ya Moto, 14 Nov 1954, *G.F.de Witte 2042* (BR).

Rwanda, Ngoma, Rukoma Nganza, Jun 1933, *A.Becquet 685* (BR); Préfecture Kibungo, Concession minière de Géorwanda-Rwinkwavu-colline dominant la plaine de Matinza, 21 May 1969, *G.Bouxin & M.Radoux 455* (BR); Préfecture Kigali, entre Karama et frontière du Burundi, à 14 km de Karama (Bugesera), 18 May 1978, *G.Troupin 15922* (BR); Biumba. marais Kibondo, 26 May 1955, *A.R.Christiaensen 895* (BR).

Burundi, Muyinga, Murehe, 30 May 1981, *M.Reekmans 10495* (BR, MO, WAG).

##### Note.

*G.F.de Witte 6449* (BR), from Haut-Katanga, Upemba National Park, is intermediate between *C.cylindraceus* and *C.succulentus*. See also note under the latter species.

#### ﻿ Coleus decimus

Taxon classificationPlantaeLamialesLamiaceae

(A.J.Paton) A.J.Paton, Phytokeys 129: 38. 2019.

586B7CA9-2DEA-5D2C-9FDE-00EE1A14BD1E

≡ Plectranthusdecimus A.J.Paton, Fl. Trop. E. Afr., Lamiac.: 324. 2009. Type: Zambia, Mbala (Abercorn) District, Kawimbe, 15 Dec 1956, *H.M.Richards 7288* (holotype K [K000431998]).

##### Description.

Paton et al. (2009: 324), Paton et al. (2013: 264), as *Plectranthusdecimus* A.J.Paton.

##### Distribution.

W Tanzania, Zambia to Angola.

##### Habitat and ecology.

Pionneer vegetation on rocky soil, often near rivers, ca. 1300 m elev.

##### Additional specimens.

DR. Congo, ***Haut-Katanga***, Tilwizembe, 8 May 1957, *P.Duvigneaud 3084L* (BRLU), Env. Kolwezi, rive gauche Musonoye, 13 Jan 2005, *F.Malaisse 16065* (BR); Près des chutes de la rivière Musonoi, 12 Mar. 1989, *M.Schaijes 4317* (BR).

##### Note.

New species record for DR. Congo.

#### ﻿ Coleus decurrens

Taxon classificationPlantaeLamialesLamiaceae

Gürke, Bot. Jahrb. Syst. 19: 215. 1894.

46CED321-FDCC-5649-804E-A500B4863DED

= Coleusvariifolius De Wild., Bol. Soc. Ibér. Ci. Nat. 19: 124. 1920. Type: DR. Congo, between Buta and Bima, 15 Oct 1905, *F.Seret 96* (lectotype BR [BR0000021453991], designated by Paton et al. 2009).

##### Type.

Cameroon, Buea, 1891, *P.Preuss 948* (holotype B destroyed; isotype COI [COI00005778], HBG).

##### Description.

Paton et al. (2009: 294) as *Plectranthusdecurrens* (Gürke) J.K.Morton, Paton (2022: 46) as *Coleusdecurrens* Gürke.

##### Distribution.

Nigeria to Uganda, Gabon, Republic of Congo, DR. Congo, Angola.

##### Habitat and ecology.

Rainforest, often seasonally flooded, riparian forest, river banks; 470–2000 m elev.

##### Additional specimens.

DR. Congo, ***Bas-Congo***, Without locality, 1904, *J.Gillet s.n*., & *J.Gillet 3783* (BR); ***Kasaï***, Panzi, galerie de la Makita, 18 Feb 1952, *H.Callens 3438* (BR); ***Forestier central***, Avakubi, 9 Jan 1914, *J.Bequaert 1853* (BR); Bongo, 26 Jul 1955, *C.Evrard 1512* (BR); Parc national de la Maïko, 45 km N of Lubutu, 2 Jun 1977, *J.Lejoly 1905C* (BR, BRLU); Yangambi, 7 km NW, 8 May 1936, *J.Louis 1834* (BR); Bolanda, 29 Oct 1913, *Nannan 85* (BR); Entre Buta et Banalia, Ambelati, 11 Jan 1926, *W.Robyns 1333* (BR); ***Ubangi-Uele***, entre Libenge et Géména, Dec 1930, *J.Lebrun 1866* (BR); ***Lac Albert***, Kilo, 28 Jun 1914, *J.Bequaert 4866* (BR); ***Lacs Edouard et Kivu***, Route Kibabi-Kikoma, km 50, 29 Apr 1958, *R.Pierlot 1943* (BR); Route Kavumu-Walikale, km 107, Irangi, 24 Oct 1959, *G.Troupin 10926* (BR); Kidedeya, près riv. Lusilube, 13 Sep 1955, *Vanschuytbroeck* in *de Witte 12687* (BR).

##### Note.

Two specimens (*J.Bequaert 6460; A.Leonard 3939*) are dwarf plants (< 20 cm), with small leaves and blade not long attenuated at base; they are either a juvenile form or a different taxon; more materials are needed.

#### ﻿ Coleus deflexifolius

Taxon classificationPlantaeLamialesLamiaceae

(Baker) A.J.Paton, Phytokeys 129: 39. 2019.

E9F0D73B-E598-5EFF-9EAF-E52B81A225B9

≡ Pycnostachysdeflexifolia Baker in D.Oliver & auct. suc. (eds.), Fl. Trop. Afr. 5: 381. 1900. Type: Kenya, Naivasha District, near Lake Elmenteita, 1893, *G.F.Scott Elliot 6756* (holotype K [K000405969]).

##### Description.

Paton et al. (2009: 394), as *Pycnostachysdeflexifolia* Baker.

##### Distribution.

E Tropical Africa.

##### Habitat and ecology.

Savannah on wet soil; 1300–2300 m elev.

##### Additional specimens.

DR. Congo, ***Haut-Katanga***, Kapemba, 1957, *P.Duvigneaud 3648Pyc* (BRLU).

##### Note.

New species record for DR. Congo.

#### ﻿ Coleus defoliatus

Taxon classificationPlantaeLamialesLamiaceae

(Hochst. ex Benth.) A.J.Paton, Phytokeys 129: 39. 2019.

D8731130-B4FA-56CF-A7C0-20E3E9E31BBA

≡ Plectranthusdefoliatus Hochst. ex Benth. in in A.P.de Candolle, Prodr. 12: 60. 1848. Type: Ethiopia, Jomara [Dschomara], 26 Dec 1839, *G.W.Schimper II.847* (holotype K; isotype BR [BR0000006250720], FI [FI000843], G [G00435210], [G00435211], HAL HAL0114469], LG, M [M0104742], MPU [MPU015434], P [P00450715], [P00450716], TUB [TUB009113], W).

##### Description.

Paton et al. (2009: 297), Paton et al. (2013: 246), as *Plectranthusdefoliatus* Hochst. ex Benth.

##### Distribution.

Eritrea to S Tropical Africa.

##### Habitat and ecology.

Savannah, steppe, riparian forest; 1600–2300 m elev.

##### Additional specimens.

DR. Congo, ***Lac Albert***, Nioka, Mont-Ri, 23 Nov 1957, *P.Bamps 66* (BR, POZG).

Burundi, Mugonga Manga, 14 Sep 1974, *P.Auquier 4087* (BR); Niambikiwe, Oct 1932, *A.Becquet 154* (BR); Route Bujumbura-Kitega, 19 Aug 1958, *A.Christiaensen 2477* (BR); Murambi, km 80 route Bujumbura-Butare, 20 May 1960, *F.L.Hendrickx 7962* (BR); Mubimbi, 24 May 1966, *J.Lewalle 827* (MO); Teza, 19 Jun 1971, *J.Lewalle 6028* (BR, WAG); Honga, 14 Oct 1971, *M.Reekmans 1055* (BR).

#### ﻿ Coleus descampsii

Taxon classificationPlantaeLamialesLamiaceae

(Briq.) A.J.Paton, Phytokeys 129: 40. 2019.

5E4F5F5B-F84B-52F5-9E71-27456784FE00

≡ Pycnostachysdescampsii Briq., Bull. Soc. Roy. Bot. Belgique 37: 63. 1899. Type: DR. Congo, Katanga, Lufonzo [Lufogo] R., Mar. 1896, *G.Descamps s.n.* (holotype BR [BR0000008910042]).

##### Description.

Paton et al. (2009: 388), as *Pycnostachysdescampsii* Briq.

##### Distribution.

Cameroon to W Tanzania.

##### Habitat and ecology.

Savannah, most often on moist, organic soil; 1000–1720 m elev.

##### Additional specimens.

DR. Congo, ***Haut-Katanga***, Marungu, 1940, *F.Jurion 343* (BR); Kasanga, 23 Mar 1908, *T.Kassner 2660* (BR); Territ. des Baanza, sur la Luvua, 18 May 1931, *F.Luxen 35* (BR); Environs de Lubumbashi, May 1945, *P.Quarré 8012* (BR); Kasambi, 20 Apr 1926, *W.Robyns 2037* (BR); Marungu, Kasiki, 12 Jun 1939, *P.J.J.Vanden Brande 247* (BR).

##### Note.

As already mentioned by Paton et al. (2009) in Tanzania, intermediates exist between *C.descampsii* and *C.parvifolius* (e.g. *S.Lisowski, F.Malaisse & J.-J.Symoens 11228* (POZG), Kundelungu, 25 Apr 1970).

#### ﻿ Coleus dewildemanianus

Taxon classificationPlantaeLamialesLamiaceae

(Robyns & Lebrun) A.J.Paton, Phytokeys 129: 40. 2019.

BBF2D1EB-EFA8-5B8E-AB40-7C14EB4786A4

≡ Pycnostachysdewildemaniana Robyns & Lebrun, Rev. Zool. Bot. Africaines 16: 352. 1928. Type: DR. Congo, Katanga, Munama, 1 Apr 1928 *P.Quarré 1143* (holotype BR [BR0000008910370], [BR0000008909732], [BR0000008910066]; isotype K, YBI).

##### Description.

Paton et al. (2009: 387), Paton et al. (2013: 315), as *Pycnostachysdewildemaniana* Robyns & Lebrun.

##### Distribution.

Tanzania to S Tropical Africa.

##### Habitat and ecology.

Shrub savannah, road verges, rocky steppe, miombo woodland, disturbed soil; 1100–1900 m elev.

##### Additional specimens.

DR. Congo, ***Haut-Katanga***, Kundelungu, 10 km NNE du Mont Kibwe wa Sanga, 26 Apr 1970, *S.Lisowski, F.Malaisse, J.-J.Symoens 11317* (POZG); Mont Mukuen, 1 Apr 1948, *A.Schmitz 1495* (BR).

Rwanda, Gahororo, env. Zaza, 27 May 1970, *G.Bouxin et M.Radoux 2223* (BR); Mahumna, commune Birenga, 4 Jul 1978, *B.Runyinya 864* (BR).

Burundi, Route Gitega-Karuzi, 27 May 1971, *J.Lewalle 5801* (BR, WAG); Kininya Mosso, 4 Jun 1952, *G.Michel et J.Reed 2384* (BR); Ruyigi, Gitwenge, 17 May 1978, *M.Reekmans 7056* (BR).

#### ﻿ Coleus duvigneaudii

Taxon classificationPlantaeLamialesLamiaceae

Meerts & A.J.Paton sp. nov.

813B02BF-E3D7-50A8-8164-2C20464B6560

urn:lsid:ipni.org:names:77347689-1

Fig. 3A–F

##### Type.

DR. Congo. Haut-Katanga, 15 km N de Mitwaba, steppe frais de plaine, 16 Jan 1960, *P.Duvigneaud 5082L* (holotype BRLU [BRLU0037795], isotype K).

##### Diagnosis.

Closely related to *Coleusfoliatus* (A.J.Paton) A.J.Paton, differing in the leaves ternate at all nodes, blade longer and narrower, mostly cuneate at base and acute at apex.

##### Description.

Perennial herb ca. 0.60 m high, with several shoots from a thick woody rootstock. Stem erect, branching only in the inflorescence, rounded near base, rounded to quadrangular in upper part, pubescent to hispidulous, with patent or ascending eglandular hairs and pale sessile glands. Leaves ternate, ascending, subsessile; blade narrowly elliptic, oblong-elliptic to almost linear, 6–9 × 0.5–1.3 cm, length/width ratio ca. 6–10, base mostly cuneate, but almost rounded in upper leaves, apex acute, ca. 8 pairs of secondary veins, margin slightly recurved, distantly serrate, both surfaces somewhat harsh to the touch, pubescent, more densely so on veins underneath, glandular punctate on upper surface, with dense red sessile glands on undersurface; petiole 0–1 mm long. Inflorescence lax to moderately congested at anthesis, laxer at fruiting, paniculiform, with opposite or ternate branches on the uppermost 4–6 nodes of the stem, branches ascending, stiff, 4–12 cm, the longest branches themselves branching; 1 flower in the axil of each bract, lowermost flowers opposite, rachis purplish tomentose, bracts narrowly ovate-elliptic, 3 × 1 mm, obtuse, thin, membranous, pedicels ascending, ca. 2–3 mm long at anthesis, up to 5 mm in fruit, densely pubescent over whole its length, with an inconspicuous joint in the upper third, breaking at the joint at maturity, occasionally not breaking, obliquely adnate to calyx base and eccentrically inserted in front of upper lobe, thickened at fruiting. Flower: calyx ca. 3 mm long at anthesis, densely brownish to purplish pubescent, with eglandular and glandular hairs (these with cup-shaped apical cell in herbarium) and pale sessile glands, campanulate to tubular in fruit, ca. 8–9 mm long, teeth subequal, triangular, acute, the middle lobes of lower lip slightly longer (3 mm long) and more sharply pointed. Corolla blue to purplish, ca. 14 mm long, tube strongly sigmoid, ca. 6 mm long, upper lip 3 mm long, lower lip 8 mm long, 3 mm deep, cucullate, acute at tip, curving upwards, with pale sessile glands and purplish glandular hairs, enclosing stamens and style; anther pouch-like, 1 mm long; style entire. Nutlets ovoid, ca. 1.5 mm long, brown.

**Figure 3.**
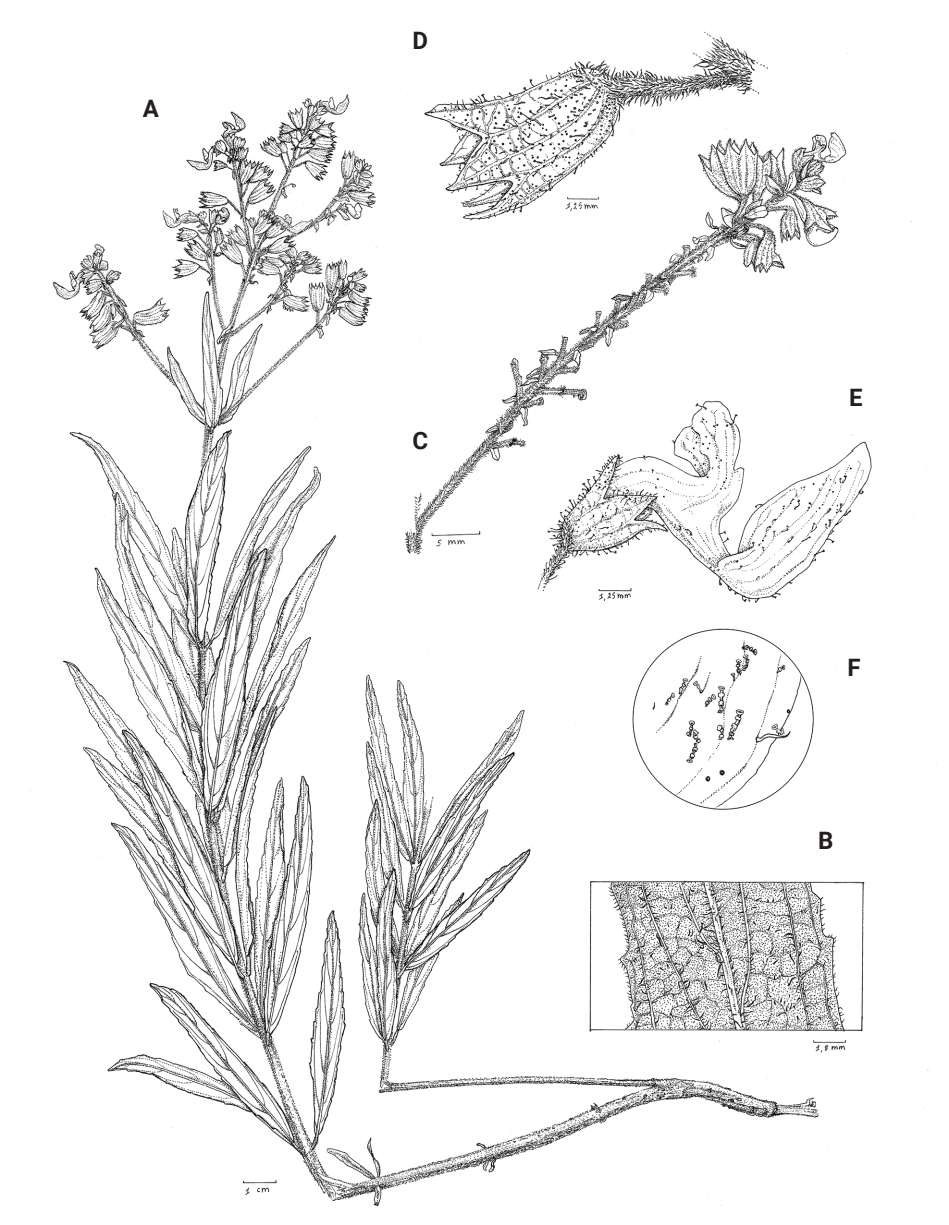
*Coleusduvigneaudii* Meerts & A.J.Paton **A** habit **B** detail of leaf blade undersurface **C** inflorescence branch **D** fruiting calyx **E** flower **F** detail of corolla pubescence (*P.Duvigneaud 5080L*). Drawn by Hilde Orye. Scale bars: 1 cm (**A**); 1.8 mm (**B**); 5 mm (**C**); 1.25 mm (**D, E**).

##### Etymology.

Dedicated to Paul Duvigneaud (1913–1991), Belgian botanist, who made important contributions to the knowledge of the flora and the vegetation of Katanga (DR. Congo).

##### Distribution.

Endemic of SE DR. Congo (Haut-Katanga); Mitwaba and Kundelungu Plateau.

##### Habitat and ecology.

Savannah, steppic savannah, often on rocky soil; 1500–1700 m elev.

##### Additional specimens.

DR. Congo, ***Haut-Katanga***, Sud du point Kisonsa, savane herbeuse, 24 Mar 1954, *R.Desenfans 5443* (BRLU); id., *R.Desenfans 5451* (BRLU); 15 km N de Mitwaba, steppe frais de plaine, 16 Jan 1960, *P.Duvigneaud 5082L* (BRLU); Kundelungu, près du gîte Rack, 5 Feb 1969, *S.Lisowski, F.Malaisse, J.-J.Symoens 1058* (BR) & *1058a* (POZG); Kundelungu, près du gîte Rack, 5 Feb 1969, *S.Lisowski, F.Malaisse, J.-J.Symoens 1247* (BR) & *1270* (POZG); same locality, steppe arbustive, 6 Feb 1969, *S.Lisowski, F.Malaisse, J.-J.Symoens 1072* (POZG); Kundelungu, au bord de la rivière Kalembe, steppe à suffrutex, 1550 m elev., 12 Jan 1971, *S.Lisowski, F.Malaisse, J.-J.Symoens 12857* (POZG).

##### Note.

*C.duvigneaudii* is very closely related to and arguably only a variety of *C.foliatus*, differing in the ternate, narrower leaves, blade base cuneate, not clasping, apex acute, blade length/width ratio 6–10 (vs. 3–5 in *C.foliatus*). It is also related to the Angolan *C.strictipes*, differing in the ternate leaves, much more pubescent inflorescence and shorter pedicels. See note under *C.foliatus*.

#### ﻿ Coleus efoliatus

Taxon classificationPlantaeLamialesLamiaceae

De Wild., Contr. Fl. Katanga: 173. & Ann. Soc. Sci. Bruxelles 41(2): 47. 1921.

78BECD18-8821-53F9-B38C-A68C2F2F73EE

≡ Plectranthusefoliatus (De Wild.) A.J.Paton, Fl. Trop. E. Afr., Lamiac.: 289. 2009. Type: DR. Congo, Welgelegen, 6 Jun 1912, *J.Bequaert 486* (lectotype BR [BR0000006261856], designated by Paton et al. [2009]).
= Plectranthusleviculus N.E.Br., Bull. Misc. Inform. Kew 1921: 296. 1921. Type: DR. Congo, Elisabethville [Lubumbashi], Jun 1920, *F.A.Rogers 26211* (holotype K [K000431879]).
= Coleuskassneri (T.C.E.Fr.) Robyns & Lebrun, Ann. Soc. Sci. Bruxelles, Sér. B 49: 106. 1929. Type: DR. Congo, Mt Morumbe, between rocks, 12 May 1908, *T.Kassner 2951* (holotype B destroyed; isotype BM, BR [BR0000006262532], E, K, P).

##### Description.

Paton et al. (2009: 289); Paton et al. (2013: 237), as *Plectranthusefoliatus* (De Wild.) A.J.Paton.

##### Distribution.

Rwanda to S Tropical Africa.

##### Habitat and ecology.

*Brachystegia* miombo woodland, dry evergreen forest, steppic savannah, often on rocky outcrops or gravelly soil, occasionally on copper-rich soil; 1250–1400 m elev.

##### Additional specimens.

DR. Congo, ***Haut-Katanga***, Welgelegen, 6 Jun 1912, *J.Bequaert 486* (BR); Tantara, 7 Aug 1956, *P.Duvigneaud & J.Timperman 2235Col* (BRLU); Katende, 50 km S Dilolo, 1957, *P.Duvigneaud 2458Col* (BRLU); Elisabethville [Lubumbashi], May 1912, *H.Homblé 290* (BR); Lubumbashi, Katuba, 24 May 1927, *P.Quarré 450* (BR).

Rwanda, Akagera, Lac Ihema, 15 Aug 1974, *P.Van der Veken 10806* (BR).

#### ﻿ Coleus elliotii

Taxon classificationPlantaeLamialesLamiaceae

(S.Moore) A.J.Paton, Phytokeys 129: 43. 2019.

DC4502DF-EC27-539F-A523-4C73F09DB482

≡ Pycnostachyselliotii S.Moore, J. Linn. Soc., Bot. 38: 275. 1908. Type: Uganda, Ruwenzori E, 10 Feb 1906, *A.F.R.Wollaston s.n.* (holotype BM [BM000910111]).
= Pycnostachyscinerascens Robyns & Lebrun, Rev. Zool. Bot. Africaines 16: 352. 1928. Type: DR. Congo, Ruwenzori, vallée Lamia, 14 May 1914, *J.Bequaert 4287* (holotype BR [BR0000008910073]; isotype K fragment).
= Pycnostachysbutaguensis De Wild., Pl. Bequaert. 4: 389. 1928. Type: DR. Congo, Ruwenzori, Vallée Butagu, 15 Apr 1914, *J.Bequaert 3715* (holotype BR [BR0000008909749], isotype K fragment).
= Pycnostachysbequaertii De Wild., Pl. Bequaert. 4: 393. 1928., nom. illeg., non Pycnostachysbequaertii De Wild., Contr. Fl. Katanga: 171. 1921.

##### Description.

Paton et al. (2009: 406), as *Pycnostachyselliotii* S.Moore.

##### Distribution.

DR. Congo to Uganda (Ruwenzori Mts.).

##### Habitat and ecology.

*Erica* shrubland; 2000–3170 m elev.

##### Additional specimens.

DR. Congo, ***Lacs Edouard et Kivu***, NW du Ruwenzori, E de Kalasabango, 13 Sep 1952, *H.Frédéricq in G.F.de Witte 8053* (BR, WAG); Piste Kalonge-Mahungu, 12 May 1953, *H.Frédéricq in G.F.de Witte 8990* (BR); Mont Muhi, 28 Jul 1955, *U.Kinet 63* (BR); Ruwenzori, crête de la Mososa, 4 Aug 1948, *W.Robyns 3321* (BR).

#### ﻿ Coleus eminii

Taxon classificationPlantaeLamialesLamiaceae

(Gürke) A.J.Paton, Phytokeys 129: 43. 2019.

27DD84E9-AA06-5B97-8086-1F20E0A3AB1C

≡ Pycnostachyseminii Gürke, Bot. Jahrb. Syst. 22: 145. 1895. Type: Tanzania, west of Lake Victoria, Kanessa, 14 Nov 1890, *F.Stuhlmann 943* (lectotype B designated by Bruce (1940) destroyed; isolectotype K [K000405740] fragment).
= Pycnostachysrotundatodentata De Wild., Pl. Bequaert. 4: 391. 1928. Type: DR. CONGO, Ruwenzori, Kisuki, 6 Jun 1914, *J.Bequaert 4701* (lectotype: BR, designated by Bramley in Paton et al. (2009) [BR0000008909756], [BR0000008910080]).

##### Description.

Paton et al. (2009: 407), as *Pycnostachyseminii* Gürke.

##### Distribution.

Cameroon to Ethiopia and NW Tanzania.

##### Habitat and ecology.

Savannah; 1100–2600 m elev.

##### Additional specimens.

DR. Congo, ***Lacs Edouard et Kivu***, Kisuki, pied du Ruwenzori, 6 Jun 1914, *J.Bequaert 4701* (BR, K fragment); Rutshuru, 19 Oct 1914, *J.Bequaert 6052* (BR); Bwito, Kikuku, 1 Jun 1954, *A.Deru 264* (BR); Dorsale du Mont Bikingi, 16 Jun 1949, *J.de Wilde 284* (BR); Luofu, 10 Dec 1934, *G.F.de Witte 2186* (DE); Entre Beni et Lubero, Oct 1931, *J.Lebrun 4275* (BR); Rutshuru, Mont Katale, Dec 1937, *J.Lebrun 9167* (BR, LWI, US).

Burundi, Piste de la faille des Allemands, 20 May 1988, *J.Saintenoy 158* (BR).

#### ﻿ Coleus engleri

Taxon classificationPlantaeLamialesLamiaceae

(Briq.) A.J.Paton, Phytokeys 129: 44. 2019.

BF95444D-A81D-5BAA-834F-FACED366E754

≡ Anisochilusengleri Briq., Bot. Jahrb. Syst. 19: 190. 1894. Type: DR. Congo, between Nyangwe and Kimbundo, 15 Jun 1882. *P.Pogge 1019* (lectotype K [K000405621], designated by Paton et al. [2009]).
= Plectranthusafricanus (Baker) A.J.Paton, Fl. Trop. E. Afr., Lamiac.: 308. 2009. Type: Sierra Leone, Freetown, Jan 1899, *G.F.Scott Elliot 5033* (holotype K [K000405622]).

##### Description.

Paton et al. (2009: 308), Paton et al. (2013: 251), as *Plectranthusafricanus* (Baker) A.J.Paton.

##### Distribution.

W Tropical Africa to Uganda and NE Angola.

##### Habitat and ecology.

Swampy savannah, marshland, edge of riparian forest; 300–1200 m elev.

##### Additional specimens.

DR. Congo, ***Bas-Congo***, Entre Ngoma et Kikwansa, 5 May 1959, *L.Pauwels 2779* (BR); Kinanga, 6 Jul 1925, *W.Robyns 166* (BR); Dolo, Jun 1899, *R.Schlechter 12469* (BR, K); ***Kasaï***, Toni-Feshi, Kwango, 24 Jun 1955, *R.Devred 2108* (BR); Entre Lufuna et Mbombi, 6 Aug 1944, *R.Germain 2687* (BR); Territ. Dibaya, route Tshimbulu-Hemptinne, 6 Jun 1957, *L.Liben 3101* (BR); Rivière Tudi, route Kinzambi, 8 Aug 1991, *B.Masens 632* (BR, WAG, K); ***Bas-Katanga***, Gandajika, rivière Katamba, 2 Jul 1952, *R.Germain 7939* (BR); Kiabukwa, 9 Jul 1946, *G.Kevers 21* (BR); Entre Nyangwe et Malela, Aug 1932, *J.Lebrun 5940* (BR); Région sud de Kanda Kanda, rive gauche du Lubilash, 28 May 1934, *F.Luxen* 346 (BR); Kaniama, 22 Apr 1947, *W.Mullenders 298* (BR); ***Forestier Central***: Lac Léopold II [Lac Mai Ndombe], Dec 1932, *J.Lebrun 6687* (BR); ***Ubangi-Uele***: Entre Libenge et Gemena, Dec 1930, *J.Lebrun 1765* (BR); Faradje, Aug 1931, *J.Lebrun 3512* (BR); ***Lac Albert***: Kilo, 1921, *J.Claessens 1307* (BR); ***Lacs Edouard et Kivu***, Luamisole, 13 Feb 1939, *F.L.Hendrickx 151* (BR); Mwenga, Mudubwe, 21 May 1959, *A.Léonard 4289* (BR).

#### ﻿ Coleus erici-rosenii

Taxon classificationPlantaeLamialesLamiaceae

(R.E.Fr.) A.J.Paton, Phytokeys 129: 44. 2019.

297B0365-F44C-5476-9FFF-B7302C064BF1

≡ Pycnostachyserici-rosenii R.E.Fr., Wiss. Erg. Schwed. Rhod.-Kongo Exped. 1: 281. 1916. Type: DR. Congo, Niragongo, 2000 m elev., 21 Dec 1911. *R.E.Fries 1588* (holotype UPS [V-039931], isotype K [K000405955], MO).
= Pycnostachysalbidoviolacea De Wild., Pl. Bequaert. 4: 400. 1928. Type: DR. Congo, Kivu, Mukule, 26 Sep 1914, *J.Bequaert 5889* (lectotype BR [BR0000008910011], designated by Paton et al. [2009], isolectotype MO).
= Pycnostachysrobynsii De Wild., Pl. Bequaert. 4: 398. 1928. Type: BURUNDI, Busiga, 28 May 1926, *W.Robyns 2356* (holotype BR [BR0000008910356], [BR0000008909695]; isotype K, MO, P).

##### Description.

Paton et al. (2009: 408), as *Pycnostachyserici-rosenii* R.E.Fr.

##### Distribution.

E DR. Congo to Uganda.

##### Habitat and ecology.

Marshland, steppe, savannah, wooded savannah, sclerophyllous mountain forest; 1600–2420 m elev.

##### Additional specimens.

DR. Congo, ***Lac Albert***, Haut-Ituri, Kwandruma, 16 Jul 1937, *J.Ghesquière 4731* (BR); ***Lacs Edouard et Kivu***, Boswenda, 23 Oct 1914, *J.Bequaert 6087* (BR, K fragment, MO); Kabare, Lwiro, 17 Aug 1953, *A.Christiaensen 4*0 (BR, LSHI); Chaîne des Mitumba, Hangi-Kipesa, 19 Jan 1956, *J.de Wilde 624* (BR); Bitashimua, 1 Aug 1934, *G.F.de Witte 1725* (BR); Kibumba, route Rutshuru-Goma, 12 Dec 1944, *R.Germain 2993* (BR, U); Kabare, Mulungu, 6 Nov 1958, *A.Léonard 1896* (BR, K, WAG); Kalehe, Mont Kahuzi, 6 Jun 1970, *J.Ntakiyimana 78* (BR).

Rwanda, Buturo, Ruhengeri, Jan 1933, *A.Becquet 362* (BR, MO); Forêt de Nyungwe, env. Gisakura, 12 Jun 1971, *G.Bouxin 993* (BR, WAG); Kabare, colline à l’W de Tshibati, 30 Dec 1969, *Ern 15* (BR, LWI, MO); Nyarutembe, Maraba, 2 Jul 1971, *M.Radoux 19* (BR); Rangiro, Kirambo, 6 May 1980, *B.Runyinya 998* (BR); Mukura, Mont Huye, 17 Jul 1974, *G.Troupin 15122* (BR).

Burundi, Muramvya, 9 Sep 1991, *J.De Laet H82* (BR); Kisozi, 15 Jul 1935, *J.B.Lejeune 314* (BR); Bubanza, 12 Jun 1981, *M.Reekmans 10653* (BR, WAG); Karuzi, 3 Apr 1958, *D.van der Ben 2023* (BR).

#### ﻿ Coleus esculentus

Taxon classificationPlantaeLamialesLamiaceae

(N.E.Br.) G.Taylor, J. Bot. 69 (suppl. 2): 158. 1931.

16759223-648F-5A04-8685-EB7750C189B8

≡ Plectranthusesculentus N.E.Br., Bull. Misc. Inform. Kew 1894: 12. 1894. Type: cultivated at K from material sent by J. Medley Wood from KwaZulu-Natal, 1893 (lectotype K [K000975993], designated by Codd (1975); isolectotype BOL).

##### Note.

Our treatment of *C.esculentus* departs from recent floras, which recognised two species (*C.esculentus* and *C.densus*). Variation in DR. Congo is complex, comprising morphs difficult to accommodate into such a scheme. We recognise one species comprising four varieties, including a new one. Evidence from molecular markers is needed to test the validity of this treatment.

### ﻿﻿Key to the varieties of *Coleusesculentus*

**Table:** 

1	Shoot with a pair of opposite branches at the 2–5 upper nodes, forming a panicle; thyrses borne distally on these branches; bracts broadly elliptic, ca. 5 × 3 mm, 5-veined	**C.esculentusvar.kolweziensis**
–	Shoot unbranched at the upper nodes; thyrses sessile on nodes of the main stem; bracts narrowly obovate or ovate-triangular, ca. 1–3 × 1 mm, mostly 1–3 veined	**2**
2	Rachis of the thyrses 8–20 cm long; verticils spaced 2–10 mm; pedicel (2–)3–19 mm long; upper lobe of calyx obovate-elliptic, very different in shape from the other lobes	**C.esculentusvar.esculentus**
–	Rachis of thyrses 0–5 cm long; verticils spaced 1–2 mm; pedicel 0–2 mm long; upper lobe of calyx triangular, not much different in shape from the other lobes	**3**
3	Rachis of thyrses obsolete; thyrses mostly condensed together near the top of the stem into a more or less spiciform inflorescence; upper lobe of calyx 2–3 mm long, equalling the tube	**C.esculentusvar.primulinus**
–	Rachis of thyrses 1–5 cm long; thyrses widely spaced along the stem; upper lobe of calyx 1.5–2 mm long, shorter than the tube	**C.esculentusvar.densus**

#### ﻿ Coleus esculentus var. esculentus

Taxon classificationPlantaeLamialesLamiaceae

CA16B82E-3FBA-532C-BDB3-CEFD42AB666E

##### Description.

Paton et al. (2009: 291), Paton et al. (2013: 240), as *Plectranthusesculentus* N.E.Br.

##### Distribution.

Tropical and South Africa.

##### Habitat and ecology.

Degraded miombo woodland, fallow fields, savannah, 300–2285 m elev.

##### Additional specimens.

DR. Congo, ***Bas-Congo***, Kinwanda, près de Tumba Mani, 23 Aug 1902, *A.Cabra & F.L.Michel 38* (BR); Kisantu, Feb 1913, *J.Gillet s.n.* (BR); Sundi Lutete, 10 Aug 1967, *I.Persson 124* (BR); ***Kasaï***, Kwango, Kahemba, 24 Jul 1955, *R.Devred 2333* (BR); Env. Luluabourg [Kananga], 1910, *A.Sparrano 27* (BR); ***Lacs Edouard et Kivu***, Kaziba, 26 Sep 1952, *J.F.Laurent 574* (BR); ***Haut-Katanga***, Marungu, Kalewe, Apr 1944, *L.Dubois 1200* (BR); 30 km W de Mutshatsha, 28 Aug 1956, *P.Duvigneaud & J.Timperman 2530Co1* (BRLU); Marungu, Kasiki, Nov 1945, *P.Quarré 7363* (BR); Entre Masombwe et Lusinga, 21 Sep 1948, *W.Robyns 3643* (BR); Lubumbashi, 1937, *Salésiens 466* (BR, WAG). Burundi, Gitega, Bufundu, *A.Becquet 766* (BR); Kumuyange, 26 Sep 1971, *J.Lewalle 6132* (BR); Ruvironza, 15 Jul 1958, *G.Michel 5540* (BR); Mosso, Ruyigi, abords de Kinyinya, 13 Aug 1951, *G.Michel & J.Reed 85* (BR).

##### Notes.

1. Occasionally cultivated for its edible tubers; in Marungu known as “mizumbu” and, in Burundi, as “impombo” (kinyarwanda) or “inumbu” (kirundi).

2. Var. esculentus is quite variable in the length of the pedicel and the internodes of the thyrse; some specimens are more or less intermediate between var. esculentus and var. densus (e.g. *P.Quarré 7085* (BR), *J.Rammeloo 4690* [BR]).

#### ﻿ Coleus esculentus var. densus

Taxon classificationPlantaeLamialesLamiaceae

(N.E.Br.) Meerts & A.J.Paton, comb. et stat. nov.

F171D4DB-6E3B-55D4-8BAD-BC0AB3933BC4

urn:lsid:ipni.org:names:77347690-1

Plectranthus densus N.E.Brown in Bull. Misc. Inform., Kew 1894(85): 12. 1894. Type: Tanzania, N of Lake Malawi (Nyassa), Oct 1880, *Thomson s.n*. (holotype K [K000431965]).(Basionym)
≡ Coleusdensus (N.E.Br.) A.J.Paton, Phytokeys 129: 39. 2019.
= Plectranthusdekindtianus De Wild. Ann. Mus. Congo Belge, Bot., sér. 4, 2: 135. 1913. Type: DR. Congo, Bugege (?), Sep 1911. *A.Hock s.n*. (holotype BR [BR0000009824928]), syn. nov.

##### Description.

Paton et al. (2009: 292), Paton et al. (2013: 241), as *Plectranthusdensus* N.E.Br., restricted to specimens with inflorescence lax.

##### Distribution.

SW Tanzania to S Tropical Africa.

##### Habitat and ecology.

Dry woodlands, savannah, 1300–2000 m elev.

##### Additional specimens.

DR. Congo, ***Kasaï***, Kahemba, route vers l’Angola, 31 May 1948, *P.Duvigneaud 955* (BRLU); ***Haut-Katanga***, Kambove, 17 Aug 1979, *H.Breyne 3735* (BR); Lubumbashi, Kasapa, 15 Sep 1972, *J.Bulaimu 533* (BR); Upemba, piste vers Mitwaba, 12 Apr 1947, *G.F.de Witte 2488* (BR); 10 km W of Mindingi, 21 Jul 1956, *P.Duvigneaud & J.Timperman 2067* (BRLU); Upemba, entre Masombwe et Lusinga, 21 Sep 1948, *J.Lebrun 3644* (BR); Luiswishi, 2 Oct 1974, *F.Malaisse 7965* (BR); Katanga, s.l., 21 Sep 58, *J.Plancke 119/1734* (BRLU); Upemba, 25 Aug 1949, *L.van Meel* in *G.F.de Witte 7555* (BR).

Burundi. Bururi, 21 Sep 1974, *J.Rammeloo 4690* (BR).

##### Note.

*Plectranthusdekindtianus* De Wild. was synonymised with *Plectranthustetragonus* Gürke by Paton et al. (2009); however, the type specimen lacks bristles on the stem and is better placed here

#### ﻿ Coleus esculentus var. primulinus

Taxon classificationPlantaeLamialesLamiaceae

(Baker), comb. et stat. nov.

45603FF8-B8BD-548F-B6A7-DBDF040E2E09

urn:lsid:ipni.org:names:77347691-1

Plectranthus primulinu s Baker in Bull. Misc. Inform., Kew 107: 292. 1895. Type: Zambia, Mwero Plateau, 1894, *A.Carson 36* (holotype K [K000430746]).(Basionym)

##### Description.

Closely related to var. densus, differing in the much shorter thyrses, with rachis obsolete, mostly grouped into a spiciform inflorescence in the upper part of the stem; calyx lobes 2–3 mm long, equalling the tube.

##### Distribution.

Angola, Zambia, DR. Congo, W Tanzania.

##### Habitat and ecology.

Savannah, wooded savannah, in DR. Congo often on Cu/Co or Mn rich soil, 1170–1830 m elev.

##### Additional specimens.

DR. Congo, ***Haut-Katanga***, Upemba, Piste vers Mitwaba, 12 Apr 1947, *G.F.de Witte 2488* (BR); Kasompi Est, Jul 1956, *P.Duvigneaud & J.Timperman 2051* (BRLU); Kabwelunono copper hill, Jun 1997, *F.Malaisse, E.Kisimba, Y.Muzinga 134* (BR); Kwatebala copper hill, May 2007, *I.Parmentier 4347* (BR; WAG); Upemba, tête de source de la Katuba, Aug 1934, *P.Quarré 4213* (BR); Kipopo, 20 May 1982, *M.Schaijes 1397* (BR); Chabara, 11 Apr 1990, *Tropmetex 224* (BR, MO).

##### Notes.

1. Previous treatments (Paton et al. 2009, 2013) synonymised *Plectranthusprimulinus* with *Coleusdensus.* However, in DR. Congo, the two morphs can be readily recognised even though intermediates occur; therefore, we propose to recognise them at varietal rank. They occur in different habitats, var. primulinus being particularly frequent in steppic savannah on metal-rich soil in Haut-Katanga, where var. densus is rarely observed.

2. Specimens collected in the rainy season consist of leafy shoots without flowers, with leaves steadily decreasing in size upwards, the upper ones bract-like and often forming an apical coma; specimens collected in the dry season are mostly leafless and bear inflorescences in the upper part of the stem. Some specimens have particularly large leaves (up to 20 × 8 cm) with velvety tomentose indumentum (e.g. *G.F.de Witte 2488*).

#### ﻿ Coleus esculentus var. kolweziensis

Taxon classificationPlantaeLamialesLamiaceae

Meerts & A.J.Paton var. nov.

0AFC2799-8339-54CD-A12E-FA64A538A1D5

urn:lsid:ipni.org:names:77347692-1

Fig. 4A–C

##### Type.

DR. Congo, Haut-Katanga, Piste Nzilo-Kyamasumba, 10°30'29"S, 25°26'12"E, 1435 m elev., 1 Mar 1987, *M.Schaijes 3354* (BR [BR0000021718298], photos).

##### Diagnosis.

Differs from the type in the following combination of traits: thyrses borne on pairs of ascending branches, at the 2–5 upper nodes of the stem, forming a panicle; bracts broadly ovate-elliptic, ca. 5 × 3 mm, 5-veined; rhizome creeping; stem leafy at flowering; leaves narrowly obovate, < 15 mm wide, pubescent on veins beneath, very shortly pubescent above.

##### Etymology.

All collections of this variety originate from the region of Kolwezi in western Upper Katanga.

##### Distribution.

Endemic to SE DR. Congo (Haut-Katanga).

##### Habitat and ecology.

Steppic savannah, dry woodland, rocky slopes; 1300–1475 m elev.

##### Additional specimens.

DR. Congo, ***Haut-Katanga***, Env. de Sakabinda, sources de la Kengere, 9 Apr 1971, *S.Lisowski* 23304 (POZG); Env. Kolwezi, près du village Muilu, 8 Apr 1971, *S.Lisowski* 23703 (POZG); without locality, 1937, *Salésiens 1264* (BR); 19 km SSE of Kolwezi, 13 Mar 1983, *M.Schaijes 1873* (BR); Piste Kolwezi-Musokantanda (Plateau de la Manika), 10°48'42"S, 25°15'59"E, 19 Feb 1989, *M.Schaijes 4305* (BR);

**Figure 4.**
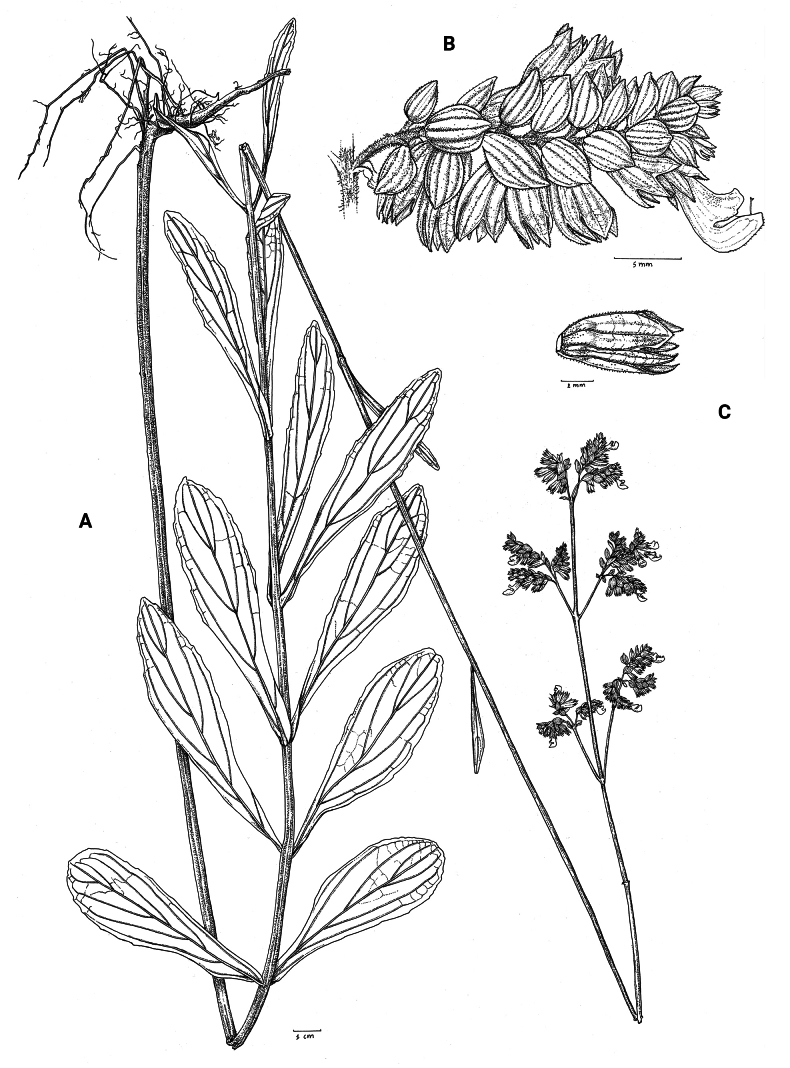
Coleusesculentusvar.kolweziensis Meerts & A.J.Paton **A** habit **B** detail of inflorescence and bracts **C** detail of calyx **A***M.Schaijes 3354***B, C***Breyne 1197*. Drawn by Hilde Orye. Scale bars: 1 cm (**A**); 5 mm (**B**); 2 mm (**C**).

##### Note.

This new variety has a restricted distribution range in the region of Kolwezi.

#### ﻿ Coleus foliatus

Taxon classificationPlantaeLamialesLamiaceae

(A.J.Paton) A.J.Paton, Phytokeys 129: 46. 2019.

FFD2F625-AD9A-504A-B030-0285B338EB01

≡ Plectranthusfoliatus A.J.Paton, Fl. Trop. E. Afr., Lamiac.: 284. 2009. Type: Tanzania, Sumbawanga District, Tatanda, Mbaa Hill, 25 Apr 1997, *S.Bidgood et al. 3459* (holotype K [K000194843], [K000194844]; isotype C, DSM, K, NHT).

##### Description.

Paton et al. (2009: 284), Paton et al. (2013: 234), as *Plectranthusfoliatus* A.J.Paton.

##### Distribution.

SW Tanzania, N Zambia, Burundi, SE DR. Congo.

##### Habitat and ecology.

Steppic savannah, scrub, mostly on highlands, often on shallow rocky soil, occasionally on copper-rich soil; 1500–2000 m elev.

##### Additional specimens.

DR. Congo, ***Haut-Katanga***, Marungu, Kasiki, 20 Jun 1931, *G.F.de Witte 470* (BR); Tenke, *P.Duvigneaud & J.Timperman 2624E* (BRLU); Mitwaba-hôtel, steppe, 9 Sep 1956, *P.Duvigneaud & J.Timperman 2691Co* (BRLU); Marungu, 1 km S Mont Lusale, 27 Jun 1957, *P.Duvigneaud 3738C* (BRLU); Kundelungu, 6.4 km NNW Katshupa, 17 Oct 1966, *F.Malaisse 4660* (BR, LSHI); Poste de Mitwaba, Aug 1945, *G.Mortelmans 96* (BR); Domaine de Muhila, près de Kansimba, 1400 m elev., steppe, 7 Nov 1970, *S.Lisowski 23488* (POZG).

Burundi, Route Matana-Rutovu, km 15, 26 Feb 1966, *J.Lewalle 469* (BR); Bururi, 3 Feb 1968, *J.Lewalle 2760* (BR, P); Luvironza, Bututsi, 3 Mar 1955, *G.Michel 4702* (BR).

##### Notes.

1. New species record for DR. Congo. Widespread on the Mitwaba and the Marungu Plateau (Haut-Katanga).

2. *Coleusfoliatus* is more variable in leaf shape in DR. Congo and Burundi compared to neighbouring regions; specimens with unusually narrow leaves (ca. 45 × 5 mm) are found in Burundi (e.g. *J.Lewalle 469*, *M.Reekmans 8659*); they differ from *C.duvigneaudii* in having opposite leaves, relatively short leaf blade and blunt leaf apex. In Upper Katanga, where *C.foliatus* and *C.duvigneaudii* are sympatric, more or less intermediate specimens occur (e.g. *S.Lisowski, F.Malaisse & J.-J.Symoens 1247*, *S.Lisowski, F.Malaisse & J.-J.Symoens 11295; A.Schmitz 3150*).

#### ﻿ Coleus frederici

Taxon classificationPlantaeLamialesLamiaceae

G.Taylor, J. Bot. 69 (suppl. 2): 159. 1931.

1995B4AC-CD9E-551E-B006-A79EBF373523

≡ Neomuellerawelwitschii Briq., Bot. Jahrb. Syst. 19: 180. 1894., non Coleuswelwitschii Briq.
≡ Plectranthuswelwitschii (Briq.) Codd, Fl. Pl. Africa 42: t. 1646. 1972. Type: Angola, Pungo Andongo, 14 Apr 1857, *F.Welwitsch 5544* (holotype BM; isotype C, K, LISU, MEL, PRE).

##### Description.

Short-lived perennial (annual or biennial after Hiern [1900]) herb, not aromatic (after Hiern [1900]), 0.60–1.20 m high. Stem erect, quadrangular, more or less crassulescent, puberulent, with very short retrorse hairs and sparse longer patent hairs and red sessile glands, branched. Leaves opposite, spreading, petiolate; blade ovate, 4.0–10.0 × 3.0–6.0 cm (up to 22 × 18 cm in Angola), base cordate to broadly rounded and shortly attenuate into the petiole, apex subacute, margin flat, crenate, teeth unequal in depth and breadth, with both sides subequal, the largest ones up to 5 mm deep; upper surface subglabrous, lower surface papillate on veins, rarely pubescent and with dense red sessile glands, ca. 5 secondary veins on either side; petiole 1.0–3.5 cm (up to 18 cm in Angola), pubescent as stem. Inflorescence lax, 10–21(–30) cm long, with 6–13 verticils spaced 15–20 mm, cymes on a 5–15 mm long peduncle, dichasial, with two subequal opposite divergent cincinni with 1 median flower, cincinni ca. 5–12 mm long, (3–)5-flowered, bracts ovate, ca. 3 mm long, early deciduous, pedicel 3–4 mm long, inserted eccentrically on calyx behind upper lobe. Flower: calyx ca. 2.5 mm long at anthesis, subglabrous, with red sessile glands, upper lip ovate, acute, ca. 1.4 mm long, upright, lower lobes much shorter, narrowly triangular, calyx at fruiting (in Angolan materials) more or less urceolate, contracted at throat, lobes parallel; corolla white or blue, with red sessile glands, ca. 18 mm long, tube sigmoid, with a narrow parallel-sided lower part ca. 4–5 mm long and a broader progressively dilated part ca. 5 mm long, lower lip ca. 9 mm long, cucullate, 5 mm deep, stamens fused, slightly exserted, anther 0.9 mm long; style undivided. Nutlets not observed (obovoid, slightly compressed, smooth, yellowish, in Angolan materials).

##### Distribution.

Angola, DR. Congo.

##### Habitat and ecology.

Shady places, scrub, forest fringe; 600–1100 m elev.

##### Additional specimens.

DR. Congo, ***Kasaï***, Kwango, Tambu [Tambo], près du village, 14 Apr 1953, *H.Callens 1173* (BR); ***Haut-Katanga***, Kundelungu, premier gué sur rivière Kalunda, entre Katwe et Lofoï, 19 May 1984, *A.Bodenghien 139* (BR).

##### Notes.

1. New species record for DR. Congo.

2. The materials collected in DR. Congo are at an early stage of flowering; however, the diagnostic character of calyx contracted at throat after anthesis is conspicuous in a few flowers in *H.Callens 1173*.

3. The specimen *A.Bodenghien 139* (BR), collected ca. 1000 km east of previously known localities, is unusual in having lower leaf surface pubescent on veins.

#### ﻿ Coleus globosus

Taxon classificationPlantaeLamialesLamiaceae

(Ryding) A.J.Paton, Phytokeys 129: 50. 2019.

BF6DD63F-C758-5AE2-9EB0-5E500FE8EE16

≡ Plectranthusglobosus Ryding, Bull. Jard. Bot. Natl. Belg. 66: 101. 1997. Type: DR. Congo, Haut-Katanga, Kundelungu Plateau, 20 Mar 1971, *S.Lisowski 23152* (holotype POZG [POZG-V-0100131], C photo).

##### Description.

Herb, probably perennial, 0.30–0.40 m high. Stem quadrangular, pubescent, with thin patent hairs and orange sessile glands. Leaves sessile, ascending to erect, narrowly elliptic to narrowly ovate, the lowermost ones broadly elliptic, 3.0–4.0 × 0.6–0.9 cm, apex obtuse to subacute, base narrowly rounded to subcordate at base, sparsely pubescent, with eglandular hairs on lower surface of veins and orange-red sessile glands on both surfaces, margin shallowly crenate to subentire, ± revolute, secondary veins diverging at a very narrow angle and distally subparallel to the margin. Inflorescence unbranched, of 1 or 2 widely spaced subglobose verticils; bracts broadly ovate ca. 6 × 6 mm, apex acuminate, deciduous; cymes 12–20-flowered; pedicel ca. 1.5 mm long; flower: fruiting calyx only slightly zygomorphic, glandular, pubescent, lobes subequal, ovate-triangular, apex subacute to obtuse, upper lobe slightly decurrent; corolla 12–14 mm long, tube 7–8 mm long, upper lip narrow, 4-lobed, with narrow lateral lobes, lower lip cucullate, 4–5 mm long, stamens connate over ca. 0.5 mm, anthers ca. 0.5 mm diam., with orange sessile glands. Nutlets ± 1.3 × 1.1 mm, dark brown, smooth, glossy.

##### Distribution.

Endemic of SE DR. Congo (Haut-Katanga, Kundelungu Plateau).

##### Habitat and ecology.

Steppic savannah on highlands, on moist soil, riverbanks, often near ponds; 1500–1700 m elev.

##### Additional specimens.

DR. Congo, ***Haut-Katanga***, Kundelungu, about 5 km NNE of the western source of Lutshipuka, 19 Feb 1969, *S.Lisowski, F.Malaisse & J.-J.Symoens 2669* (POZG); about 6 km WNW of the western source of Lutshipuka, 25 Mar 1969, S.*Lisowski, F.Malaisse & J.-J.Symoens 3793* (POZG); Mont Kabwe, 28 Mar 1971, *S.Lisowski, F.Malaisse & J.-J.Symoens 23610* (POZG).

#### ﻿ Coleus goetzenii

Taxon classificationPlantaeLamialesLamiaceae

(Gürke) A.J.Paton, Phytokeys 129: 50. 2019.

B40F7C4D-39F3-564D-8CB6-D1DB753C2FB8

≡ Pycnostachysgoetzenii Gürke in Götzen, Durch Afr., reimpr.: 8. 1896. Type: Rwanda, Sabinyo, flanc sud, ca. 2400 m elev, 1 Feb 1972, *P.Auquier 2369* (neotype BR [BR0000013410254] designated here; isoneotype K, LG, MO [MO100924134], WAG).
= Pycnostachysvulcanicola Lebrun & L.Touss., Bull. Jard. Bot. État Bruxelles 17: 71. 1943. Type: DR. Congo, Virunga, Volcan Karisimbi, Feb 1932. *J.Lebrun 5006* (holotype BR [BR0000008909725], [BR0000008910059]; isotype K, MO).

##### Description.

Paton et al. (2009: 409), as *Pycnostachysgoetzenii* Gürke.

##### Distribution.

EC Tropical Africa (Virunga Mts.).

##### Habitat and ecology.

Mountain forest with bamboo, *Hypericum*, and *Hagenia*; 2300–2800 m elev.

##### Additional specimens.

DR. Congo, ***Lacs Edouard et Kivu***, Tshakabindi, NE Visoke, 25 Jan 1955, *G.F.de Witte 11548* (BR, WAG); Versant E du Nyiragongo, Nov 1937, *J.Lebrun 8727* (BR); Ruwenzori, entre les gites de Mahangu et de Kalonge, 31 Dec 1977, *J.Lejoly 2570* (BR); Kabara, flanc E du Karisimbi, 21 Aug 1937, *J.Louis 5428* (BR, K, MO, P); Nyamuragira, gegen Biliba, 27 Aug 1954, *Stauffer 230* (BR, K, MO, WAG).

Rwanda, Lac Gando, Mar 1935, *G.F.de Witte 2298* (BR); Route Butare-Cyangugu, km 72, 9 May 1980, *G.Troupin 16246* (BR, K).

Burundi, entre Mabai et la rivière Kavumande, 12 Jun 1950, *H.Renier 238* (BR).

##### Note.

Neotypification of *Pycnostachysgoetzenii* Gürke. The holotype has disappeared (Rwanda, Kirunga Volcano [Virunga Mts.], north of Lake Kivu, 7500–8000 ft. elev., *G.Goetzen 98* [holotype B]); no isotype has been found. We select as the neotype *P.Auquier 2369*, matching the protologue, collected in the same region and at about the same altitude as the holotype.

#### ﻿ Coleus gracilipedicellatus

Taxon classificationPlantaeLamialesLamiaceae

(Robyns & Lebrun) A.J.Paton, Phytokeys 129: 51. 2019.

4D689229-AF28-5311-96E1-B6A201E2EBB5

≡ Holostylongracilipedicellatum Robyns & Lebrun, Ann. Soc. Sci. Bruxelles, Sér. B 49: 103. 1929. Type: DR. Congo, Katanga, Pweto to Baudouinville [Moba], between Kayabala and Lungulungu, 29 Apr 1926. *W.Robyns 2196* (holotype BR [BR0000008908674], [BR0000008908032], [BR0000008908681]; isotype BM fragment, BRLU fragment, E, K).
= Plectranthusbaumii Gürke in O. Warburg (ed.), Kunene-Sambesi Exped.: 356. 1903, non Coleusbaumii Gürke. Type: Angola, Kubango, Massaca, 19 Oct 1899. *H.Baum 283* (holotype B destroyed; isotype BM [BM000564009], E (as 238), G [G00437838], K, W [W19010009248], Z [Z-000018989]).

##### Description.

Paton et al. (2013: 233), as *Plectranthusbaumii* Gürke.

##### Distribution.

Southern DR. Congo to Botswana.

##### Habitat and ecology.

Miombo woodland, steppic savannah on rocks, 1300–1690 m elev.

##### Additional specimens.

DR. Congo, ***Haut-Katanga***, Katofio, 18 Aug 1948, *P.Duvigneaud 1378* (BRLU); Territ. Sakania, env. Kipushia, Mont Kasamwa, 29 Apr 1971, *S.Lisowski 23485 & 23495* (POZG); Fungurume, 23 Jul 2007, *B.Senterre 4638* (BR); Lubumbashi, 1937, *Salésiens 355* (BR, WAG); Lukafu, Apr 1900, *E.Verdick 455* (BR).

#### ﻿ Coleus gracillimus

Taxon classificationPlantaeLamialesLamiaceae

(T.C.E.Fr.) Robyns & Lebrun, Ann. Soc. Sci. Bruxelles, Sér. B 49: 106. 1929

B29500BD-8433-5FD9-821D-59647A07BAA8

≡ Englerastrumgracillimum T.C.E.Fr., Notizbl. Bot. Gart. Berlin-Dahlem 9: 69. 1924. Type: DR. Congo, Mt Corva, 15 May 1895, *G.Descamps s.n*. (holotype B destroyed; isotype UPS [V-712081], K fragment).
≡ Plectranthusgracillimus (T.C.E.Fr.) Hutch. & Dandy, Bull. Misc. Inform. Kew 1926: 481. 1926.

##### Description.

Paton et al. (2009: 290), Paton et al. (2013: 238), as *Plectranthusgracillimus* (T.C.E.Fr.) Hutch. & Dandy.

##### Distribution.

Widespread in Tropical Africa.

##### Habitat and ecology.

Miombo woodland, savannah, rocks, occasionally on copper-rich soil and on saline soil, 300–1575 m elev.

##### Additional specimens.

DR. Congo, ***Bas-Congo***, Kimbidi, 21 Apr 1959, *L.Pauwels 2571* (BR); Kibotuka, 2 Sep 1975, *L.Pauwels 5429* (BR, WAG); ***Kasaï***, Mayala, 20 Apr 1953, *H.Callens 1536B* (BR); Kwango-Mela, 20 Mar 1955, *R.Devred 1680* (BR); Kwango, entre Dinga et Mpandi, 5 May 1944, *R.Germain 2238* (BR); ***Bas-Katanga***, 30 km S Kamina, 4 Jul 1948, *P.Duvigneaud 1126Co1* (BRLU); Route Kayembe-Mukulu-Kamina, 9 May 1959, *S.Risopoulos 1071* (BR); N de Mabwe, 2 May 1949, *L.van Meel* in *G.F.de Witte 6279* (BR); ***Lacs Edouard et Kivu***, Ubwari Peninsula, 8 Oct 1979, *Y.&T.Ankei 79/0128* (BR); ***Haut-Katanga***, Kisenge, colline de Kapolo, 1956, *P.Duvigneaud & J.Timperman 2341* (BRLU); Upemba, Ganza, 30 May 1949, *L.van Meel* in *G.F.de Witte 6457* (BR); Tenke, colline Pumpi, 26 May 2007, *I.Parmentier & E.Kisimba 4606* (BR); Mont Mukuen, 6 Jun 1947, *A.Schmitz 698* (BR).

Burundi, Bururi, Rumonge, 5 Jun 1966, *J.Lewalle 892* (BR); Kininya Mosso, 28 Jun 1952, *G.Michel 3104* (BR).

##### Note.

New species record for Burundi.

#### ﻿ Coleus guerkei

Taxon classificationPlantaeLamialesLamiaceae

(Briq.) A.J.Paton, Phytokeys 129: 53. 2019.

5CEE0F9A-C880-5107-84B9-8910482E5800

##### Type.

Angola, Cuito (Kuito), 1 Apr 1900. *H.Baum 789* (holotype B destroyed; isotype E [E00193514], G [G00435305], HBG [HBG518375], K, M [M0104725], S [S-G-3335], W [W1901-0009329]).

##### Description.

Paton et al. (2009: 317), Paton et al. (2013: 259), as *Plectranthusguerkei* Briq.

##### Distribution.

Widespread in Tropical & South Africa.

##### Habitat and ecology.

Savannah on moist soil, marshland; 100–1800 m elev.

##### Additional specimens.

DR. Congo, ***Mayumbe***, Gimbi, 17 Jun 1948, *Laurent 713* (BR); ***Bas-Congo***, Ntadi, 21 Apr 1944, *R.Germain 2140* (BR); Kisantu, 1900, *J.Gillet 1337* (BR); Kibotuka, 2 Sep 1975, *N’Kunga 5429* (BR); ***Kasaï***, Gombe ya Tumba, 16 Apr 1953, *H.Callens 1274* (BR); Kananga, 24 Nov 1981, *S.Lisowski 66927* (BR); Kapanga, Jun 1933, *F.Overlaet 923* (BR); Panzi, 1925, *H.Vanderyst 16001* (BR); ***Bas-Katanga***, Gandajika, 12 Oct 1956, *L.Liben 1705* (BR); Katumanga, 29 Oct 1956, *L.Liben 1811* (BR); ***Ubangi-Uele***, Gangala na Bodi, 21 Nov 1942, *C.Cornet d’Elzius et al. 373* (BR); Faradje, 28 Dec 1949, *Costermans 62* (BR); Garamba, piste centrale vers km 82, 18 Feb 1952, *G.Troupin 65* (BR); ***Lac Albert***, Irumu, 9 Mar 1914, *J.Bequaert 2844* (BR); Kerekere, 19 Jan 1960, *D.Froment 649* (BR); ***Haut-Katanga***, Dilolo, riv. Mangoa, 20 Aug 1956, *P.Duvigneaud & J.Timperman 2410* (BRLU); Baudouinville [Moba], 5 May 1926, *W.Robyns 2241* (BR).

Rwanda, Agatete, 19 Nov 1953, *L.Liben 963* (BR).

Burundi, Musumba Mosso, 10 Jun 1952, *G.Michel 2674* (BR); Mosso, Ruyigi, 4 Sep 1951, *G.Michel & J.Reed 178* (BR); Route Rusengo-Cankuso, km 5, 16 May 1978, *M.Reekmans 7003* (BR, WAG).

#### ﻿ Coleus hadiensis

Taxon classificationPlantaeLamialesLamiaceae

(Forssk.) A.J.Paton, Phytokeys 129: 54. 2019.

B53D338C-7B93-5C41-9017-5EEEC156BE51

≡ Plectranthushadiensis (Forssk.) Schweinf. ex Sprenger, Wiener Ill. Gart.-Zeitung 19: 2. 1894. Type: Yemen, in montibus Hadiensis [Hadiyah], 1763, *P.Forsskål 348* (holotype C [C10002654]).
= Plectranthuszatarhendii sensu Troupin & Ayob., Fl. Rwanda 3: 340. 1985., non (Forsskal) E.A.Bruce.
= Plectranthusfragrans Lebrun & L.Touss., Bull. Jard. Bot. État Bruxelles 17: 70. 1943. Type: DR. Congo, Katanda, Sep. 1937. *J.Lebrun 7618* (holotype BR [BR0000006262556]; isotype K, P).

##### Description.

Paton et al. (2009: 300), Paton et al. (2013: 247), as *Plectranthushadiensis* (Forssk.) Sprenger.

##### Distribution.

Widespread, Egypt to South Africa, Arabian Peninsula, Maldives,

Sri Lanka.

##### Habitat and ecology.

Savannah, xerophilous scrub, woodland, rocks; 950–2150 m elev.

##### Additional specimens.

DR. Congo, ***Lacs Edouard et Kivu***, Beni, Kasindi, 10 Aug 1914, *J.Bequaert 5233* (BR); Katanda, Aug 1937, *J.Lebrun 7589* (BR); Katanda, Sep. 1937, *J.Lebrun 7618* (BR); Ruindi, 1937, *J.Lebrun 7933* (BR, YBI); Escarpement de Kabasha, 7 Dec 1934, *G.F.de Witte 2172* (BR); Lac Edouard, baie de Kabale, 24 Dec 1953, *D.van der Ben 972* (BR).

Rwanda, Ibere Rya Bigogwe, 8 Feb 1984, *F.-X.Ayobangira 1673* (BR); Bugesera, env. Karama, 12 Mar 1972, *G.Bouxin 1365* (BR); Mayaga, 27 May 1954, *L.Liben 1202* (BR); Mutara, env. Gabiro, 5 Apr 1957, *G.Troupin 3123* (BR); Akagera, Lac Ihema, 1 May 1973, *G.Troupin 15042* (BR).

##### Note.

In the Flore du Rwanda (Troupin and Ayobangira 1985), *C.hadiensis* is erroneously referred to as *Plectranthuszatarhendii*, a species absent from Central Africa.

#### ﻿ Coleus heterotrichus

Taxon classificationPlantaeLamialesLamiaceae

Briq., Bull. Soc. Roy. Bot. Belgique 40: 40. 1901.

913FD0B9-8FB3-5BD7-B2DD-0297B9F33A6A

Figs 1G–H , 2D

= Coleusclaessensii De Wild., Bol. Soc. Ibér. Ci. Nat. 19: 117. 1920. Type: DR. Congo, Mobwasa, 1910, *J.Claessens 741* (lectotype BR [BR0000006261863]; isolectotype BR [BR0000006262198], designated here), syn. nov.
≡ Calchasclaessensii (De Wild.) P.V.Heath, Calyx 5: 160. 1997.
= Coleusringoetii De Wild., Contr. Fl. Katanga: 174. & Ann. Soc. Sci. Bruxelles 41(2): 50. 1921. Type: DR. Congo, Shinsenda, Mar 1912, *A.Ringoet 546* (holotype BR [BR0000006262907], [BR0000006263232]), syn. nov.
= Coleustermetophilus De Wild., Contr. Fl. Katanga: 175. Ann. Soc. Sci. Bruxelles 41(2): 50. 1921. Type: DR. Congo, Kundelungu, swamps, 13 Mar 1908, *T.Kassner 2618* (lectotype BR [BR0000008732170], right-hand specimen only; designated here; isolectotype Z [Z-000018897]), syn. nov.
= Coleusquarrei Robyns & Lebrun, Rev. Zool. Bot. Africaines 16: 3. 1928. Type: DR. Congo, Elisabethville [Lubumbashi], ferme de Kibembe, 30 Jul 1927, *P.Quarré 608* (holotype BR [BR0000006262570]), syn. nov.
= ?C.eetveldeanus Briq., Bull. Soc. Roy. Bot. Belgique 37: 73. 1899. Type: DR. Congo, Haut-Katanga, M‘Toa, 15 May 1899, *G.Descamps s.n*. (holotype BR [BR0000008109156]).

##### Type.

DR. Congo, Bolobo, Jun 1891. *F.Demeuse 455* [“1155” in the protologue in error] (holotype BR [BR0000021454103]; isotype G [G00435192]).

##### Description.

Annual herb, occasionally scrambling, aromatic, (0.15–)0.25 –0.90(–2.00) m high; rootstock fibrose, without tubers. Stem erect, simple or branched, quadrangular, somewhat lignified in lower part in robust specimens, in the lower part with very short, papilliform (ca. 0.1 mm long) patent to recurved hairs and sessile red glands or very short glandular patent hairs and sparse longer patent hairs especially at nodes, in the inflorescence with mostly very short (ca. 0.1 mm long, papilliform), patent to slightly recurved eglandular and gland-tipped hairs, and sparse long multicellular patent hairs (ca. 1 mm long), these sometimes almost lacking. Leaves opposite, ascending to spreading, petiole 1.0–4.5(–10) cm long, pubescent like the stem, with dense very short hairs (retrorse or patent) and sparse, much longer patent hairs; blade occasionally with a purplish spot in the middle, ovate to broadly ovate-triangular, (1.0–)3.0–7.0(–12) x (1.0–)2.5–5.0(–8.5) cm, apex acute to obtuse, base broadly rounded, or truncate to subcordate and then shortly attenuate into the petiole, membranous, ca. 4–5 pairs of secondary veins, margin crenate, upper surface with sparse, appressed, antrorse hairs, lower surface very shortly pubescent on veins, (occasionally papillate over the whole surface), rarely with long patent hairs on veins, with red sessile glands. Inflorescence lax, (5–)12–32(–40) cm long, 2–6(–10) cm broad at fruiting, with (2–)7–20(–28) verticils spaced 10–25(–40) mm, bracts whitish, ovate, acuminate, cucullate, 2–7 mm long, ciliate, early deciduous (very rarely persistent at lower verticils), cyme with a 1–2 mm long peduncle, often dichasial, each cyme consisting of two subequal cincinni, diverging at right angle, elongating to 5–20(–50) mm in fruit, lax, each with 4–7(–20) flowers (i.e. cyme with 9–15(–41) flowers), spaced ca. 3 mm, pedicels 2–5(–6) mm long, with very short papilliform, eglandular and gland-tipped hairs and occasional longer multicellular hairs and red sessile glands, pedicel curving at tip, inserted eccentrically in front of calyx upper lobe. Flower: calyx 1.5 mm long at anthesis, 3–5.5 mm in fruit, shortly pubescent and with red sessile glands, tube campanulate ca. 2 mm long, throat truncate, upper lobe broadly ovate to almost round, 1.5–2.5 × 1.5–2.5 mm, apiculate, very shortly decurrent, curving upwards, lateral lobes of lower lip oblong-rectangular, truncate, lower lobes fused into an oblong linear lower lip ca. 2.5–3 mm long with two acute teeth. Corolla blue, with red sessile glands, 8–13 mm long, tube strongly sigmoid 4 mm long, widening near throat, upper lip 1–2 mm long, lower lip 4–7 mm long, 2.5 mm deep, thinly puberulent, stamens fused, tube sigmoid; anther 0.5 mm.

##### Distribution.

Sierra Leone, Ivory Coast, Nigeria, Central African Republic, Ethiopia, Sudan, South Sudan, Tanzania, Mozambique, Malawi, Zambia, Angola.

##### Habitat and ecology.

Rainforest, savannah, dry woodland, dry evergreen forest, fallow fields, ruderal, disturbed ground, rock outcrops, mostly in shady places, occasionally on copper-rich or saline soil; 300–1750 m elev.

##### Additional specimens.

DR. Congo, ***Bas-Congo***, Kimbuba, 27 Oct 1958, *Pauwels 386* (BR); Kimbidi, 21 Apr 59, *L.Pauwels 2536* (BR); ***Kasai***, Lisha, 28 Apr 1888, *F.Hens C6* (Z, marked as “holotype” in error); Kapanga, 1934, *F.Overlaet 1202* (BR); Thielen Saint-Jacques, *H.Vanderyst 21575* (BR); ***Bas-Katanga***, Tshikamba près Mutombo-Mukulu, Jun 1931, *P.Quarré 2512* (BR); Kamina, 11 Mar 1959, *S.Risopoulos 871* (BR); Kiala, Dec 1954, *Thiébaud 210B* (BR); ***Forestier Central***, Eala, 28 Nov. 1943, *R.Germain 1731* (BR); Entre Bokuma et Bokatola, Sep 1933, *J.Lebrun 1313* (BR); Yangambi, île Yalututcha II, 18 Aug 1938, *J.Louis 10905* (BR, P, U, US); Route Kisangani-Lubutu, 20 Nov 1982, *L.Pauwels 6592* (BR); ***Ubangi-Uele***, Gatanga, Jan 1936, *A.M.De Graer 484* (BR); Garamba, 13 Oct 1951, *H.De Saeger 1438* (BR); Entre Businga et Banzyville [Mobayi-Mbongo], Jan 1930, *J.Lebrun 2009* (BR); ***Lac Albert***, Kurukwata, 4 Nov 1957, *P.Gérard 3586* (BR); ***Lacs Edouard et Kivu***, Ruzizi, Route Uwira-Mbaraka, km 150, May 1950, *Germain 7009* (BR); Ironga, Mutongo, *Gutzwiller 2734* (BR); Lac Mokoto, 21 Jul 1953, *D.van der Ben 634* (BR); ***Haut-Katanga***, Mwashya, May 1939, *H.J.Bredo 2757* (BR); Upemba, riv. Kenia, 28 Mar 1947, *G.F.de Witte 2440* (BR); Kabiashia, 19 Mar 68, *F.Malaisse 5438* (BR, LSHI); Lubumbashi, Mar 1933, *P.Quarré 3159* (BR); Pweto, chutes de la Kafisia, 18 Apr 1926, *W.Robyns 2021* (BR); Luishia, 3 Apr 1990, *Tropmetex 82* (BR, K, MO, MPN, WAG). Burundi, Lac Nyanza, *J.Lewalle 6063* (BR); Route Bugarama, km 8, 2 Jun 1976, *M.Reekmans 5184* (BR); Kabezi, 9 Apr 1978, *M.Reekmans 6892* (BR, WAG); Bururi, Rumonge, 20 Mar 1981, *M.Reekmans 9851* (BR, WAG).

Sierra Leone, Bumban National Park, 25 Sep 1967, *J.K.Morton & S.L.Cole 4920* (SL, K, GC).

Ivory Coast, Inselberg near Duékoué 6°45'N, 7°22'W, 25 Oct 1991, *S.Porembski 1000* (B, K).

Nigeria, Jos Plateau, Naraguta, 18 Oct 1957, *F.N.Hepper 1076* (BR, K). Cameroon, about 15 km. NE of Meiganga, 24 Nov 1964, *W.J.J.O. de Wilde & B.E.E. de Wilde-Duyfjes 4039* (BR).

Sudan, near summit of Gebel Lothir, 27 Nov 1930, *N.D.Simpson 7605* (K).

South Sudan, Kajiko north, 29 Oct 1982, *P.Kosper 128* (K). Ethiopia, Wellega Region, ca. 30 km S of Asosa. 9°55'N, 34°40'E, *E.I.Friis et al. 7878* (AAU, C, K).

Tanzania, Kigoma rural Distr, Gobe Stream Reserve, Kakombe Valley, 04°39'50"S, 29°37'22"E, 15 May 1999, *P.Gobbo et al. 320* (K, MO).

Mozambique, Serra Macula, Mercula, Simba Camp, 12°04.5'S, 37°38'E, 10 Jun 2003, *J.S.Golding, J. Timberlake & P.Clarke 32* (K).

Malawi, St. Kizito Seminary, Mtandere Mission, 14 miles E of main road, 25 Apr 1971, *Pawek 4668* (K, MAL).

Zambia: Choma Distr., Sinazongwe/Choma road, 11.6 km from the junction, 16°52'08"S, 27°16'23"E, 10 Mar 1997, *B.Luwiika, D.K.Harder, H.H. Schmidt, & N.B.Zimba* 608 (BR, K, MO).

Angola. Lunda Norte, 25 km S of Capaia and 55 km WSW of Lucapa, 8°33'25"S, 20°15'13"E, 1 Apr 2013 *D.G.Goyder & I.Darbyshire 7175* (K).

##### Notes.

1. *Coleusheterotrichus* has been overlooked by recent floras, being synonymised with *C.bojeri*. In the *C.bojeri* complex, it has a most distinctive combination of traits, including long cincinni and stem pubescence of short papilliform hairs and sparse long multicellular hairs; it also tends to have larger leaves with cordate base, but this trait is more variable. Some poorly-grown specimens have short cymes and can be deceptive, for example, *T.Kassner 2652* (BR, P), *J.Bequaert 3100* (BR), but indumentum is typical.

2. *C.heterotrichus* is one of the most widespread *Coleus* species in Central Africa, with a surprisingly broad ecological range, from rainforest to rocky outcrops of Katanga.

3. *C.claessensii* De Wild. is a morphotype with exceptionally long cincinni. *C.quarrei* De Wild. is a dwarf form, branching from the base.

4. *Coleuseetveldeanus* was synonymised with *Plectranthusdupuisii* (= *C.welwitschii*) by Paton et al. (2019); it probably belongs here, differing from typical *C.heterotrichus* in the shorter pedicels (1–2 mm long); however, underground parts are missing and conclusive placement is not possible.

5. Bracts are occasionally more or less persistent (e.g. *M.Micha 349*, *M.Reekmans 436*).

6. Lectotypification of *Coleusclaessensii* De Wild. *J.Claessens 741* (BR [BR0000006261863]) is selected as the lectotype because it is the most complete specimen. Remaining syntype: *A.Sapin s.n*. (syntype BR), DR. Congo, Ekuta on the Lua, 1912.

7. Lectotypification of *Coleustermetophilus* De Wild. The very brief protologue published in 1921 without a Latin diagnosis (De Wildeman 1921a) indicates “Elisabethville (Homblé)” as the type. A much more extensive protologue, with a Latin diagnosis (De Wildeman 1921b), indicated the following syntypes: *Homblé 154, Homblé 220, Ringoet 397, Ringoet* in *Homblé 534* and *Kassner 2618*. Of all these specimens, only two sheets are identified as *Coleustermetophilus* in De Wildeman’s handwriting, i.e. [BR0000008732170] and [Z-000018897]. The sheet [BR0000008732170] has two collecting labels, i.e. the original label “*Homblé 220*” and a posterior label “*Kassner 2618*”. It comprises two specimens marked “a” (right-hand) and “b” (left-hand), respectively. The label in the left-hand corner of the sheet bears the following note in De Wildeman’s handwriting: “a) *Coleustermetophilus* De Wild. n.sp.; b) *Coleushomblei* De Wild.”. The protologue of *C.termetophilus* (“pétiole atteignant 6 cm de long, limbe ovale, tronqué à la base”) corresponds only to the right-hand specimen (*T.Kassner 2618*). The other specimen (*Homblé 220*) is *C.homblei* De Wild. Another sheet in BR is also labelled “*Homblé 220*” [BR0000017710053], with three specimens also corresponding to *Coleushomblei* De Wild., clearly not matching the protologue of *C.termetophilus*. Therefore, *Homblé 220* must be excluded from the original materials of *C.termetophilus*. The right-hand specimen on sheet [BR0000008732170] corresponds to *T.Kassner 2618* and is most likely a duplicate from the gathering *Kassner 2618* in Z [Z-000018897]. In the protologue of *C.termetophilus*, De Wildeman wrote about *T.Kassner 2618*: “ce dernier échantillon provenant de l’herbier de Zürich, mis à notre disposition par le Dr. H. Schinz…” indicating that De Wildeman had indeed received a duplicate. Another sheet in BR [BR0000017710183] is also a duplicate of *T.Kassner 2618*, donated by P, apparently not seen by De Wildeman. Based on this, I designate *T.Kassner 2618* (right-hand specimen on sheet [BR0000008732170) as the lectotype of *Coleustermetophilus*; *T.Kassner 2618* [Z-000018897]; [BR0000017710183] are isolectotypes.

8. New species record for Sierra Leone, Ivory Coast, Nigeria, Central African Republic, Ethiopia, Sudan, South Sudan, Tanzania, Mozambique, Malawi, Zambia and Angola.

#### ﻿ Coleus hildei

Taxon classificationPlantaeLamialesLamiaceae

Meerts & A.J.Paton sp. nov.

C79A126B-8F2F-544D-A681-B1ED2DD0F2D2

urn:lsid:ipni.org:names:77347693-1

Fig. 5A–F

##### Type.

DR. Congo, Haut-Katanga, Parc national de l’Upemba, flancs du muleshi, stream Sense, 1400 m elev., 24 Feb 1948, *G.F.de Witte 3455* (holotype BR [BR0000017707978]; isotype K).

##### Diagnosis.

Related to *Coleusbojeri* and other species formerly referred to the genus *Solenostemon* on account of the lower calyx lobes fused into a lip, differing in the following combination of traits: rootstock bearing fusiform tubers, petiole winged over the whole length, leaf apex acute to acuminate, pedunculate cyme, longer pedicels (mostly 3–7 mm long), longer fruiting calyx (5–7 mm long).

##### Description.

Perennial herb, ca. 0.3–0.9 m high; rootstock fibrose, with a fascicle of fusiform tubers 1–3 cm long (rarely collected). Stem erect, most often simple, occasionally sparingly branched, quadrangular, puberulent, with very short adpressed retrorse hairs and red sessile glands, becoming denser and patent in the inflorescence. Leaves opposite, ascending, petiolate, occasionally with fascicules of young leaves in the axils in robust specimens; blade thin, membranous, ovate to narrowly ovate, apex acute to long acuminate (often obtuse in lowermost leaves), base rounded to cuneate, then attenuate and decurrent on the petiole, margin regularly crenate to serrate (ca. 2–3 teeth/ cm), (2.6–)6–8.5 × (0.8–)1.2–3.8 cm, 3–5 pairs of secondary veins, upper surface subglabrous to very shortly pubescent, with appressed hairs pointing to tip, lower surface very shortly pubescent on mid-vein (retrorse hairs) and reticulation, glabrous elsewhere save numerous red sessile glands (ca. 20/mm^2^); petiole 0.7–3.5(–4.5) cm long, very narrowly winged, ciliate. Inflorescence terminal, simple or with 1 or 2 pairs of basal branches, lax, (6–)10–30 cm long, 15–25 mm wide (corollas excluded) at anthesis, up to 4 cm wide in fruit, verticils 10–15(–40) mm apart, bracts membranous, cucullate, ovate-elliptic, ca. 6 b×3 mm, contracted into an acumen, caducous or occasionally persisting, shortly pubescent outside and with red sessile glands; cymes with a 1–8 mm long peduncle, ascending to spreading, the lowermost cymes dichasial, with the basal branches diverging at an open angle, 7–11(–19)-flowered, branches with mixed indumentum of papillae and short spinulose hairs, cincinni elongating to 2 cm in fruit, pedicel variable in length in a cyme, (1–)3–7(–12) mm long, decreasing in length from base to top of cyme, inserted very eccentrically opposite the upper calyx lobe. Flower: calyx ca. 2 mm long at anthesis, very shortly pubescent and with red sessile glands, fruiting calyx 5–7 mm long, tube 1.5–2 mm long, upper lobe ovate, ovate-elliptic to obovate-elliptic, ca. 3 mm long, acute to rounded, recurved, slightly decurrent, lateral lobes ca. 1.5–2 mm long, truncate to obtuse, often slightly contracted near middle, lower lobes fused into a linear lip ca. 4 mm long, markedly longer than the other lobes, straight to slightly curved upwards distally, ending in two subaristate teeth ca. 1.5–2 mm long; corolla ca. 12–13 mm long, tube strongly sigmoid, longer than calyx, widening near throat, lower lip. ca. 7 mm long, 3–4 mm deep, with red sessile glands, upper lip ca. 2–3 mm long, bilobate; staminal filaments fused, anthers subglobose, connective often with 2–3 red sessile glands. Nutlets subglobose, smooth, pale brown, densely red-speckled, ca. 1 mm.

**Figure 5.**
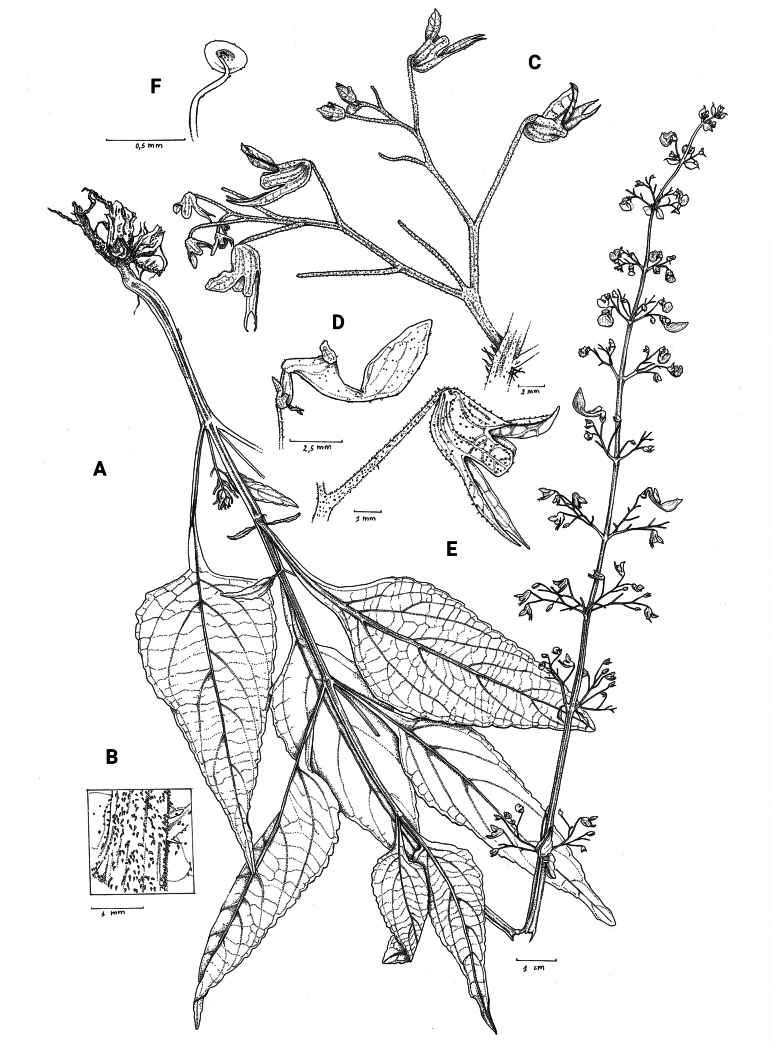
*Coleushildei* Meerts & A.J.Paton **A** habit **B** detail of pubescence of lower surface of mid-vein **C** cyme **D** flower **E** fruiting calyx and pedicel **F** stamen **A***De Troyer 56* & *G.F.de Witte 5644* (tubers) **B, C***G.F.de Witte 3455***D***G.F.de Witte 5679***E***G.F.de Witte 5689***F***G.F.de Witte 5679*. Drawn by Hilde Orye. Scale bars: 1 cm (**A**); 1 mm (**B**); 2 mm (**C**); 2.5 mm (**D**); 1 mm (**E**); 0.5 mm (**F**).

##### Etymology.

Dedicated to Hilde Orye, eminent botanical illustrator, chairwoman of the association of Belgian botanical artists, who produced all the original illustrations of this article.

##### Distribution.

Endemic of SE DR. Congo (Haut-Katanga).

##### Habitat and ecology.

Miombo woodland, scree, rocky hills; 800–1500 m elev.

##### Additional specimens.

DR. Congo, ***Haut-Katanga***, Mukulakulu, forêt claire sur sol caillouteux, 24 Mar 1953, *De Troyer 56* (BR); Parc national de l’Upemba, rive droite de la Kalule Nord, contreforts du Mont Kia, près de Biamabwa, 1090 m elev., forêt katangaise d’altitude, 28 Feb 1949, *G.F.de Witte 5644* (BR); Versant N du Mont Kia, rive droite de la Kalule Nord, forêt katangaise d’altitude, saxicole sur éboulis, vers 1090 m elev., 1 Mar 1949, *G.F.de Witte 5679*, *5689*, *5690* (BR); Kundelungu, 830 m elev., forêt claire, exp. SO, pente 45°, 29 Jan 1954, *R.Desenfans 4974* (BRLU); Fungurume, Shandiranzoro west, 21 Mar 2007, *F.Malaisse & E.Kisimba 372* (BR).

##### Notes.

1. *C.hildei* belongs to the group of species formerly referred to the genus *Solenostemon* (lower calyx lobes fused into a lip). It has affinities with the *Coleusbojeri* complex. It differs in the tuberous roots, the much taller habit, the pedunculate, dichasial cymes, the longer pedicels, the larger fruiting calyx and the winged petiole.

2. Vernacular name: tombwe (in kiluba).

#### ﻿ Coleus homblei

Taxon classificationPlantaeLamialesLamiaceae

De Wild., Contr. Fl. Katanga: 174. & Ann. Soc. Sci. Bruxelles 42(1): 49. 1921.

9A165417-EA3A-541A-9493-B44BF30DA1A2

= Plectranthussigmoideus A.J.Paton, Fl. Trop. E. Afr., Lamiac.: 333. 2009., syn. nov.
≡ Coleussigmoideus (A.J.Paton) A.J.Paton, Phytokeys 129: 97. 2019. Type: Zambia, track opposite turning to Mbala (Abercorn) Club, *H.M.Richards 4353* (holotype K [K000070564]), syn. nov.

##### Type.

DR. Congo, Elisabethville [Lubumbashi], brousse, Feb. 1912. *H.Homblé 210* (lectotype BR [BR0000006262181], designated here).

##### Description.

Short-lived perennial herb 0.20–0.40(–0.65) m, rootstock a thin rhizome rooting at nodes, tubers occasionally observed (not collected in Central Africa). Stem erect, quadrangular, with short appressed retrorse hairs (also patent hairs outside Central Africa), yellowish to green, mostly unbranched, rarely with a few erect branches. Leaves opposite, in ca. 5 or 6 pairs, the uppermost pair often much reduced, petiolate, except uppermost pair, ascending, petiole 0.2–2.0(–2.5) cm with short retrorse hairs and a few long cilia, blade ovate, ovate-elliptic to subrhombic, 1.2–4.5(–5.0) × 0.8–2.5(–3.2) cm, apex acute, base cuneate and shortly attenuate into the petiole, margin narrowly recurved, with 4–8 sharp teeth on either side (2–3 mm deep on adaxial side), upper surface pubescent, with a mixed indumentum of antrorse hyaline hairs 0.5–2 mm long and very short papilliform hairs, lower surface very shortly appressed pubescent on veins (hairs often retrorse), also with red sessile glands, 3–5 pairs of secondary veins. Inflorescence lax, 5–13(–17) cm long, with 3–9(–15) verticils spaced 5–25 mm; bracts narrowly ovate, cucullate, 3–7 mm long, acuminate, forming an apical coma, caducous; cymes ascending to spreading, sessile, with 3–7 flowers on a 0–2 mm long rachis; pedicels ca. 4–6 mm long at anthesis, spreading to slightly ascending. Flower: calyx campanulate, ca. 3 mm long at anthesis, shortly pubescent, with red sessile glands, widely open; fruiting calyx 5–6 mm long, lateral teeth obtuse, ca. 2 mm long, lower teeth fused in a ca. 4 mm long very narrow lip, with two acute tips; corolla pale blue or white, with red sessile glands, ca. 9–13 mm long, tube ca. 5 mm long, strongly sigmoid, lower lip ca. 6–7 mm long, 4 mm deep, upper lip ca. 2 mm long; staminal filaments fused, anther ca. 0.5 mm long. Nutlets brown, red-speckled, ovoid, ca. 1 mm long.

##### Distribution.

SW Tanzania to Zambia and SE DR. Congo.

##### Habitat and ecology.

Miombo woodlands, often with *Brachystegiamicrophylla*, on shallow rocky soil, termite mounds; ca. 1250–1520 m elev.

##### Additional specimens.

DR. Congo, ***Haut-Katanga***, Près de Lubumbashi, colline Kiswishi, 24 Feb 1987, *F.Billet & B.Jadin 4227* (BR, UPS); Dilolo, 1935, *de Wouters d’Oplinter 3* (BR); Lubumbashi, route de la mine de l’Etoile, km 12, 19 Apr 1957 *P.Duvigneaud 2847Co* (BRLU); Entre Welgelegen et Kasumbalesa, 29 Jan 1960, *P.Duvigneaud 5302C* (BRLU); Kasombo, 3 Feb 1960, *P.Duvigneaud 5384Col* (BRLU); 12 km NW de Lubumbashi, 12 Mar 1958, *A.Gathy 300* (BRLU); Elisabethville [Lubumbashi], Mar 1912, *H.Homblé 220* (BR) (about this specimen, see note under *C.heterotrichus*); Kasapa, 26 Feb 1966, *F.Malaisse 4012* (BR, LSHI); Ferme prince Léopold, 21 Feb 1927, *P.Quarré 1032* (BR); Keyberg, 9 km SW of Lubumbashi, 27 Feb 1948, *A.Schmitz 1392* (BR); 14 km from Lubumbashi, 17 Feb 1966, *J.-J.Symoens 12233* (BR, K, LSHI); Likasi, Panda, 25 Mar 1970, *S.Lisowski 23382* (POZG).

##### Notes.

1. *C.homblei* is superficially similar to *C.bojeri* and was synonymised with it by Paton et al. (2009, 2013). It differs from it in the perennial habit (this often difficult to observe), the more sharply serrate leaf margin, verticils with fewer flowers, longer pedicels and longer calyx at anthesis. The generally unbranched shoot is also typical with only ca. 5 pairs of leaves, the uppermost pair sessile and bracteiform. The species is widespread in miombo woodlands in the region of Lubumbashi.

2. Lectotypification of *Coleushomblei* De Wild. De Wildeman (1921b) cited two syntypes, i.e. *H.Homblé 210* (syntype BR [BR0000006262181], [BR0000006262518]), DR. Congo, Elisabethville [Lubumbashi], brousse, Feb 1912 & *H.Homblé 1259* (syntype BR [BR0000005201518], [BR0000005201846]), Plateau Biano, Tshisinka, partie boisée, Feb 1913. Both syntypes are unusual in having shoots branched from the base. Both match the protologue. *H.Homblé* 210 is more representative of the sharply serrate leaf margin and sheet [BR0000006262181] is designated as the lectotype because the label has “*Coleushomblei*” in De Wildeman’s handwriting.

3. The type materials of *Coleussigmoideus* A.J.Paton show the diagnostic traits of *Coleushomblei* De Wild., differing only in the presence of tubers, while tubers have not been collected in materials from DR. Congo.

4. See also note under C. *heterotrichus*.

#### ﻿ Coleus kaminaensis

Taxon classificationPlantaeLamialesLamiaceae

Meerts & A.J.Paton sp. nov.

33F063A9-46EA-5604-B3DB-711B2EF82743

urn:lsid:ipni.org:names:77347694-1

Fig. 6A–F

##### Type.

DR. Congo, Kamina, la Lovoi, Apr 1932, *P.Quarré 2999* (holotype BR [BR0000017712682], [BR0000017712699], [BR0000017712750], [BR0000017712767]; isotype K).

##### Diagnosis.

Related to *C.gracilipedicellatus*, on account of habit, profusely branched paniculate inflorescence, filiform pedicels jointed near apex and rachis distally zigzagging, differing in the anther not forming a pouch. It is also related to *C.bifidus*, on account of inflorescence architecture and anther structure, differing in the shallowly lobed style and the subentire leaf margin. The calyx of *C.kaminaensis* also differs by having the upper calyx lobe narrower and subulate at apex rather than clearly triangular in shape.

##### Description.

Perennial herb, 1.75–2.25 m high, with a horizontal rootstock (fide Quarré, not observed). Stem erect, thick, woody at base, rounded, slightly striate, pale brownish to purplish, lenticellate, subglabrous to thinly appressed puberulous, with short appressed antrorse hairs in the inflorescence, branching in upper half. Leaves opposite, ascending, subsessile, blade elliptic to ovate-elliptic, 4–8.5 × 1–2 cm, base cuneate to shortly attenuate, apex acute, margin shallowly and remotely crenate, to subentire, recurved, 4–5 pairs of secondary veins, shortly appressed pubescent on veins beneath, with red sessile glands, puberulous on upper surface; petiole 0–0.1 cm. Inflorescence paniculiform, lax, ca. 30 × 20 cm, much branched, flowers solitary in the axil of a small bract, more or less spirally or distichously arranged, occasionally 2 pedicels opposite at a node; inflorescence axis slender and slightly zigzagging at apex, puberulous, bracts narrowly ovate to linear, acute, ca. 1–1.5 mm long, caducous; pedicels 5–25 mm long, filiform, faintly jointed near tip, glabrous below joint, shortly pubescent as calyx above; calyx ca. 5 mm long at anthesis, shortly appressed pubescent and with orange-red sessile glands, tube campanulate ca. 2.5 mm long, faintly 10-veined, lobes subulate, ca. 1.5–2 mm long, separated by broad truncate sinuses, with a short basal membrane; mature calyx not observed. Corolla blue (fide Quarré), ca. 9–11 mm long, tube almost straight, ca. 4–6 mm long, lower lip ca. 5 mm long, distally puberulous, curving upwards and closing throat, upper lip ca. 2 mm, upwardly pointing; stamens not exserted, filaments fused, anthers orbicular, not forming a pouch; style very shallowly lobed. Nutlets not observed.

**Figure 6.**
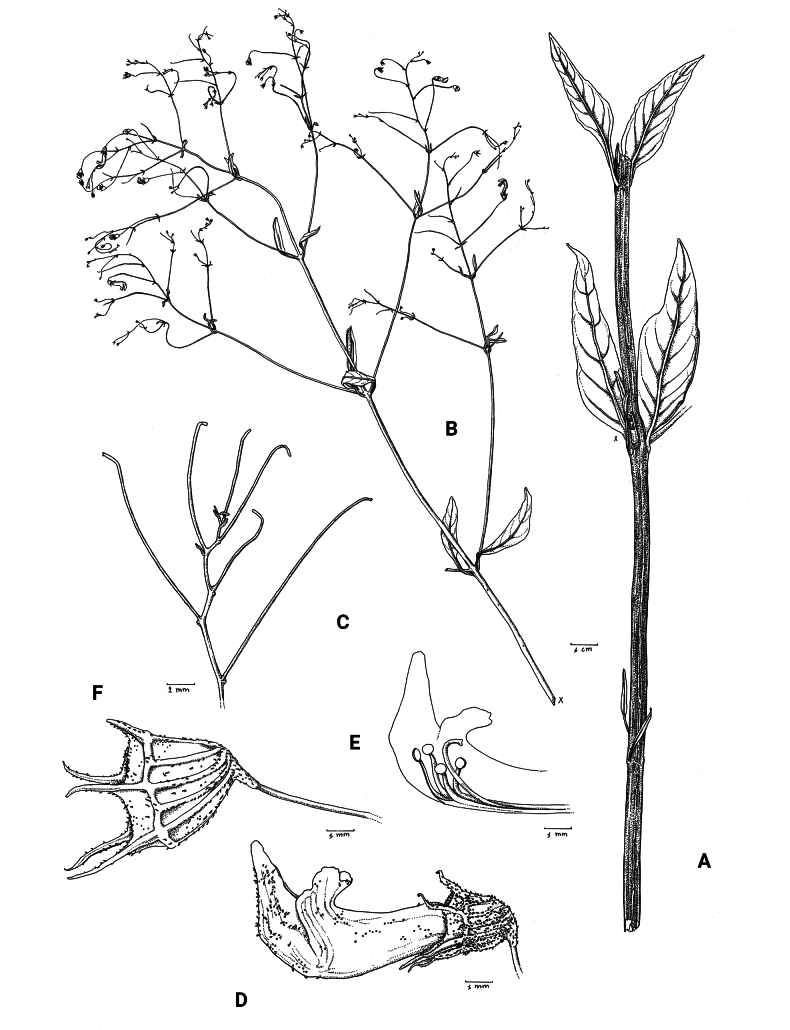
*Coleuskaminaensis* Meerts & A.J.Paton **A** stem and leaves **B** inflorescence **C** detail of inflorescence **D** flower **E** section of a corolla **F** fruiting calyx (*P.Quarré 2999*). Drawn by Hilde Orye. Scale bars: 1 cm (**A, B**); 2 mm (**C**); 1 mm (**D, E, F**).

##### Etymology.

Kamina, locality in DR. Congo where the type specimen was collected.

##### Distribution.

Endemic to SE DR. Congo, known only from the type specimen.

##### Habitat and ecology.

Savannah; ca. 1000 m elev.

##### Additional specimens.

None.

##### Notes.

1. Corolla structure, with lower lip upwardly curved, almost closing the throat and short stamens, suggest that the species is self-pollinating.

2. The single gathering of this species lacks fruiting calyces, with most flowers detaching at anthesis. Collecting notes indicate that the plant is heavily attacked by some leaf-eating parasite, virtually no leaves being untouched; this might account for the observed early abortion of flowers. The specimen is also galled. The species is said to be widespread in its locality.

3. Vernacular name: Lutoti na pori.

#### ﻿ Coleus kapatensis

Taxon classificationPlantaeLamialesLamiaceae

R.E.Fr., Wiss. Erg. Schwed. Rhod.-Kongo Exped. 1: 283. 1916.

81D72131-813E-5F4E-9B87-CF3B5C1C21EB

≡ Plectranthuskapatensis (R.E.Fr.) J.K.Morton, Novon 8: 265. 1998. Type: Zambia, Bangweulu [Bangwelo], in Peninsula Kapata pr. Kamindas, 5 Oct 1911. *R.E.Fries 871* (holotype UPS [V-046475]; isotype K, Z).

##### Description.

Paton et al. (2009: 275), Paton et al. (2013: 242), as *Plectranthuskapatensis* (R.E.Fr.) J.K.Morton.

##### Distribution.

S. Tanzania to S. Tropical Africa.

##### Habitat and ecology.

Miombo woodland, more rarely savannah on rock outcrops and dry evergreen woodland (muhulu); 1050–1570 m elev.

##### Additional specimens.

DR. Congo, ***Haut-Katanga***, Keyberg, 1 Apr 1957, *E.Detilleux 736* (BR); Luiswishi, 4 Apr 1972, *F.Malaisse 7618* (BR); Fungurume, colline Bilima, 29 Aug 2007, *I.Parmentier & Kila 4744* (BR); Kyamasumba, 42 km NNW of Kolwezi, 19 Sep 1982, *M.Schaijes 1521* (BR); 12 km NNW of Lubumbashi, 19 Apr 1962, *A.Schmitz 7711* (BR).

Burundi, Dunga (Osso), 21 Sep 1977, *M.Reekmans 6402* (BR).

##### Notes.

1. New to DR. Congo and Burundi.

2. This species has been collected in DR. Congo and Burundi either in the rainy season in vegetative state (leafy shoots bearing propagules) or completely leafless and in flowers at the end of the dry season.

#### ﻿ Coleus kivuensis

Taxon classificationPlantaeLamialesLamiaceae

Lebrun & L.Touss., Bull. Jard. Bot. État Bruxelles 17: 72. 1943.

3DC4DBC3-DAB8-5365-B0D6-1DFFEB1B79CF

≡ Plectranthuskivuensis (Lebrun & L.Touss.) R.H.Willemse, Kew Bull. 40: 96. 1985. Type: DR. Congo, Kivu, Rutshuru, Dec 1937, *J.Lebrun 9031* (holotype BR [BR0000006262563]; isotype K [K000431876], [K000431875], P [P00450798]).
= Plectranthusneochilus sensu Troupin & Ayob., Fl. Rwanda 3: 339. 1985., non Schltr.

##### Description.

Paton et al. (2009: 346), as *Plectranthuskivuensis* (Lebrun & L.Touss.) Willemse.

##### Distribution.

Eritrea to N. Tanzania and E DR.Congo.

##### Habitat and ecology.

Shrub savannah, fallow fields, steppe; 900–1400(–2200) m elev.

##### Additional specimens.

DR. Congo, ***Lacs Edouard et Kivu***, Kengele, pied du Ruwenzori, 27 Apr 1914, *J.Bequaert 3969* (BR); Keshero, 23 Sep 1958, *Crispiels-Thonon 124* (BR); Près de Kambukabakali, rive droite de la Semliki, 27 Oct 1954, *G.F.de Witte 11278* (BR); Kaliba, pied du Kasali, 11 Mar 1957, *G.F.de Witte 14035* (BR); Rutshuru, 16 Nov 1971, *C.Evrard 6827* (BR); Rutshuru, 30 Dec 1936, *J.Ghesquière 3573* (BR, K).

Rwanda, Mulehe (Bugesera), 15 Mar 1954, *L.Liben 1268* (BR, WAG); Parc national de l’Akagera, plaine de Nyaruhuru, 14 Apr 1969, *G.Bouxin & M.Radoux 167* (BR); Région du Matara, environs de Mimuli, 23 May 1957, *G.Troupin 3205* (BR); Région du Mutara, environs de Nyagatare, colline Rutare, 1 May 1958, *G.Troupin7239* (BR).

#### ﻿ Coleus kundelunguensis

Taxon classificationPlantaeLamialesLamiaceae

Meerts & A.J.Paton sp. nov.

BA1B170F-CB41-589D-A501-4C8EC306D53B

urn:lsid:ipni.org:names:77347695-1

Fig. 7A–D

##### Type.

*S.Lisowski, F.Malaisse & J.-J.Symoens 7626*, DR. Congo, Katanga, Kundelungu, 1650 m elev., steppe humide, 28 Oct 1969 (holotype POZG [POZG-V-0073133]).

##### Diagnosis.

Related to *C.foliatus*, differing in the very lax inflorescence with verticils spaced 10–25 mm, the longer pedicel jointed near the middle, the anthers not forming a pouch and the bifid style.

##### Description.

Perennial herb 0.30–0.50 m high, shoots more or less tufted, more or less woody at base, rootstock rhizomatous. Stem subterete, erect to ascending, simple or sparingly branching, shortly pubescent with patent eglandular hairs of different lengths, almost papilliform in the inflorescence, sparse glandular hairs and yellow sessile glands. Leaves opposite, ascending to erect, sessile to subsessile, ca. 4–6 pairs to a stem, widely spaced, petiole 0–0.2 cm long, blade ovate to ovate-elliptic, or obovate-elliptic, (1.2–)2.0–5.5(–7.5) × (0.5–)0.6–2.0 cm, base broadly cuneate to rounded, occasionally truncate to subauriculate in the uppermost leaves, apex acute to obtuse, more rarely acuminate, margin crenate to subentire, narrowly recurved, very shortly pubescent near margin and on lower surface of mid-vein, punctuate on both surfaces, drying dark reddish-green especially on veins. Inflorescence mostly unbranched, occasionally with a pair of branches at base, 4–20 cm long, very lax, rachis somewhat flexuose, verticils spaced 10–25 mm, flower solitary in the axil of each bract, with 1 or 2 flowers at each node, bract narrowly ovate to linear, 2–5 mm long, acute, pedicel 5–7 mm long at anthesis, elongating to 8–17 mm in fruit, very shortly pubescent (hairs almost papilliform, eglandular), conspicuously jointed and slightly angled slightly above middle, inserted asymmetrically in front of upper calyx lobe. Flower: calyx 4 mm long at anthesis, 9–10 mm long in fruit, tube straight to very slightly curved, 6–7 mm long, shortly pubescent, with eglandular and glandular hairs and sparse sessile orange glands, veins prominent, throat truncate, upper lip triangular, 1.5–2.5 mm long, slightly curving upwards, not decurrent, lower lobes narrowly triangular, the middle ones longer, 2.5–3.5 mm long, all lobes shortly ciliate. Corolla ca. 12 mm long, tube 6–7 mm long, sigmoid, lower lip carenate, 5–7 mm long, slightly pubescent on carena, upper lip 4 mm long, pubescent; anthers not forming a pouch, style bifid. Nutlets pale brown, smooth, dull, ovoid to globose, ca. 1.5 mm long.

**Figure 7.**
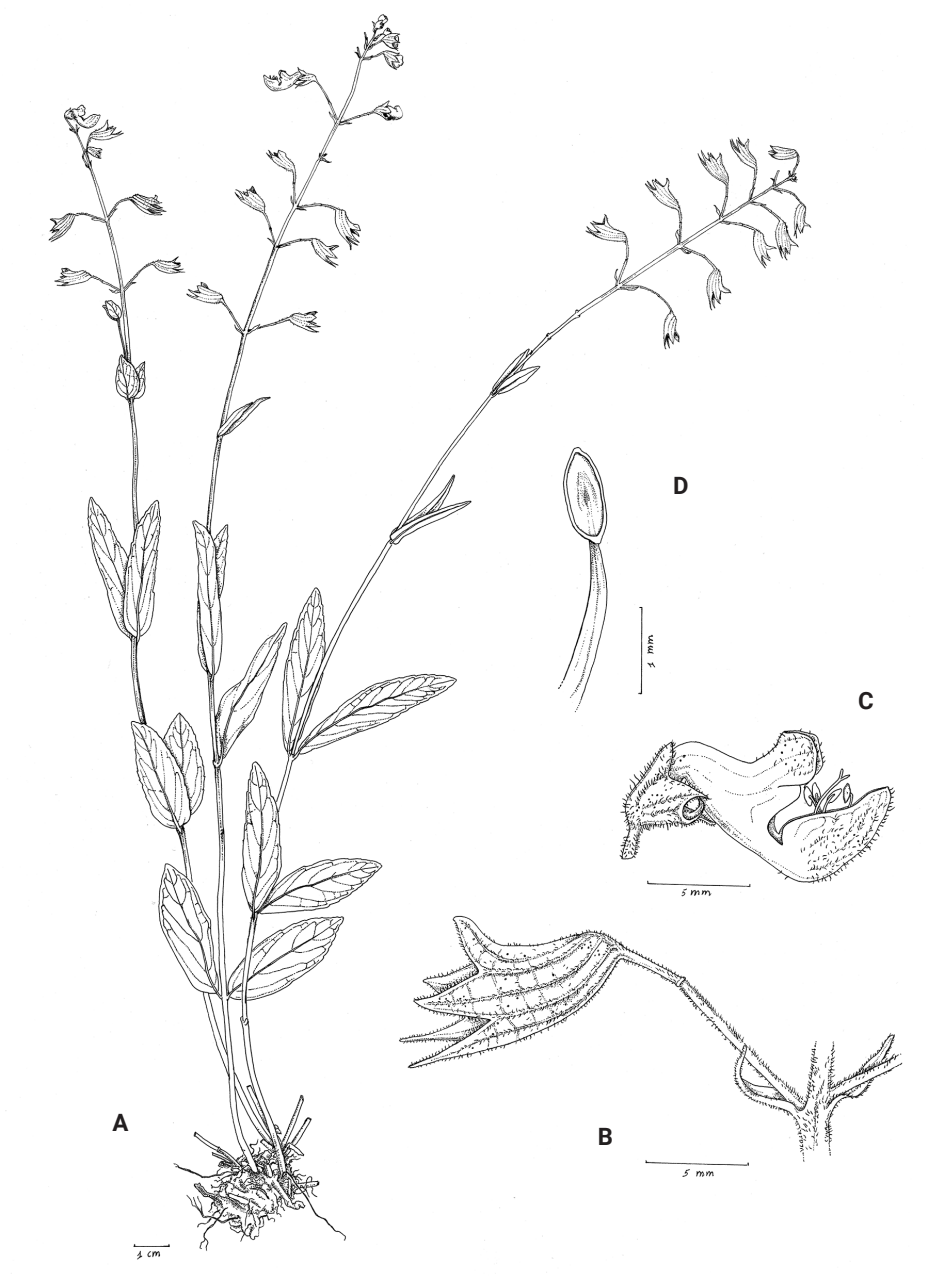
*Coleuskundelunguensis* Meerts & A.J.Paton **A** habit **B** inflorescence verticil with one fruiting calyx **C** flower **D** stamen (**A***S.Lisowski, F.Malaisse & J.-J.Symoens* 773 & *6722***B** S.*Lisowski, F.Malaisse, J.-J.Symoens 6722***C, D** S.*Lisowski, F.Malaisse, J.-J.Symoens 7468*). Drawn by Hilde Orye. Scale bars: 1 cm (**A**); 5 mm (**B, C**); 1 mm (**D**).

##### Distribution.

Endemic to the DR. Congo, Haut-Katanga, Kundelungu Plateau.

##### Habitat and ecology.

Steppic savannah, often on moist soil, 1600–1700 m elev.

##### Additional specimens.

DR. Congo, ***Haut-Katanga***, Kundelungu, près de la source occidentale de la Lutshipuka, 1600 m elev., steppe arbustive, 6 Jan 1969, *S.Lisowski 773* (POZG); Kundelungu, bord de la Kalembe, 1600 m elev., 8 Jan 1971, *S.Lisowski 23701* (POZG); Kundelungu, 1600 m elev., près du gîte Rack, 6 Feb 1969, *S.Lisowski, F.Malaisse & J.-J.Symoens 1286* (POZG); Kundelungu, rivière Lualala, 1700 m elev., à 3 km au SE du poste de Lualala, 16 Feb 1969, S.*Lisowski, F.Malaisse & J.-J.Symoens 2149* (BR, POZG) *& 2149d* (POZG); Kundelungu, 1700 m elev., à 3 km à l’W du poste de Lualala, steppe herbacée, 27 Oct 1969, *S.Lisowski, F.Malaisse & J.-J.Symoens 7468* (POZG); Kundelungu, à 3 km de la barrière, 1590 m elev., bord de la rivière Kalunda, 9 Jan 1971, *S.Lisowski, F.Malaisse & J.-J.Symoens 12592A* (POZG).

##### Notes.

1. Most of the cited specimens had been hitherto misidentified in collections as the Angolan *C.strictipes*, from which *C.kundelunguensis* differs in the smaller habit, flexuous shoot, very lax inflorescence with verticils spaced 10–25 mm, the anthers not forming a pouch and the bifid style.

2. The anthers not forming a pouch and the bifid style are rare in the group of species formerly referred to *Holostylon*, being observed only in *C.kundelunguensis* Meerts & A.J.Paton and *C.bifidus* (A.J.Paton) A.J.Paton.

#### ﻿ Coleus lactiflorus

Taxon classificationPlantaeLamialesLamiaceae

Vatke, Linnaea 43: 89. 1881.

01E0AE81-8D61-5EF5-912D-78C9A1803668

≡ Plectranthuslactiflorus (Vatke) Agnew, Upland Kenya Wild Fl.: 637. 1974. Type: Kenya, Taita District, Mbololo Forest, May 1985, *H.J.Beentje et al. 1042* (neotype K [K000975978]; isoneotype EA, designated by Paton et al. [2009]).

##### Description.

Paton et al. (2009: 351), as *Plectranthuslactiflorus* (Vatke) Agnew.

##### Distribution.

Ethiopia to N. and NW Tanzania, E DR. Congo.

##### Habitat and ecology.

Savannah fallow fields; 1400–1600 m elev.

##### Additional specimens.

DR. Congo, ***Lac Albert***, Ituri, SE of Ngolu, N of Nioka, 18 Dec 1951, *T.Sperry 335* (BR).

Rwanda, Territ. Kibungu, Rwinkwavu, 13 Apr 1966, *J.Lewalle 685* (BR).

##### Note.

1. New to DR. Congo. Rare in Central Africa, known from only two collections.

#### ﻿ Coleus lanuginosus

Taxon classificationPlantaeLamialesLamiaceae

Hochst. ex Benth. in A.P.de Candolle, Prodr. 12: 79. 1848.

12894A27-E149-5587-ABF3-F0C9B80114F0

≡ Plectranthuslanuginosus (Hochst. ex Benth.) Agnew, Upland Kenya Wild Fl.: 638. 1974.
= Coleussodalium Baker, Fl. Trop. Afr. 5: 526. 1900. Type: Eritrea, Mogad (Magod) Valley, 8 Apr 1892. *G.Schweinfurth & D.Riva 1810* (holotype K; isotype BR [BR0000006245825], FT, Z).

##### Type.

Ethiopia, in montibus et vallibus prope Aduam [Adua], 4 Oct 1842. *G.W.Schimper III.1915* (lectotype K [K000431915]; isolectotype B, BM, E, FT, G, KIEL, MPU, P, UPS, W, designated by Ryding [2000]).

##### Description.

Paton et al. (2009: 347), Paton et al. (2013: 278), as *Plectranthuslanuginosus* (Hochst. ex Benth.) Agnew.

##### Distribution.

Eritrea to N Tanzania, SW Arabian Peninsula. Naturalised in Zimbabwe.

##### Habitat and ecology.

Dry woodlands, steppe, shrub savannah; 1800–2500 m elev.

##### Additional specimens.

DR. Congo, ***Lacs Edouard et Kivu***, Rutshuru, 19 Apr 1914, *J.Bequaert 6051* (BR); Nyamgaleka, versant droit de la Haute Lume, 14 Apr 1953, *G.F.de Witte 8783* (BR); Kikomero, Nov 1937, *J.Lebrun 8447* (BR); Entre les rivières Molindi et Rutshuru, 21 Feb 1958, *M.Heine 220* (BR).

Rwanda, Kidaho, Rukoro, 29 Mar 1962, *C.Nshorere 116* (BR); Bweramvula, 20 Jun 1933, *G.Molitor 37* (BR).

##### Note.

Many materials cited in the Flore du Rwanda by Troupin and Ayobangira (1985) were errors for *Equilabiumwollastonii* (S.Moore) Mwany. & A.J.Paton.

#### ﻿ Coleus linarioides

Taxon classificationPlantaeLamialesLamiaceae

Meerts & A.J.Paton sp. nov.

65BD2E6A-ADF1-5146-8EDA-574F60E20259

urn:lsid:ipni.org:names:77347696-1

Fig. 8A–D

##### Type.

*S.Lisowski 23150* (holotype POZG [POZG-V-0072770]), DR. Congo, Haut-Shaba [Haut-Katanga], Plateau des Kundelungu, env. 3 km au NW de la source occidentale de la Lutshipuka, steppe, 11 Jan 1971.

##### Diagnosis.

Belongs in the group of species formerly comprising the genus *Holostylon* on account of the undivided style; closely related to *C.foliatus*, differing in the linear leaves, 1–4 mm wide, with strongly revolute margin.

##### Description.

Herb, annual or perennial, ca. 0.20–0.30 m high, roots not observed. Stem ascending to erect, subterete, simple, with short ascending hairs and sessile red glands, internodes ca. 1 cm. Leaves opposite or ternate, more rarely scattered, ascending to erect, sessile, blade linear, 1.5–3.5 × 0.1–0.4 cm, base truncate to rounded, apex blunt, margin strongly revolute, glabrous and punctate above, slightly pubescent on mid-vein and margins underneath and with many red sessile glands. Inflorescence simple, lax, 2–6 cm long, verticils spaced ca. 5 mm, mostly 2-flowered, rachis shortly pubescent with ascending glandular and eglandular hairs, bracts ovate, acute, ca. 3 mm long, pedicels 5–7 mm long, with short ascending hairs, without a conspicuous joint. Flower: calyx 4 mm long at anthesis, elongating to ca. 9 mm in fruit, campanulate to tubular, tube straight to slightly curved, with orange sessile glands and short glandular and eglandular hairs, upper lip ovate-triangular, slightly recurved, 2 mm long, lateral lobes of lower lip triangular, median lobes of lower lip narrowly triangular, slightly longer; corolla ca. 12 mm long, purplish, tube sigmoid ca. 5 mm long, lower lip strongly keeled, ca. 7 mm long, shortly pubescent and with sessile pale glands, upper lip ca. 4 mm long. Anthers pouch-like; style undivided. Nutlets not observed.

**Figure 8.**
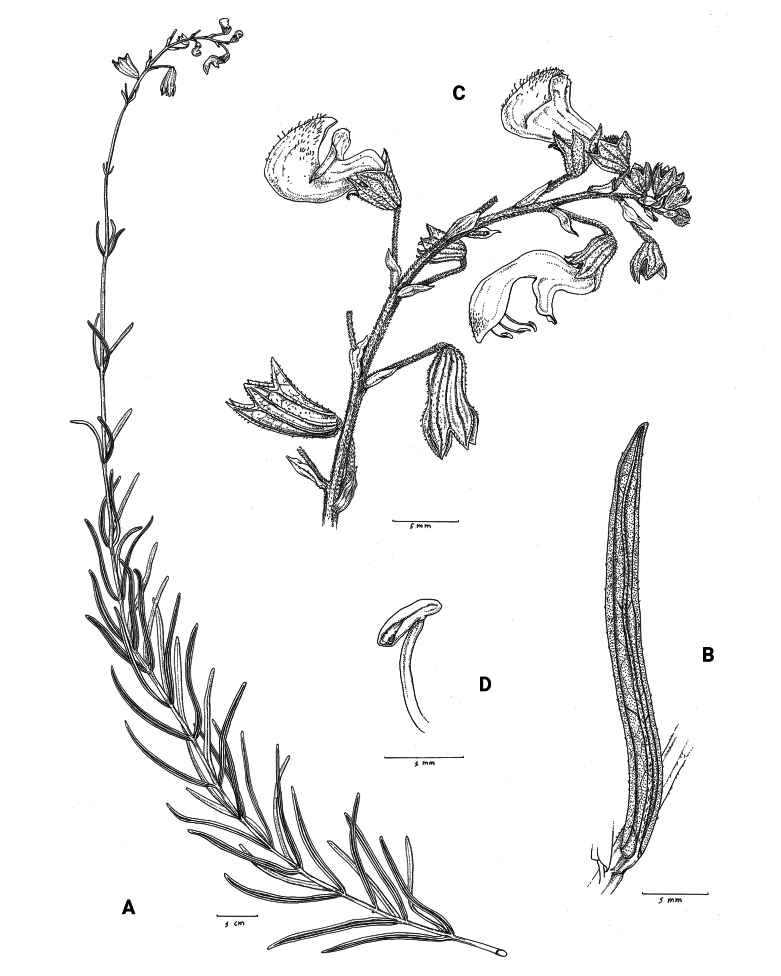
*Coleuslinarioides* Meerts & A.J.Paton **A** habit **B** leaf undersurface **C** detail of inflorescence **D** stamen (*S.Lisowski 23150*). Drawn by Hilde Orye. Scale bars: 1 cm (**A**); 5 mm (**B, C**); 1 mm (**D**).

##### Etymology.

Habitus and leaf shape are reminiscent of several species of *Linaria* (Plantaginaceae).

##### Distribution.

Endemic to DR. Congo, Haut-Katanga, Kundelungu Plateau.

##### Habitat and ecology.

Steppic savannah, ca. 1600 m elev.

##### Additional specimens.

None.

##### Note.

A very distinct species on account of the ternate linear leaves with strongly revolute margin.

#### ﻿ Coleus lisowskii

Taxon classificationPlantaeLamialesLamiaceae

Meerts & A.J.Paton sp. nov.

3BEE2006-0A84-59BC-923D-56AD8B2B81EC

urn:lsid:ipni.org:names:77347697-1

Fig. 9A–E

##### Type.

DR. Congo, Katanga, Marungu, 1900 m elev., près du poste Luonde, steppe herbacée, 15 Jun 1969, *S.Lisowski, F.Malaisse & J.-J.Symoens 5908* (holotype POZG [POZG-V-0072834]; isotype POZG [POZG-V-0072833]).

##### Diagnosis.

Belongs in the group of species formerly comprising the genus *Pycnostachys*, closely related to *Coleusdescampsii* and *Coleusparvifolius*, on account of the short, narrow leaves, the very pubescent calyx and the ciliate bracts; it differs in the lack of small leaf fascicles in the axils and in the heteromorphic leaves.

##### Description.

Perennial herb, 0.50–1.00 m high, rhizome creeping. Stem with a prostrate basal part (0–)1–10 cm, rooting at nodes, then abruptly erect, terete to obscurely quadrangular, shortly pubescent with retrorse hairs in lower half, upwardly more or less villous, also with pale sessile glands, simple or sparingly branched only near tip. Leaves opposite or ternate, sessile to very shortly petiolate, heteromorphic, the ones on the lower part of the stem spreading to recurved, ovate-elliptic, obovate-elliptic, to broadly elliptic, 1.5–3.5 × (0.5–)1.0–1.5 cm, the lowermost (juvenile) ones almost round, ca. 0.5 × 0.5 cm, base broadly cuneate to rounded, apex rounded, obtuse or subacute, margin shallowly crenate to subentire, somewhat thickened by a marginal vein, shortly ciliate, with 4 or 5 pairs of secondary veins, arching and more or less parallel to margin, prominulent on both surfaces, shortly pubescent on veins on lower surface, subglabrous on upper surface; petiole 0.1–0.3 cm; leaves on the erect part of the stem ascending to erect, more rarely recurved, subsessile, blade very narrowly lanceolate or oblanceolate-elliptic, to almost linear, 1.8–5.0 × 0.2–0.6(–0.8) cm, flat or more or less folded in length, base cuneate to truncate, apex subacute, margin subentire to shallowly crenate distally, ciliate to denticulate, veins 3–5 pairs, prominulent on both surfaces, diverging at a narrow angle and more or less parallel, subglabrous to shortly pubescent on both surfaces (antrorse hairs), more densely so in upper leaves, also with sessile orange and red glands on both surfaces. Inflorescence spicate, capitate to shortly ovoid, 12–30 × 10–16 mm (corolla excluded), apex subacute to obtuse, bracts subtending inflorescence narrowly lanceolate to almost linear, reflexed, persistent, ca. 10 × 0.8–1.5 mm, lower surface villous, upper surface glabrous; bracts of individual flowers purplish, narrowly elliptic to almost linear, the uppermost ones forming a coma, outwardly curving, ca. 4–7 × 0.5–0.8 mm, villous on abaxial surface, densely ciliate, with a fringe of undulate or curly cilia 0.5–1 mm. Flower: calyx tube ca. 2 mm long at anthesis, tomentose and with orange sessile glands, fruiting calyx tube elongating to 4–5 mm long, somewhat compressed on upper side, gibbous on lower side, villous, with long eglandular hairs, also with yellow sessile glands, lobes more or less spreading, ca. 3–4 mm long at fruiting, somewhat flattened and sharp-edged near base, with long curly eglandular hairs ca. 0.6 mm long on margins and outer surface and sessile orange glands, scales at mouth triangular ca. 0.8–1 mm long, somewhat outwardly curving at maturity, with short eglandular hairs; corolla ca. 11–15 mm long, colour unknown (pale coloured in herbarium), tube sigmoid, with a narrow basal part ca. 4 mm long and a broader distal part 3–4 mm long, lower lip 4–6 mm long, pubescent and with yellow-orange sessile glands, upper lip 3–4 mm long, with 4 narrowly triangular lobes. Nutlets pale brown, dull, smooth, ovoid, somewhat compressed, ca. 1.5 mm long.

**Figure 9.**
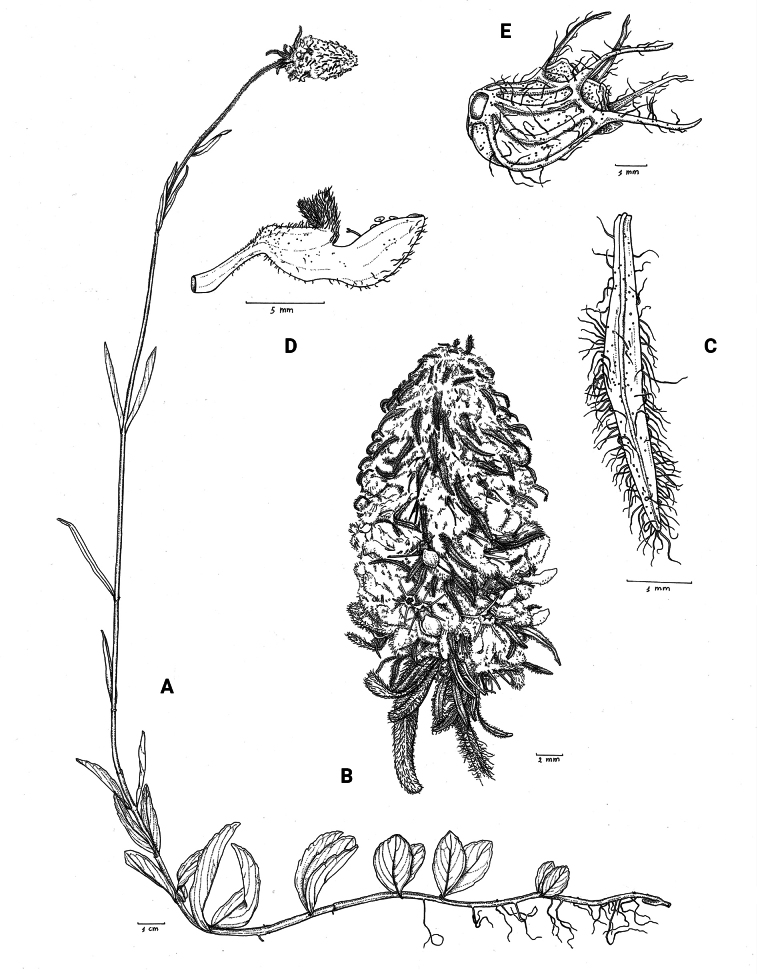
*Coleuslisowskii* Meerts & A.J.Paton **A** habit **B** inflorescence **C** bract **D** corolla **E** fruiting calyx (**A***S.Lisowski, F.Malaisse & J.-J.Symoens 6148a***B–E** S.*Lisowski, F.Malaisse, J.-J.Symoens 5908*). Drawn by Hilde Orye. Scale bars: 1 cm (**A**); 2 mm (**B**); 1 mm (**C, E**); 5 mm (**D**).

##### Etymology.

Dedicated to Stanislaw Lisowski (1924–2002), Polish botanist, who made important contributions to the flora of tropical Africa.

##### Distribution.

Endemic to the DR. Congo, Haut-Katanga, Marungu Massif.

##### Habitat and ecology.

Steppic savannah, woodlands, dambos, ca. 1400–2000 m elev.

##### Additional specimens.

DR. Congo, ***Haut-Katanga***, Marungu, à env. 3 km à l’W de Kasiki, 12 Jun 1969, *S.Lisowski, F.Malaisse & J.-J.Symoens 6148a* (POZG); Plateau de Muhila, bord de la rivière Muizia, 13 May 1971, *S.Lisowski 23728* (POZG); Marungu, 20 km NE de Kapulo, dembo frais dans la forêt claire à *Brachystegia*, 25 Jun 1957, *P.Duvigneaud 3710P* (BRLU); Entre Kapulo et Pepa, base du plateau des Marungu, muulu autour d’une termitière, 25 Jun 1957, *P.Duvigneaud 3713bis* (BRLU).

##### Notes.

1. This species is superficially similar to *C.lancifolius*, on account of the narrow ascending leaves, differing in the capitate inflorescence, the tomentose calyx and the long ciliate bracts. The prostrate basal part of the stem, with broader leaves, is less typical in *P.Duvigneaud 3710P* & *3713bis*, but the four cited collections are quite similar in all the other characters and there is little doubt that they are conspecific.

2. There are intermediates with *C.descampsii*, with very short leaves in some axils, for example, S.*Lisowski 23727, 23728* (POZG), Plateau de Muhila, bord de la rivière Muizia, 1450 m elev., 13 May 1971; *S.Lisowski 23719* (POZG), Plateau de Muhila, 5 km N du poste de Kitu, 1420 m elev., bord marécageux d’un ruisseau, 16 May 1971.

3. *S.Bidgood et al. 3358* (K, P), from Tanzania, is probably the same taxon.

#### ﻿ Coleus longipetiolatus

Taxon classificationPlantaeLamialesLamiaceae

Gürke, Bot. Jahrb. Syst. 19: 214. 1894.

3075595A-087E-5B93-A89F-EDA8EA1D82AA

= Plectranthusleptophyllus (Baker) A.J.Paton, Fl. Trop. E. Afr., Lamiac.: 284. 2009. Type: Kenya, Ribe (Ribi) to Galla country, *T.Wakefield s.n.* (holotype K [K000431957]).

##### Type.

Tanzania, Usambara Mts, Kwa Mshusa, 15 Aug 1893, *C.Holst 9076* (holotype B destroyed; isotype COI [COI00005780], G, HBG [HBG518665], K [K000431956], KFTA, M [M0104723], P [P00450800], W, Z).

##### Description.

Paton et al. (2009: 284), Paton et al. (2013: 231), as *Plectranthusleptophyllus* (Baker) A.J.Paton.

##### Distribution.

E & S Tropical Africa.

##### Habitat and ecology.

Wooded savannah, forest; 1040–1300 m elev.

##### Additional specimens.

DR. Congo, ***Lacs Edouard et Kivu***, Virunga, Katuka, 26 May 1948, *J.de Wilde 70* (BR); Virunga, Rivière Mati, affluent gauche de la rivière Talya, 24 Apr 1956, *G.F.de Witte 13179* (BR); Karunda, Nyabiondo, 24 Jul 1957, *R.Gutzwiller 1369* (BR).

##### Note.

New species record for DR. Congo.

#### ﻿ Coleus maculosus

Taxon classificationPlantaeLamialesLamiaceae

(Lam.) A.J.Paton, Phytokeys 129: 68. 2019.

461847B3-1D19-56F7-B2CF-2AC4192D67C7

≡ Galeopsismaculosa Lam., Encycl. 2: 601. 1788. Type: cultivated, from Africa. (holotype P-Lam).

##### Note.

*Coleusmaculosus* is variable in petiole length, leaf blade shape and corolla length. Earlier authors (e.g. Robyns (1947): Troupin and Ayobangira (1985)), recognised three taxa: a taxon with large corolla and oblong sessile leaves (corresponding to the type specimen of *Coleusedulis* Vatke), a taxon with large corolla and ovate petiolate leaves (type of *Coleusfimbriatus* Lebrun & L.Touss.) and a taxon with small corolla and ovate petiolate leaves (type of *Plectranthuspunctatus* L’Hér.). Recent authors, however, give priority to corolla size and recognise only two subspecies; we follow this treatment, but further work is needed.

### ﻿﻿Key to the subspecies of *Coleusmaculosus*

**Table:** 

1	Corolla 6–8 mm long; tube 3–5 mm long, almost straight to shallowly sigmoid; leaf petiolate, blade ovate	**C.maculosussubsp.maculosus**
–	Corolla (11–)13–18 mm long; tube 6–8 mm long, shallowly to conspicuously sigmoid; leaf sessile to petiolate, blade ovate to oblong-elliptic	**C.maculosussubsp.edulis**

#### ﻿ Coleus maculosus (Lam.) A.J.Paton, subsp. maculosus

Taxon classificationPlantaeLamialesLamiaceae

D471C34D-62E8-53F2-905C-FDD2112250DC

= Coleusserrulatus Robyns, Bull. Jard. Bot. État Bruxelles 17: 78. 1943.
≡ Plectranthusserrulatus (Robyns) Troupin & Ayob., Bull. Jard. Bot. Natl. Belg. 55: 299. 1985. Type: DR. Congo, Visoke, R. Susa, 2 Feb 1935, *G.F.de Witte 2214* (holotype BR [BR0000006263263], [BR0000006262624]; isotype K [K000431883]).
= PlectranthuspunctatusL’Hér.subsp.punctatus, Fl. Trop. E. Afr., Lamiac.: 350. 2009. Type: Ethiopia (“Abyssinia”), cultivated from seed sent by Bruce at Hort. Kew, 1774 (neotype BM; isoneotype G; designated by Hedge et al. [1998]).

##### Description.

Paton et al. (2009: 349), as Plectranthuspunctatus(L.f.)L’Hérit.subsp.punctatus.

##### Distribution.

Cameroon, to Eritrea and Tanzania, Madagascar.

##### Habitat and ecology.

Marshland, mountain grassland, fallow field, mountain forest, regrowth; 1700–3050 m elev.

##### Additional specimens.

DR. Congo, ***Lacs Edouard et Kivu***, Beni, Vayana, Aug 1938, *P.Gille 108* (BR); Entre les Lacs Kivu et Edouard, Apr-May 1929, *H.Humbert 7907* (BR); Mont Kahuzi, 13 Jun 1971, *S.Lisowski 23824* (POZG).

Rwanda, Piste allant de Kinigi au pied du Visoke, 3 Feb 1972, *P.Auquier 2415* (BR); Flanc sud du Sabyinayo, 3 Feb 1972, *P.Bamps 3071* (BR); Remera, Buliza, Kigali, Nov 1932, *A.Becquet 326* (BR); Bunyereri, Nyungwe, 13 May 1971, *G.Bouxin 698* (BR); Forêt de Mushabarara, Apr 1939, *G.Gilbert 2361* (BR); Rukura, Bumbogo, Mar 1933, *G.Molitor 23* (BR); Mont Kisoni, 6 Mar 1935, *J.B.Lejeune 210* (BR); Route Bukavu-Astrida [Butare], km 94, 4 May 1959, *G.Troupin 9909* (BR); Ruhengeri, Kinigi, 24 Feb 1972, *P.Van der Veken 9530* (BR).

Burundi, Kitega, 4 Dec 1922, *O.A.J.Elskens 214* (BR); Bujumbura, Mayuyu, 28 Feb 1971, *J.Lewalle 5249* (BR); Muramwya, Ryarusera, 27 Feb 1972, *M.Reekmans 1577* (BR); Rwasave, rivière Murwuya, 14 May 1957, *D.van der Ben 1567* (BR).

#### ﻿ Coleus maculosus subsp. edulis

Taxon classificationPlantaeLamialesLamiaceae

(Vatke) A.J.Paton, Phytokeys 129: 69. 2019.

DD1FC474-E26D-5AA9-8F6C-215C012FAC9B

≡ Coleusedulis Vatke, Linnaea 37: 319. 1872.
≡ Plectranthusedulis (Vatke) Agnew, Upland Kenya Wild Fl.: 640. 1974.
≡ Coleusmaculosussubsp.edulis (Vatke) A.J.Paton (2019) 69. Type: Ethiopia (“Abyssinia”), near Gaffat, Oct 1863, *G.W.Schimper 1212* (holotype B destroyed; isotype BM [BM000514932], K [K000431925], [K000431923]).
≡ Plectranthuspunctatussubsp.edulis (Vatke) A.J.Paton, Fl. Trop. E. Afr., Lamiac.: 350. 2009.
= Coleusfimbriatus Lebrun & L.Touss., Bull. Jard. Bot. État Bruxelles 17: 79. 1943.
≡ Plectranthusfimbriatus (Lebrun & L.Touss.) Troupin & Ayob., Bull. Jard. Bot. Natl. Belg. 55: 299. 1985. Type: DR. Congo, Mt Mushumangabo, Aug 1937, *J.Lebrun 7163* (holotype BR [BR0000006262945], [BR0000006263270]).

##### Description.

Paton et al. (2009: 350), as Plectranthuspunctatussubsp.edulis (Vatke) A.J.Paton.

##### Distribution.

Ethiopia, Kenya, Uganda, Tanzania, Burundi, Rwanda and DR. Congo.

##### Habitat and ecology.

Mountain forest, savannah, fallow field, marshland, swamp, river banks, riparian forest; 1600–3100 m elev.

##### Additional specimens.

DR. Congo, ***Lac Albert***, Nioka, 27 Oct 1934, A.P.De Craene 229* (BR, WAG); ***Lacs Edouard et Kivu***, Tshirunge, 5 Oct 1914, *J.Bequaert 5997* (BR); Parc National Albert [Virunga], volcan Niamlagyra, aux environs de Mushumangabo, 17 Jan 1942, *R.Germain 1258* (BR); W du Lac Kivu, Feb-Mar 1929, *H.Humbert* 7527* & 7528 (BR); Mont Kahuzi, 28 May 1960, Meurillon 953* (BR, LWI); ***Haut-Katanga***, Parc National de l’Upemba. Lubanga, 17 Apr 1948, *G.F.de Witte 03726* (BR).

Rwanda, Mukono, Byumba, Dec 1932, A.Becquet 189* (BR); Rwasenkoko, route Butare-Cyangugu, km 67, 1 Mar 1980, *D.Bridson 468* (BR, K); Gikongoro, Kivu, Rubyiro, 16 Aug 1999, C.Ewango & Ngayabahiga 2195* (BR, GIS, M, MO, WAG); Route Astrida [Butare]-Shangugu, km 65, 7 Mar 1958, *M.Reynders 239* (BR); Route Bukavu-Astrida [Butare], env. Uwinka, colline Bunyereri, 7 Aug 1959, G.Troupin 10566* (BR).

Burundi, Muramvya, Mont Manga Mugongo, 4 Jun 1966, *J.Lewalle 883* (BR); Muramvya, Nyabigonde, 3 Apr 1966, J.Lewalle 642* (BR); Nyakirwa, 21 Jan 1977, M.Reekmans 5647* (BR); Province: Ngozi, Commune: Mukora (Rwegura), 27 Apr 1977, *M.Reekmans 6046* (BR); Ijenda, 8 May 1981, M.Reekmans 10137* (BR, WAG).

##### Note.

Subsp. edulis is variable in petiole length and blade shape; morphs with subsessile leaves and oblong-elliptic blade up to 14 cm long (specimens indicated “*” hereabove) correspond to the type of *C.edulis*; morphs with ovate, petiolate leaves correspond to the type of *C.fimbriatus*.

#### ﻿ Coleus mannii

Taxon classificationPlantaeLamialesLamiaceae

Hook.f., J. Proc. Linn. Soc., Bot. 7: 211. 1864.

CCB37847-33A4-56E8-9458-B71FBD49E709

= Coleusgiorgii De Wild., Bol. Soc. Ibér. Ci. Nat. 19: 120. 1920. Type: DR. Congo, Likimi, *S.De Giorgi 1511* (lectotype BR [BR0000021453984], designated by Champluvier & Dowsett-Lemaire [1999]).

##### Type.

Cameroon, Mt Cameroon, 1862, *G.Mann 1967* (holotype K [K000025010]).

##### Description.

Paton (2022: 51).

##### Distribution.

W Tropical Africa to DR. Congo and Sudan.

##### Habitat and ecology.

Swamps, riparian forest, savannah on wet soil, often near watercourses and ponds; 400–1200 m elev.

##### Additional specimens.

DR. Congo, ***Bas-Congo***, Mandzambe, s.d., *J.Claessens 326* (BR); ***Kasaï***: Kebiya, Dec 1951, *Flamigni 10350* (BR); Bokoro, 22 May 1948, *Jans 695* (BR); Luluabourg [Kananga], 1930, *H.Vanderyst 21130 & 21135* (BR); ***Forestier central***, Yaekama, territ. Isangi, 7 Feb 1959, *P.Bamps 337* (BR); Avakubi, 9 Jan 1914, *J.Bequaert 1847* (BR); Ligasa-Mangala, 9 Dec 1956, *C.Evrard 2045* (BR); Banzingi, 22 Jul 1954, *G.F.de Witte 10739* (BR); Marais de la Bukotsa, 27 Nov 1952, *H.Fredericq* in *G.F.de Witte 8421* (BR); 6 km W de Yangambi, 6 Jul 1938, *J.Louis 10212* (BR, P); ***Ubangi-Uele***, Dungu, rivière Nambasa, Oct 1936, *A.M.De Graer 749* (BR); Entre Banzyville [Mobayi-Mbongo] et Pambwa, 14 Oct 1954, *C.Evrard 109bis* (BR); Entre Libenge et Gemena, Dec 1930, *J.Lebrun 1841* (BR); Dida, Gombari, 22 Dec 1906, *F.Seret 711* (BR); ***Lacs Edouard et Kivu***, Virunga, Bakotsa, près confluent Byangolo-Molidi, 23 Sep 1954, *G.F.de Witte 11129* (BR); Sinamboro, affl. droit Balembi, 4 Feb 1955, *G.F.de Witte 11668* (BR).

#### ﻿ Coleus marunguensis

Taxon classificationPlantaeLamialesLamiaceae

Meerts & A.J.Paton sp. nov.

1DA42DD8-88CC-560D-9F9B-AF232B1675D3

urn:lsid:ipni.org:names:77347698-1

Fig. 10A–F

##### Type.

DR. Congo, Haut-Katanga, Kasiki, 20–27 Jun 1931, *G.H.de Witte 493* (holotype BR [BR0000016835344]).

##### Diagnosis.

Closely related to the species formerly comprising the distinct genus *Solenostemon* (middle lobes of lower calyx lip fused over most of their length), differing in the beige tomentose flowers, the stem with antrorse hairs, the shortly petiolate leaves with blade truncate at base, upper surface glabrous and with impressed veins.

##### Description.

Perennial herb or shrub, woody in lower half, height unknown (> 0.40 m). Stem erect, branched, markedly quadrangular, very shortly puberulent with appressed antrorse eglandular hairs and sparse long patent hairs in lower part and also with sparse red sessile glands. Leaves opposite, spreading, petiolate, with or without fascicles of small leaves in the axils; petiole 0.7–1.6 cm long, pubescent like the stem or villous; blade discolorous, broadly ovate-triangular, 2.0–5.0 × 1.4–4.0 cm, base truncate to slightly cordate, apex acute, margin serrate, recurved, teeth rounded, lower surface with very short appressed hairs on veins and softly pubescent to sparsely villous between the veins and with red sessile glands, upper surface subglabrous, veins impressed on upper surface and prominent on lower surface. Inflorescence 5–20 cm long, lax, verticils spaced 10–25 mm, cymes sessile, cincinni 1–2 mm long (pedicel not included), 6–8 flowered, bracts puberulous on outer surface, ovate, cucullate, contracted into a point, ca. 4 mm long, forming a small apical coma, pedicel ascending, 3–4 mm long in fruit, curving at tip, inserted very eccentrically on calyx in front of upper lobe. Flower: calyx ca. 1.5 mm long at anthesis, densely beige tomentose and with red sessile glands, 4–5 mm long in fruit, pubescent all over, tube ca. 1.5 mm long, contracted at throat, sulcate, upper lip purplish, obovate, ca. 2 mm long, curving upwards, obtuse-rounded, margin often irregularly undulate and with 1 tooth on either side and with a short apical mucro, lateral lobes oblong, ca. 1.5 mm long, truncate to rounded, lower middle lobes fused into a lip ca. 2.5 mm long, with two ovate apical lobes contracted into a point; corolla wholly beige tomentose when in bud, ca. 9–13 mm long at anthesis, pubescent to tomentose and with sparse red sessile glands, tube slightly sigmoid, ca. 3.5 mm long, lower lip 4–8 mm long, 2–3 mm deep, cucullate, upper lip 1.5–3 mm long, 4-lobed; stamen filaments fused, anther ca. 0.7 mm long, style bifid. Nutlets not observed.

**Figure 10.**
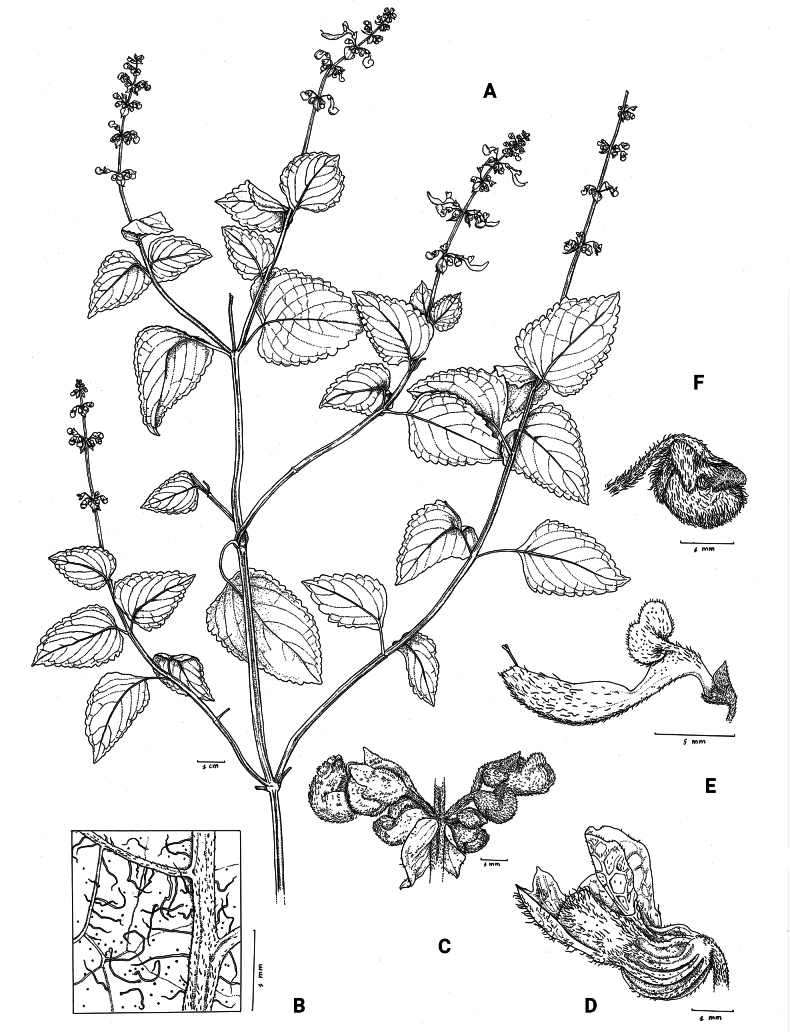
*Coleusmarunguensis* Meerts & A.J.Paton **A** habit **B** detail of pubescence of leaf undersurface **C** detail of inflorescence **D** fruiting calyx **E** flower **F** flower bud (**A, B, C, E***G.H.de Witte 493***D, F***S.Lisowski, F.Malaisse & J.-J.Symoens 10339a*). Drawn by Hilde Orye.Scale bars: 1 cm (**A**); 1 mm (**B, C, D, F**); 5 mm (**E**).

##### Etymology.

The Marungu Plateau, where the type specimen was collected, is a hotspot of biodiversity in DR. Congo.

##### Distribution.

Endemic to DR. Congo (Haut-Katanga, Marungu Plateau).

##### Habitat and ecology.

Wetlands, 1850–2200 m elev.

##### Additional specimens.

DR. Congo, ***Haut-Katanga***, Plateau des Marungu, Env. de Luonde, mare Buzanza, 1850 m elev., 20 Feb 1970, *S.Lisowski, F.Malaisse & J.-J.Symoens 10339* (POZG).

##### Note.

There is a second specimen in BR labelled “*G.F.de Witte 493*” [BR0000016835351]; however, it is a different species, i.e. *Equilabiumstolzii*, that was probably growing intermingled with *Coleusmarunguensis*. *Equilabiumstolzii* is known to occur in wetlands on the Marungu Plateau; the second sheet has been labelled by us *G.F.de Witte 493bis*.

#### ﻿ Coleus melleri

Taxon classificationPlantaeLamialesLamiaceae

(Baker) A.J.Paton & Phillipson, Phytokeys 129: 73. 2019.

8198A2A6-D78D-5428-9A4C-4978C9C2EE03

≡ Plectranthusmelleri Baker, J. Bot. 20: 243. 1882. Type: Madagascar, between Toamasina (Tamatave) and Antananarivo, 3 Aug 1862. *C.J.Meller s.n.* (holotype K [K000430814]).
= Plectranthusluteus Gürke, Bot. Jahrb. Syst. 28: 468. 1900.
≡ Coleusluteus (Gürke) Staner, Bull. Agric. Congo Belge 25: 426. 1934. Type: Tanzania, Morogoro District: SE Uluguru Mts, *F.Stuhlmann 8790* (holotype B destroyed).

##### Description.

Paton et al. (2009: 293), Paton et al. (2013: 242), as *Plectranthusmelleri* Baker.

##### Distribution.

Liberia, Gabon, Uganda to S. Tropical Africa, Madagascar.

##### Habitat and ecology.

Dense evergreen mountain forest, riparian forest, Ericaceae scrub; 850–2600 m elev.

##### Additional specimens.

DR. Congo, ***Kasaï***, Kwango, 21 Jul 1955, *R.Devred 2317* (BR); ***Lac Albert***, Lekwa (Djugu), 11 Mar 1959, *Deville 229* (BR); Mt. Dia, 13 Oct 1937, *G.C.Gilbert 557* (BR); ***Lacs Edouard et Kivu***, Lake Kivu, Idjwi Island, 6 Aug 1959, *Cambridge Congo Expedition 275* (BR, US); Ruwenzori, versant ouest, vallée de la Lume, Jul 1929, *H.Humbert 8980* (BR, P); Terr. Kabare, Kahuzi, 11 Feb 1959, *A.Léonard 2981* (BR, WAG); Wimbi, 26 km S Lubero, 21 Jul 1937, *J.Louis 4631* (BR); Mont Biega, 10 Aug 1972, *Ntakiyimana 318* (BR, LWI); Route Bukavu-Walikale, km 48, 17 Mar 1960, *J.Petit 9* (BR, LWI); Zwischen Nyamuragira und Mikeno, 17 Sep 1964, *H.U.Stauffer 371* (BR, WAG, Z). ***Haut-Katanga***, Lusinga, route Mitwaba, 14 Sep 1948, *W.Robyns 3591* (BR); Kundelungu, 10°26'S, 27°53'E, 5 Oct 1950, *A.Schmitz 3201* (BR).

Rwanda, Près de Pindura, piste de l’Ibigugu, 29 Jul 1974, *P.Auquier 3526* (BR); Flanc sud Sabyinyo, 3 Jul 1972, *P.Bamps 3049* (BR); Uwinka, Nyungwe, 9 Aug 1969, *G.Bouxin & M.Radoux 554* (BR); Volcan Sabyinyo, 4 Feb 1972, *G.Troupin 14339* (BR, WAG); Route Butare-Cyangugu, 2 km avant Gisakura, 24 Aug 1974, *P.Van der Veken 10942* (BR, GENT, WAG).

Burundi, Bugarama, 29 Jun 1969, *J.Lewalle 3860* (BR); Bururi, rives de la Siguvyaye, 2 May 1966, *J.Lewalle 761* (BR, MO, WAG); Bujumbura, Mt. Manga, 14 Sep 1947, *M.Reekmans 3520* (BR).

##### Notes.

1. The specimen *W.Robyns 3591* (BR) from Haut-Katanga (Upemba) is larger than usual in all its parts including corolla and leaf blade base is obtuse-rounded instead of acute-attenuate; it could be a different taxon.

2. The specimen *A.Léonard 3526* (BR) differs in having a white corolla (fide collector), being almost glabrous in all parts and lacking propagules; it is almost certainly a distinct taxon, but more materials are needed.

#### ﻿ Coleus meyeri

Taxon classificationPlantaeLamialesLamiaceae

(Gürke) A.J.Paton, Phytokeys 129: 73. 2019.

7BD64AFF-8138-51AA-B259-A327BB480ADF

≡ Pycnostachysmeyeri Gürke, Abh. Königl. Akad. Wiss. Berlin 1891: 362. 1892. Type: Tanzania, Kilimanjaro, Rua stream, *H.Meyer 279* (holotype B destroyed; isotype K [K000405982] fragment).
= Pycnostachyslongibracteata De Wild., Pl. Bequaert. 4: 388. 1928. Type: DR. Congo, Ruwenzori, Vallée du Lanuri, 26 Mar 1914, *J.Bequaert 4490* (holotype BR [BR0000008909763]; K fragment).
= Pycnostachysovoideoconica De Wild., Pl. Bequaert. 4: 396. 1928. Type: DR. Congo, Mukule–Mokoto, *J.Bequaert 6325* (holotype BR [BR0000008910097]; K fragment).

##### Description.

Paton et al. (2009: 412), as *Pycnostachysmeyeri* Gürke.

##### Distribution.

W. Tropical Africa to Ethiopia and Tanzania.

##### Habitat and ecology.

Savannah, Bambusa thickets, mountain forest, riparian forest; 1500–2400 m elev.

##### Additional specimens.

DR. Congo, ***Lac Albert***, Blukwa, Sep 1949, *A.P.De Craene 351* (BR, K); Aye Kibali, Djugu, 11 Jun 1959, *D.Froment 520* (BR); ***Lacs Edouard et Kivu***, Ruwenzori, Lanuri, 26 May 1914, *J.Bequaert 4490* (BR, K fragment); Mukule-Mokoto, 19 Dec 1914, *J.Bequaert 6325* (BR, K fragment); Terr. Masisi, Dondo, May 1957, *R.Gutzwiller 1063* (BR, K); Ruwenzori, vallée de la Muboka, Nov 1931, *J.Lebrun 4412* (BR); Kahuzi, 1 Jul 1959, *A.Leonard 4816* (BR, K); Flanc NW du Karisimbi, 17 Aug 1937, *J.Louis 5250* (BR); Mont Bugulumiza, 26 Jul 1955, *R.Pierlot 665* (BR); Busenene, 13 Nov 1953, *R.Van Ysacker 75* (BR).

Rwanda, Wisumo, Gisovu, 21 Feb 1980, *D.Bridson 433* (BR); Route Astrida [Butare]-Bukavu, km 93, *G.Troupin 11533* (MO); Env. Rangiro, Kirambo, 4 Jun 1981, *G.Troupin 16271* (BR); Ruhengeri, Kinigi, 24 Feb 1972, *P.Van der Veken 9531* (MO).

Burundi, Mont Teza, 9 Jul 1974, *J.Rammeloo 3753* (BR).

#### ﻿ Coleus mirabilis

Taxon classificationPlantaeLamialesLamiaceae

Briq., Bot. Jahrb. Syst. 19: 183. 1894.

92F0A564-C73D-555E-B40A-E8D09850E2CA

≡ Ascocarydionmirabile (Briq.) G.Taylor, J. Bot. 69 (suppl. 2): 162. 1931.
≡ Plectranthusmirabilis (Briq.) Launert, Mitt. Bot. Staatssamml. München 7: 299. 1968. Type: Angola, Malanje (Malandsche), Mar 1880, *A.von Mechow 489* (lectotype Z not seen; isolectotype W [W 1889-0054590], designated by Codd [1975]).
= Coleusmirabilisvar.poggeanus Briq., Bot. Jahrb. Syst. 19: 184. 1894. Type: DR. Congo, River Lulua, 9°30'S, *P.Pogge 350* (holotype B destroyed).

##### Description.

Paton et al. (2013: 258), as *Plectranthusmirabilis* (Briq.) Launert

##### Distribution.

S DR. Congo to NE Namibia.

##### Habitat and ecology.

Swamp savannah, riparian forest; ca. 900–1100 m elev.

##### Additional specimens.

DR. Congo, ***Kasaï***, Panzi, 1925, *H.Vanderyst 16117* (BR); ***Bas-Katanga***, Mutombo-Mukulu, Jun 1931, *P.Quarré 2534* (BR); Kamina, Lovoi, Apr 1932, *P.Quarré 3007* (BR); ***Haut-Katanga***, Manika, route Kolwezi-Kasaji, 4 Apr 1955, *J.Brynaert 393* (BR); Territ. Dilolo, Kisenge, marais de la Mukuleshi, en bordure de galerie forestière, 19 May 1957, *P.Duvigneaud 3237La* (BRLU).

#### ﻿ Coleus minusculus

Taxon classificationPlantaeLamialesLamiaceae

Meerts & A.J.Paton sp. nov.

A0229AF4-DE5A-53BA-8A8D-6592EF14D2C5

urn:lsid:ipni.org:names:77347699-1

Fig. 11A–C

##### Type.

DR. Congo, Haut-Katanga, 28 km NE de Lubumbashi, Savane de la Luiswishi, 1208 m elev., 30 Aug 1972, *J.Bulaimu 520* (holotype BR [BR0000017733526]).

##### Diagnosis.

Related to *Coleusmodestus* on account of inflorescence structure, differing in being a dwarf plant (< 10 cm high), leafless at flowering, rootstock a small tuber.

##### Description.

Perennial herb, 0.04–0.10 m, leafless at flowering, glutinous; rootstock a fusiform tuber 1–2 × ca. 0.5 cm. Stem 1 or several, purplish, erect, simple or branched, terete to subquadrangular, with dense short glandular hairs and sparse eglandular hairs. Leaves not observed. Inflorescence terminal, lax, racemiform, flowers solitary in the axil of each bract, mostly subopposite, bracts linear, ca. 1 mm long, persistent, ciliolate; pedicel 2–3 mm long, extending to 4–5 mm in fruit, slightly ascending to patent, slightly curving downwards at tip, inserted slightly eccentrically on calyx. Flower: calyx 2–2.5 mm long at anthesis, to 4 mm in fruit, with yellow-orange sessile glands and short glandular hairs, tube shortly cylindrical to campanulate, 10-veined, straight or slightly curved upwards, lobes subequal, narrowly triangular, ca. 1 mm long, the upper one slightly broader, slightly recurved, not decurrent; corolla 5–7 mm long, bluish, with yellow-orange sessile glands, tube straight, ca. 2 mm long, progressively broadening to throat, lower lobe 2.5–4 mm long, ca. 2 mm deep, upper lobe ca. 1.5 mm long, 4-lobed; anther ca. 0.6 mm; style bifid. Nutlets pale brown, ca. 0.9 mm diam., smooth, flattened, red-speckled.

**Figure 11.**
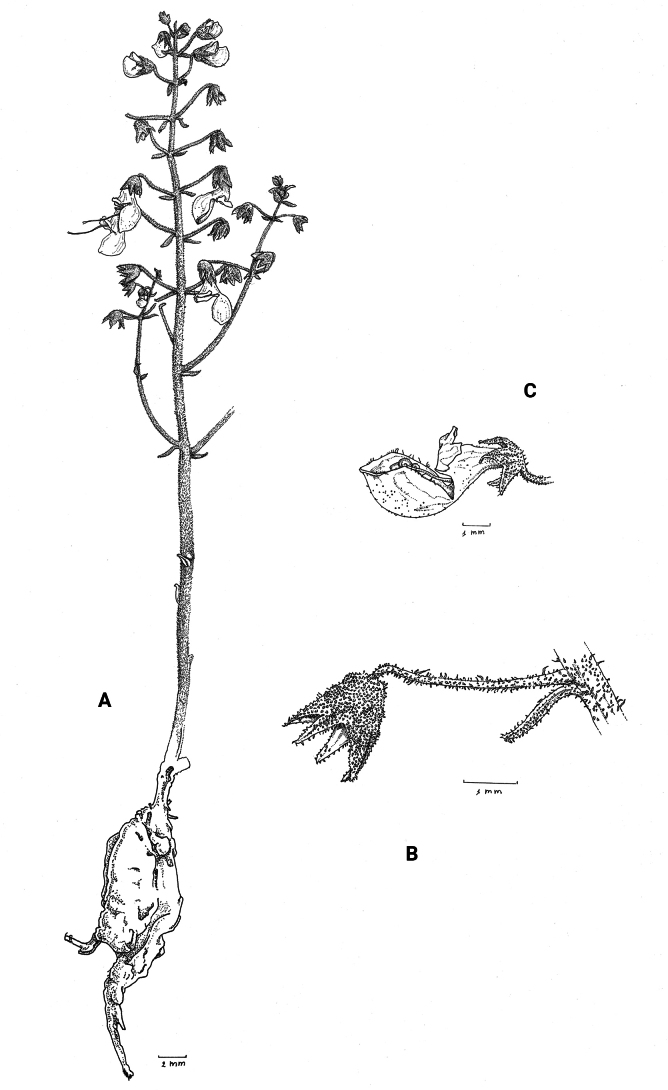
*Coleusminusculus* Meerts & A.J.Paton **A** habit **B** fruiting calyx **C** flower. *J.Bulaimu 520*. Drawn by Hilde Orye.Scale bars: 2 mm (**A**); 1 mm (**B, C**).

##### Etymology.

Latin *minusculus -a -um*, very small, on account of the dwarf habit of the species.

##### Distribution.

Endemic of SE DR. Congo (Haut-Katanga).

##### Habitat and ecology.

Miombo woodlands, savannah; 1200–1300 m elev.

##### Additional specimens.

DR. Congo, ***Haut-Katanga***, Guba (territ. Lubudi), 30 km E of Kolwezi, dépression incendiée sur terre très dure dans les Mutobo [*Isoberlinia* div. sp.], 29 Aug 1956, *P.Duvigneaud & J.Timperman 2549Co* (BRLU); Luiswishi, savane, 20 Sep 1982, *F.Malaisse 12350* (BR).

##### Note.

*C.minusculus* is a very distinctive species, on account of its dwarf habit. It shares similarities with *C.modestus*, which also has tubers, a single flower in the axil of each bract, often subopposite and inflorescence with glandular hairs.

#### ﻿ Coleus mitwabaensis

Taxon classificationPlantaeLamialesLamiaceae

Meerts & A.J.Paton sp. nov.

9557BFB0-574D-55BB-A804-F5AB0B36A425

urn:lsid:ipni.org:names:77347700-1

Fig. 12A–F

##### Diagnosis.

Differing from all other species by the following combination of traits: corolla yellow, rootstock a small tuber, leaves not exceeding 2.7 × 1.0 cm.

##### Type.

DR. Congo, Haut-Katanga, Kaziba (rive gauche Mweleshi, affluent rive gauche Senze), 1140 m elev., forêt katangaise, 12 Feb 1948, *G.F.de Witte 3333* (holotype BR [BR0000017707961]; isotype K).

##### Description.

Perennial herb, with a single shoot, 0.12–0.40(–0.50) m high, from a globose to irregularly knobby ovoid tuber ca. 10 mm diam. Stem erect or more or less straggling, puberulent, with very short patent or retrorse eglandular hairs and yellow sessile glands; inflorescence axis abruptly becoming densely covered with purplish papillae and very short glandular hairs. Leaves opposite, spreading to ascending, occasionally grouped near stem base, blade mostly elliptic to narrowly elliptic, the lowermost ones shorter, obovate to obovate-elliptic, (1.0–)1.5–2.7 × (0.1–)0.3–1.0 cm, base cuneate to attenuate, apex obtuse to rounded, subglabrous above or strigillose (with short upward pointing hairs), very shortly appressed pubescent on veins beneath, with pale sessile glands, margin shallowly crenate to entire, very narrowly recurved, papillate to ciliolate, ca. 3 pairs of secondary veins diverging at a very acute angle; petiole 0(–0.3) cm. Inflorescence terminal, lax, unbranched or with 1 or 2 branches at lowermost node, 5–15 cm long, rachis with short papilliform hairs, glandular and eglandular, often reddish tinged, verticils spaced 5–15 mm, 2-flowered, flowers solitary in the axil of each bract, bract narrowly ovate, ca. 1 mm long, more or less persistent, pedicel 1–2 mm long, inserted slightly eccentrically. Flower: calyx at right angle with the pedicel, tubular, with pale sessile glands, short glandular hairs and conical papilliform purplish hairs on veins, ca. 3 mm long at anthesis, accrescent to 4–5 mm in fruit, throat truncate, posterior lobe ovate-triangular, ca. 1–1.5 mm long, not decurrent, lateral lobes triangular ca. 2 mm long, median lobes of lower lip narrowly triangular, ca. 2.5 mm long, with the sinus between median lobes deeper than between median and lateral lobes. Corolla yellow, 10–13 mm long, tube slightly curved to almost straight, widening from base to throat, ca. 3 mm diam. near throat, upper lip ca. 1 mm long, lower lip shortly pubescent, with yellow sessile glands and thin flexuous marginal cilia, ca. 4 mm long, cucullate, ca. 2 mm deep, enclosing stamens. Nutlets globose, very slightly compressed, brown, ca. 1 mm diam.

**Figure 12.**
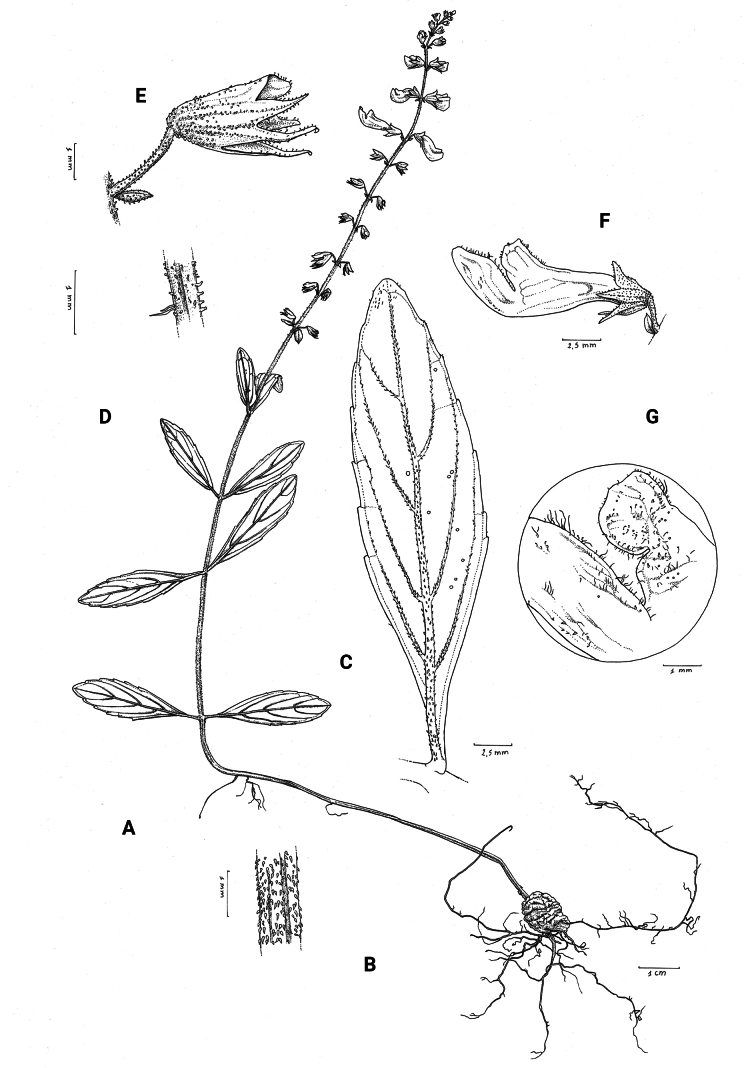
*Coleusmitwabaensis* Meerts & A.J.Paton **A** habit **B** detail of stem pubescence **C** leaf undersurface **D** detail of rachis pubescence **E** fruiting calyx **F** flower **G** detail of corolla pubescence. *P.Bamps & F.Malaisse 8613*. Drawn by Hilde Orye. Scale bars: 1 cm (**A**); 1 mm (**B, D, E, G**); 2.5 mm (**C, F**).

##### Etymology.

Mitwaba Plateau, in the north of Upper Katanga, where the type specimen was collected, hosts a very original flora.

##### Distribution.

Endemic of SE DR. Congo (Haut-Katanga, Mitwaba Plateau).

##### Habitat and ecology.

Savannah, seasonally moist soil on river banks, miombo woodland; 1140–1400 m elev.

##### Additional specimens.

DR. Congo, ***Haut-Katanga***, Route Mitwaba-Manono, km. 45, riv. Kalumengongo (zone Mitwaba), 1140 m elev., 8°19'S, 27°16'E, sable périodiquement inondé en bordure de rivière, 3 Feb 1986, *P.Bamps & F.Malaisse 8613* (BR); Kankunda (affl. rive gauche du Lupiala), 1400 m elev., forêt, 26 Nov 1947, *G.F.de Witte 3104* (BR); Route automobile pour le Shinkulu, 1450 m elev., savane arbustive, 21 May 1948, *G.F.de Witte 3863* (BR, K).

##### Notes.

1. *C.mitwabensis* is strikingly distinct on account of the yellow corolla, tuberous rootstock, small leaves and 2-flowered verticils.

2. Vernacular name: tulamalama, sansala (in kiluba).

3. The sap is used to impregnate fishing nets to attract fish. The tubers are edible.

#### ﻿ Coleus modestus

Taxon classificationPlantaeLamialesLamiaceae

(Baker) Robyns & Lebrun, Ann. Soc. Sci. Bruxelles, Sér. B 49: 106. 1929.

B949B8EE-1154-5953-B37A-C0C187D7730C

≡ Plectranthusmodestus Baker, Bull. Misc. Inform. Kew 1895(99): 72 (1895). Type: Zambia, Mbala District: Tanganyika Plateau, 1889, *A.Carson s.n.* (holotype K [K000430767]).

##### Description.

Paton et al. (2009: 282), Paton et al. (2013: 229), as *Plectranthusmodestus* Baker.

##### Distribution.

SW Tanzania to Zambia.

##### Habitat and ecology.

Highland savannah, dry woodlands, dambos; 1250–1950 m elev.

##### Additional specimens.

DR. Congo, ***Haut-Katanga***, Keyberg, 6 Dec 1956, *E.Detilleux 222* (BR); Marungu, près de Kibobwa, 8 Nov 1970, *S.Lisowski, F.Malaisse, J.-J.Symoens 11875* (BR, POZG); Forêt de la Luiswishi (NE Lubumbashi), 3 Dec 1971, *F.Malaisse 7595* (BR); Marungu, Mulongoshi, Nov 1945, *P.Quarré 7211* (BR); Kimilolo, 23 Jun 1927, *P.Quarré 938* (BR); Kipopo, 12 Apr 1958, *A.Schmitz 6018* (BR); Kiunda, Sept 1944, *L.Dubois 1260* (BR).

#### ﻿ Coleus monostachyus

Taxon classificationPlantaeLamialesLamiaceae

(P.Beauv.) A.J.Paton, Phytokeys 129: 76. 2019.

B5811009-F2AD-5D3D-8B35-07D6348B7BBC

##### Type.

Benin, *A.Palisot de Beauvois s.n.* (holotype G [G00018077]).

#### ﻿ Coleus monostachyus (P.Beauv.) A.J.Paton subsp. monostachyus

Taxon classificationPlantaeLamialesLamiaceae

A4DF915B-7FB8-5C16-B33D-61F7521FBB60

= Solenostemonmonostachyusvar.amplifrons Briq. Bull. Herb. Boissier, sér. 2, 6: 826. 1906. Type: DR. Congo, Bangala, 26 May 1888, *F.Hens ser. C, 33* (holotype Z [Z-000021117]).

##### Description.

Paton et al. (2022: 52).

##### Distribution.

W Tropical Africa to S Chad and Cabinda.

##### Habitat and ecology.

Disturbed ground, cultivated fields, fallow fields, rainforest, wetland, occasionally epiphytic on palm stipe; 60–800 m elev.

##### Additional specimens.

DR. Congo, ***Mayumbe***, Gimbi, vallée de la Mvuzi, 5 Jan 1949, *L.Toussaint 720* (BR); ***Bas-Congo***, Kinsonia, 9 Mar 1960, *P.Compère 1589* (BR); Kitobola, 15 May 1910, *A.Flamigni 176* (BR, US); Terr. Masina, Kimbangu, 20 Dec 1978, *L.Pauwels 6093* (BR, WAG); ***Kasaï***, Kutu, 30 Oct 1903, *Em.&M.Laurent s.n*. (BR); Kapanga, Apr 1934, *F.Overlaet 1213* (BR); ***Forestier central***: Mulumbela, 27 Jan 1980, *Y.&T.Ankei 79/1042* (BR); Bambesa, 16 Sep 1958, *A.Blomme 122* (BR); Bongabo, 6 Jun 1971, *H.Breyne 1695* (BR); Territ. Banalia, route Kole-Kanwa, km 24, 13 Jun 1932, *Galdermans 10* (BR); Territ. Ekota-Bakutu, Sep 1934, *L.Dubois 565* (BR); Karawa, Apr 1924, *V.G.Goossens 4485* (BR); Bokuma, 26 Feb 1941, *G.Hulstaert 61* (BR); Eala, 22 May 1946, *J.Léonard 164* (BR); Yangambi, 29 Oct 1913, *A.Michiels 32* (BR); Barumbu, 29 Aug 1938, *J.Louis 11061* (BR, U, US); ***Ubangi-Uele***, Bodangabo, 15 Feb 1955, *C.Evrard 200* (BR); Digba, 13 Sep 1952, *P.Gérard 252* (BR); Tukpwo, 9 Jul 1959, *P.Gérard 3984* (BR);

##### Note.

Specimens with unusually large leaves (e.g. *W.Robyns 4263*) correspond to “var. amplifrons’, but variation is continuous and this variety is not retained here.

#### ﻿ Coleus mystax

Taxon classificationPlantaeLamialesLamiaceae

Meerts & A.J.Paton sp. nov.

7E587E6A-1A4A-5293-AE33-D8155321C6FF

urn:lsid:ipni.org:names:77347701-1

Fig. 13A–E

##### Type.

DR. Congo, Haut-Katanga, Parc national de l’Upemba, Ganza, près de la riv. Mware, 24 Jun 1949, *G.F.de Witte 6990* (holotype BR [BR0000016835726]; isotype K).

##### Diagnosis.

Closely related to *Coleusefoliatus* De Wild., differing in the long patent smooth hairs on the calyx, pedicel and rachis, the shorter, more congested, inflorescence and the calyx lobes all similar in shape.

##### Description.

Annual herb, ca. 0.3 m high, leafless at flowering. Stem erect, sharply quadrangular, puberulent (hairs appressed, antrorse), with a tuft of hairs at the nodes, shiny, with yellow sessile glands, sparingly branched. Leaves not observed. Inflorescence lateral, moderately congested, 6–20 mm long, peduncle ca. 5 mm long, 9 to 20 flowers arranged in a spiral, with a single flower between an opposite or subopposite pair, each bract subtending a single flower; rachis with straight, smooth 1–4 mm long long cilia and short glandular hairs; bract narrowly elliptic, ca. 0.3 mm long; pedicel 1–2.5 mm long, pubescent as the rachis, inserted eccentrically in front of upper calyx lobe; calyx tubular, 3–4 mm long at anthesis, slightly accrescent to 4–5 mm long in fruit, pale whitish-green with dark green veins, with patent smooth cilia 2–5 mm long, short glandular hairs and sessile yellow glands, tube truncate, lobes purplish-tinged, all lobes almost similar in shape and length, narrowly triangular-subulate, 1–2 mm long, upper lobe slightly recurved, not decurrent; corolla sky-blue, ca. 6 mm long, tube straight, ca. 3 mm long, not exceeding calyx, progressively widening to throat, upper lip ca. 1 mm long, sparingly pilose, anterior lip ca. 2 mm long, 1 mm deep, cucullate, sparingly pilose, stamens included. Fruit: Nutlets rounded, compressed, shiny brown, ca. 0.8 mm diam.

**Figure 13.**
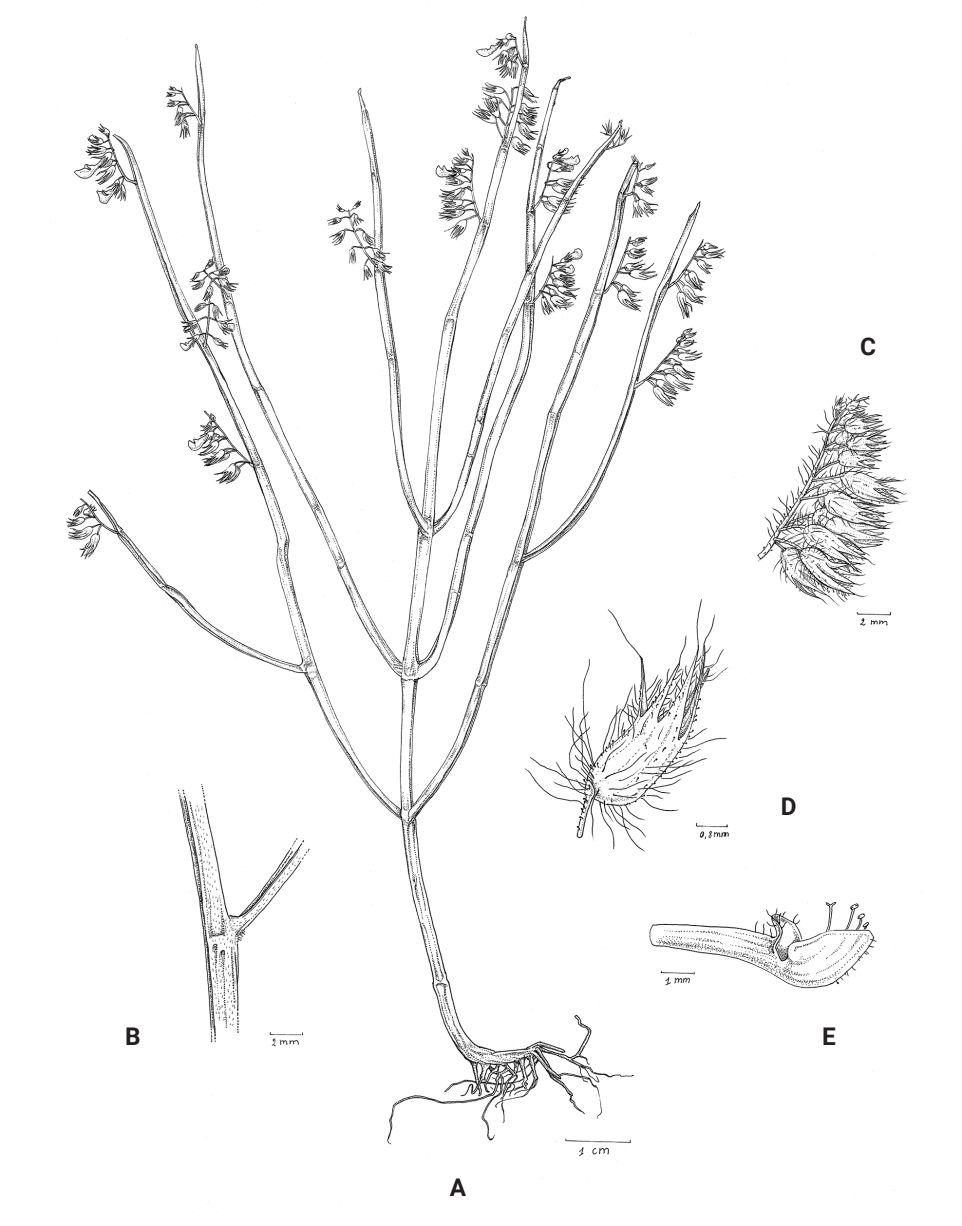
*Coleusmystax* Meerts & A.J.Paton **A** habit **B** detail of stem **C** inflorescence **D** fruiting calyx **E** corolla. *G.F.de Witte 6990*. Drawn by Hilde Orye. Scale bars: 1 cm (**A**); 2 mm (**B, C**); 0.8 mm (**D**); 1 mm (**E**).

##### Etymology.

Latin *mystax -acis*, whisker, on account of the long stiff hairs on calyx and pedicel.

##### Distribution.

Endemic of SE DR. Congo (Haut-Katanga).

##### Habitat and ecology.

Steppic savannah, miombo woodland, often on rocky soil; 1100–1400 m elev.

##### Additional specimens.

DR. Congo, ***Haut-Katanga***, Colline de Kungumarembe, à l’Est de Kasompi, forêt claire à *Brachystegiafloribunda*, 30 Jul 1956, *P.Duvigneaud & J.Timperman 2183E* (BRLU); Entre Shinkolobwe et Tantara, forêt claire sur roches dures, 7 Sep 1956, *P.Duvigneaud & J.Timperman 2643H* (BRLU); Jadotville [Likasi], forêt claire à *Brachystegiautilis* sur grand conglomérat, 15 Jun 1957, with *Coleusefoliatus*, *P.Duvigneaud 3580C1* (BRLU); 7 km à l’ouest de Nzilo, sur la piste Nzilo-Kyamasumba, 1200 m elev., forêt claire sur affleurement rocheux, 17 Jun 1984, *M.Schaijes 2323* (BR).

##### Notes.

1. *C.mystax* is closely related to *C.efoliatus*, with which it sometimes co-exists; it shares with it the annual habit, early deciduous leaves, strongly quadrangular stem, inflorescence architecture and short corolla. It differs from it in the very conspicuous long smooth cilia on inflorescence (vs. cilia lacking or occasionally sparse in *C.efoliatus*), the calyx lobes all more or less equal in size and shape (vs. upper lobe ovate), the shorter, more congested inflorescence (6–20 mm long vs. 15–50 mm) and the shorter calyx (4–5 mm long vs. 5–10 mm).

2. Vernacular name: tulamalama (in the Upemba National Park).

#### ﻿ Coleus parvifolius

Taxon classificationPlantaeLamialesLamiaceae

(Baker) Meerts & A.J.Paton comb. nov.

1F9F991E-D024-5983-82A1-D6003135DD34

urn:lsid:ipni.org:names:77347702-1

Pycnostachys parvifolia Baker, Bull. Misc. Inform. Kew 1895: 72. 1895. Type: Zambia, Northern Province, Fwambo, 1894, *A.Carson 103* (lectotype K [K000405989], designated by Bruce [1940]).(Basionym)
= Coleuscapitatus A.J.Paton, Phytokeys 129: 33. 2019, nom. illeg., syn. nov.

##### Description.

Paton et al. (2009: 388), Paton et al. (2013: 317), as *Pycnostachysparvifolia* Baker.

##### Distribution.

W and S Tanzania to Zambia and SE DR. Congo.

##### Habitat and ecology.

Steppic savannah on highlands, on moist soil (1200–) 1700–2000 m elev.

##### Additional specimens.

DR. Congo, ***Haut-Katanga***, Kundelungu, 1968, *T.Coget 116* (BR); 50 km NE Lubumbashi, bord de la Luiswishi, 11 Apr 1969, *S.Lisowski 23447* (POZG); Lwamisamba, 28 Mar 1975, *F.Malaisse 8641* (BR); Marungu, Kipiri, Nov 1945, *P.Quarré 7337* (BR).

##### Note.

1. *C.parvifolius* and *C.descampsii* are closely related and intermediates occur.

2. The new name *C.capitatus* A.J.Paton was superfluous because the binomial *Coleusparvifolius* has never been published before.

#### ﻿ Coleus pengbelensis

Taxon classificationPlantaeLamialesLamiaceae

Meerts & A.J.Paton sp. nov.

86FA9AC9-7178-565B-B518-B3823D90DA9E

urn:lsid:ipni.org:names:77347703-1

Fig. 14A–I

##### Type.

DR. Congo, Bas-Uele, Route Digba-Gwane, 700 m, sur pengbele, Dec. 1945, *R.Germain 4397* (holotype BR [BR0000016836068]; isotype K).

##### Diagnosis.

Related to *Coleusbojeri* and other species formerly referred to the genus *Solenostemon* on account to the lower calyx lobes fused into a lip, differing in the upper calyx lobe narrowly ovate-triangular, long attenuate, horizontal or somewhat recurved, much exceeding the lower lobes, indumentum papillate and tuberculate nutlets.

##### Description.

Annual herb ca. 0.3–1.0 m high, not reported to be aromatic; tubers absent or not collected. Stem erect, quadrangular, thinly papillate, simple or branched. Leaves opposite, ascending, often with fascicles of small leaves in the axils; blade narrowly ovate to narrowly elliptic, 3.5–6 × 0.8–1.6(–2.2) cm, base attenuate, apex acute, margin crenate, densely papillate on both surfaces and with sessile red glands, 4–6 pairs of veins diverging at a very acute angle, veins prominent on lower surface; petiole 0.5–1.6(–2.5) cm long, narrowly canaliculate, occasionally with long patent cilia. Inflorescence terminal, simple, (3–)9–16 cm long, lax, verticils spaced 5–25 mm, the uppermost ones contiguous, cymes consisting of two opposite cincinni, ca. 20–50 flowered, subsessile, very short at first, markedly elongating up to 30 mm in fruit and then flexuous, bracts narrowly to broadly ovate, ca. 5–7 × 2–5 mm, cucullate, occasionally with long cilia in lower half, contracted into a 2 mm point, persistent, the uppermost ones forming a coma, the others reflexed; pedicel 0.5 mm long at anthesis, elongating to 1.5 mm in fruit, inserted eccentrically in front of posterior lobe. Flower: calyx campanulate to shortly tubulate, 2 mm at anthesis, accrescent up to 4–5 mm in fruit, very shortly pubescent and with red sessile glands, tube truncate, upper lobe narrowly ovate-triangular, long attenuate, horizontal or somewhat recurved, much exceeding the lower lobes, margin occasionally ciliate, shortly pubescent also on inner surface, lateral lobes of lower lip triangular, ca. 1.2 × 1 mm, obtuse, median lobes fused for most of their length into a triangular lip ca. 1.5 × 1.5 mm, with 1 or 2 apical teeth; corolla blue or pale mauve, ca. 8 mm long, tube ca. 3 mm long, slightly curved, upper lip perpendicular, ca. 2–3 mm long, lower lip ca. 5 mm long, 2 mm deep, shortly pubescent, occasionally with sessile red glands, stamens fused. Nutlets almost globular, ca. 0.9 mm in diam., light brown, verrucose-tuberculate.

**Figure 14.**
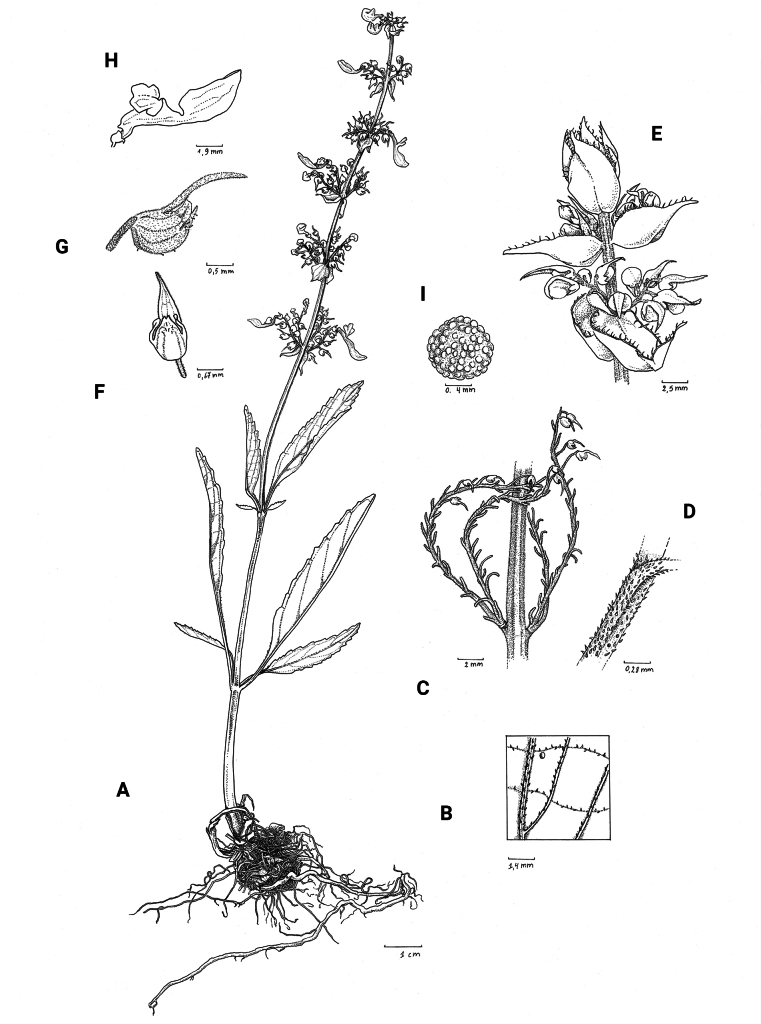
*Coleuspengbelensis* Meerts & A.J.Paton **A** habit **B** detail of lower surface of leaf blade **C** detail of infrutescence **D** detail of pubescence of inflorescence branch **E** detail of tip of inflorescence with bracts **F** flowering calyx seen from below **G** fruiting calyx, side view **H** corolla **I** nucule (**A***A.De Wulf 264* & *G.Le Testu 338* (roots) **B***A.De Wulf 264***C***G.Le Testu 338***E***A.De Wulf 264***D, F** I *R.Germain 4397*). Drawn by Hilde Orye. Scale bars: 1 cm (**A**); 1.4 mm (**B**); 2 mm (**C**); 0.28 mm (**D**); 2.5 mm (**E**); 0.67 mm (**F**); 0.5 mm (**G**); 1.9 mm (**H**); 0.4 mm (**I**).

##### Etymology.

Pengbele (also spelled pembele) is the local name of the particular vegetation type in which the species occurs i.e. open herbaceous vegetation on seasonally flooded lateritic crust (Bamps 1975).

##### Distribution.

N DR. Congo, Central African Republic.

##### Habitat and ecology.

Open savannah, scrub, seasonally moist soil on lateritic crust with impaired drainage in the rainy season and drying out in the dry season (pengbele); 600–700 m elev.

##### Additional specimens.

DR. Congo, ***Ubangi-Uele***, Région de Gwane, sur pengbele, 8 Jul 1955, *R.Boutique 148* (BR); Bas-Uélé, en savane, 13 Oct 1934, *A.De Wulf 264* (BR); Tukpwo, sur pengbele, 1 Aug 1953, *P.Gérard 989* (BR); Tukpwo, savane, 17 Apr 1954, *P.Gérard 1269* (BR); Tukpwo, sur cuirasse latéritique, Nov 1945, *R.Germain 4281* (BR); Tukpwo, un peu au-delà de la bifurcation de Bengo, îlot forestier sur pembele, 18 Jun 1942, *Gilbert* in *H.Dubois 100* (BR).

Central African Republic, ***Oubangui-Chari***, Haute-Kotto, entre Zazo et Lutari (?), plateau de latérite, 30 Oct 1921, *G.Le Testu 3385* (BR); Région de Zemio-Obo, à Obo, 27 Dec 1963, *B.Descoings 11936* (MPU); Entre Kitessa et Zemio, 01 Jan 1964, *B.Descoings 12365* (MPU); entre Zemio et Rafaï, dalle latéritique suintante à 21 km à l’ouest de Zemio, 01 Jan 1964, *B.Descoings 12407* (MPU); route entre Zemio et Rafaï, à 48 km à l’ouest de la Ouarra, 03 Jan 1964, *B.Descoings 12502* (MPU).

##### Notes.

Vernacular name: akonki-pia (in azande).

#### ﻿ Coleus penicillatus

Taxon classificationPlantaeLamialesLamiaceae

(A.J.Paton) A.J.Paton, Phytokeys 129: 82. 2019.

27051D57-D7DF-5989-8DDD-613DF3A19D24

≡ Plectranthuspenicillatus A.J.Paton, Fl. Trop. E. Afr., Lamiac.: 325. 2009. Type: Zambia, Mbuzi–Kaluza, 23 Aug 1938, *P.J.Greenway & C.G.Trapnell 5627* (holotype K [K000431999]).

##### Description.

Paton et al. (2009: 325), Paton et al. (2013: 265) as *Plectranthuspenicillatus* A.J.Paton.

##### Distribution.

S Tanzania to E Zambia and SE DR. Congo.

##### Habitat and ecology.

Rock outcrop (in DR. Congo; elsewhere: savannah). ca. 2300 m elev., (elsewhere 300–800 m elev.).

##### Additional specimens.

DR. Congo, ***Haut-Katanga***, Marungu, 12 km ESE Kasiki, 23 Sep 1959, *A.Schmitz 6577* (BR).

##### Notes.

1. New species record for DR. Congo.

2. The specimen cited was collected at a much higher elevation compared to the rest of the range (2300 m vs. 300–800 m). It departs somewhat from other materials in having denser inflorescence, with more flowers.

#### ﻿ Coleus piscatorum

Taxon classificationPlantaeLamialesLamiaceae

Meerts & A.J.Paton sp. nov.

2AC39AC4-A0F1-5B83-888A-50EB4EDE12FB

urn:lsid:ipni.org:names:77347704-1

Fig. 15A–D

##### Type.

DR. Congo, Haut-Katanga, Upemba National Park, Munoï, bifurcation Lupiala, 890 m elev., 2 Jun 1948, *G.F.de Witte 3897* (holotype BR [BR0000017708043]).

##### Diagnosis.

Closely related to *Coleusefoliatus* De Wild., differing in the shorter pedicels (1.5 vs. 2–6 mm), shorter fruiting calyx (4.5 mm long vs. 5–10 mm), with all 5 subequal triangular lobes (vs. upper lobe ovate) and the longer corolla (ca. 10 mm long vs. 3–5(–6) mm); also closely related to *C.mystax*, differing in the lack of long cilia in the inflorescence, the longer corolla and the divaricate branching pattern.

##### Description.

Annual herb, ca. 0.30 cm high. Stem erect, sharply quadrangular, shiny, sparsely pubescent, with very short retrorse and long patent hairs, with a tuft of hairs at nodes, branched in the upper two-thirds, with 3–5 pairs of opposite branches, almost horizontal to ascending at a broad angle, divaricate, slender, 1–8 cm long, each with 1 or 2 levels of dichotomous ramifications. Leaves almost all absent at flowering; blade ovate, ca. 2 × 1 cm, base rounded, apex narrowly subobtuse, somewhat pubescent on both surfaces, with pale sessile glands on lower surface, margin entire, secondary veins ca. 2 pairs, inconspicuous; petiole 0–1 mm long. Inflorescence seemingly terminal, actually lateral on ultimate node of twigs, slightly congested, 3–7 mm long, racemiform, rachis with short patent eglandular and glandular hairs, 1(–2) flower(s) in the axil of each bract, occasionally subopposite, bracts linear, ca. 1 mm long, pedicels 1–1.5 mm long, pubescent as rachis, inserted slightly eccentrically in front of upper calyx lobe. Flower: calyx ca. 2.5 mm long at anthesis, with short patent glandular and eglandular hairs and pale sessile glands, fruiting calyx ca. 4.5 mm long, whitish-membranous or chartaceous, tube tubular to campanulate, ca. 2 mm long, all lobes more or less similar in shape and size, narrowly triangular, ca. 2–2.5 mm long, acute, with thickened margin; median lobes of lower lip slightly longer; corolla blue, ca. 10 mm long, tube straight, ca. 3 mm long, progressively expanding to throat, lower lobe ca. 5 mm long, 3 mm deep, cucullate, enclosing stamens, thinly puberulent, upper lobe ca. 2 mm long. Nutlets pale brown, shiny, smooth, somewhat lenticular, ca. 1 mm.

**Figure 15.**
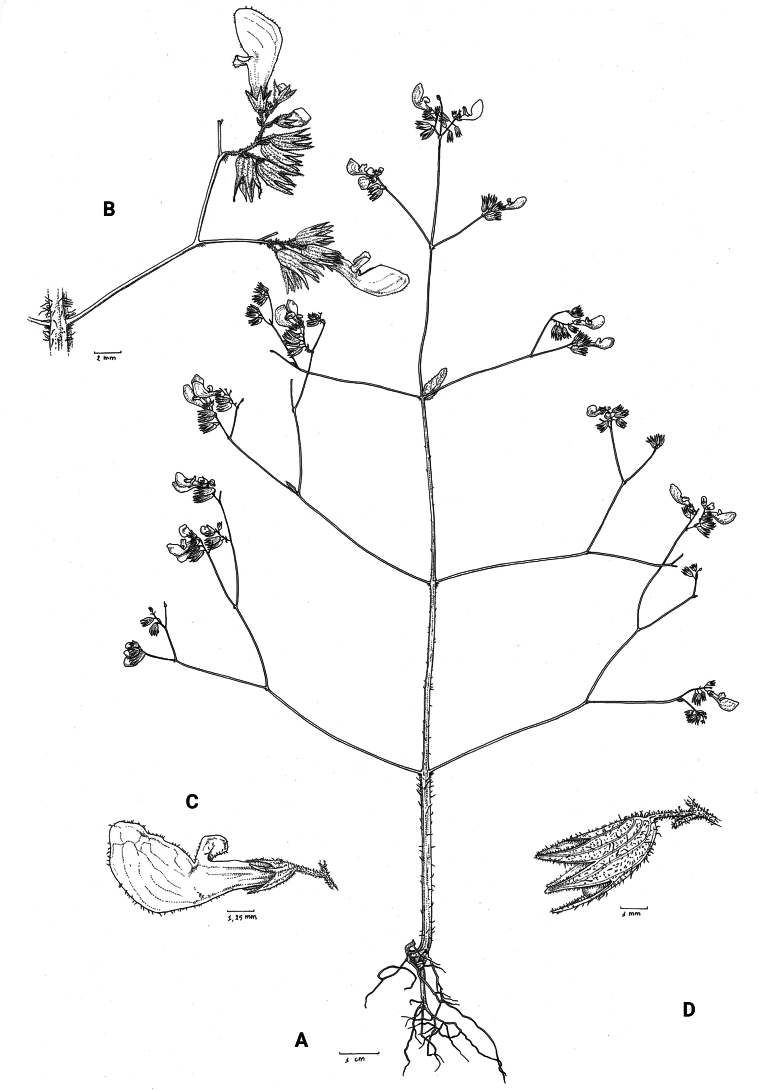
*Coleuspiscatorum* Meerts & A.J.Paton **A** habit **B** detail of inflorescence **C** flower **D** fruiting calyx (*G.F.de Witte 3897*). Scale bars: 1 cm (**A**); 2 mm (**B**); 1.25 mm (**C**); 1 mm (**D**). Drawn by Hilde Orye.

##### Etymology.

Latin *piscator -oris*, fisherman; the species is used to impregnate fishing nets to attract fishes.

##### Distribution.

Endemic of SE DR. Congo (Haut-Katanga).

##### Habitat and ecology.

Shrub savannah, 890 m elev.

##### Additional specimens.

None, known only from the type specimen.

##### Note.

Vernacular name: lukakatjila (in kiluba).

#### ﻿ Coleus prittwitzii

Taxon classificationPlantaeLamialesLamiaceae

(Perkins) A.J.Paton, Phytokeys 129: 86. 2019.

AEC16F91-FAA5-5686-8DFA-B3041CB74087

≡ Pycnostachysprittwitzii Perkins, Notizbl. Bot. Gart. Berlin-Dahlem 8: 68. 1921. Type: Tanzania, Iringa District, Ndembera flood plain, near Gominyi, 2 Aug 1901, *G.von Prittwitz 28* (holotype B destroyed; isotype K [K000405965] fragment).

##### Description.

Paton et al. (2009: 398), Paton et al. (2013: 324), as *Pycnostachysprittwitzii* Perkins.

##### Distribution.

SW Tanzania to N Zambia.

##### Habitat and ecology.

Savannah on moist soil; ca. 1200 m elev.

##### Additional specimens.

Burundi, Mutara, Mosso, 12 Aug 1952, *G.Michel 3686* (BR, K); Kiofi, Mosso, 16 Sep 1952, *G.Michel 3907* (BR).

##### Note.

The two cited specimens differ from other materials of *C.prittwitzii* in having opposite (not ternate), broader, leaves and shorter inflorescence; they could represent a different taxon.

#### ﻿ Coleus pseudoschizophyllus

Taxon classificationPlantaeLamialesLamiaceae

Meerts & A.J.Paton sp. nov.

6AF4E0FB-4ECC-596E-BDAF-2435EFE4EAD8

urn:lsid:ipni.org:names:77347705-1

Fig. 16A–F

##### Type.

DR. Congo, Haut-Katanga, Marungu, Katomia, 18 Apr 1939, *P.J.J.Vanden Brande 55* (holotype BR [BR0000016832183]).

##### Diagnosis.

Closely related to *C.schizophyllus* on account of pinnatisect leaves and tuberous rootstock, differing by the conspicuously exserted sigmoid corolla tube, larger leaves, exserted stamens, tightly appressed indumentum and lack of glandular hairs in the inflorescence. It is also very closely related to *C.welwitschii*, differing by the jagged to pinnatisect leaves and rootstock with tubers.

##### Description.

Perennial herb, or suffrutex, 0.30–0.80 m high, from a thick rhizomatous rootstock, with fusiform tubers up to 5 cm long. Stem ascending to erect, the lower part woody, defoliated, purplish, strongly quadrangular, thinly puberulous with short adpressed retrorse hairs, upwards more densely puberulous and with red sessile glands, with a few long patent hairs at nodes, branched. Leaves opposite, petioled, often with fascicles of young leaves in the axils, ascending to spreading; blade ovate to trullate, 1.0–2.8 × 0.6–2.5 cm, apex acute, base more or less abruptly contracted and then attenuated into the petiole, margin markedly recurved, jagged to pinnatisect, with 3 to 6 lobes on either side, apical lobe narrowly oblong-triangular 3–5 mm long, upper surface dark green in herbarium, appressed pubescent, ca. 3 pairs of secondary veins, markedly impressed, lower surface markedly paler, pubescent on venation, this prominent, with dense red sessile glands; petiole 0.5–1.5 cm long, narrowly winged over the whole length, canaliculate, margin strongly recurved. Inflorescence lax, 8–10 cm long, 9–16 mm wide at anthesis (corolla excluded), with 12–15 verticils, spaced 6–10 mm, bracts ovate, cucullate, acuminate, 2–3 mm long, not forming a coma, ciliate, soon caducous, cymes sessile or with 1-mm long peduncle, of 5–12 flowers, cincinni 2–4 mm long, pedicels 2–4 mm, appressed pubescent, curved at tip, inserted very eccentrically in front of calyx upper lobe. Flower: calyx ca. 1.5 mm long at anthesis, pubescent, with red sessile glands, fruiting calyx 4–5 mm long, tubular, tube with 10 prominent ridges, throat truncate; posterior lip obovate, slightly recurved, very shortly decurrent, rounded to apiculate, ca. 2 mm long, lateral lobes oblong, ca. 2 mm long, rounded to truncate, median lobes of anterior lip fused into a 3–4 mm long linear lip, with teeth acute. Corolla white, 7–11 mm long, very shortly pubescent or puberulent on lobes, with red sessile glands, tube strongly sigmoid, 3–5 mm long, the sigmoid part long exserted, upper lip ca. 1 mm long, shallowly 4-lobed, much shorter than lower, lower lip ca. 4–5 mm long, 1.5–2 mm deep, somewhat upwardly curving, stamens exserted, filaments fused, anther ca. 0.5 mm long, style very shortly bifid, the branches often not divergent. Fruit: nutlets not observed.

**Figure 16.**
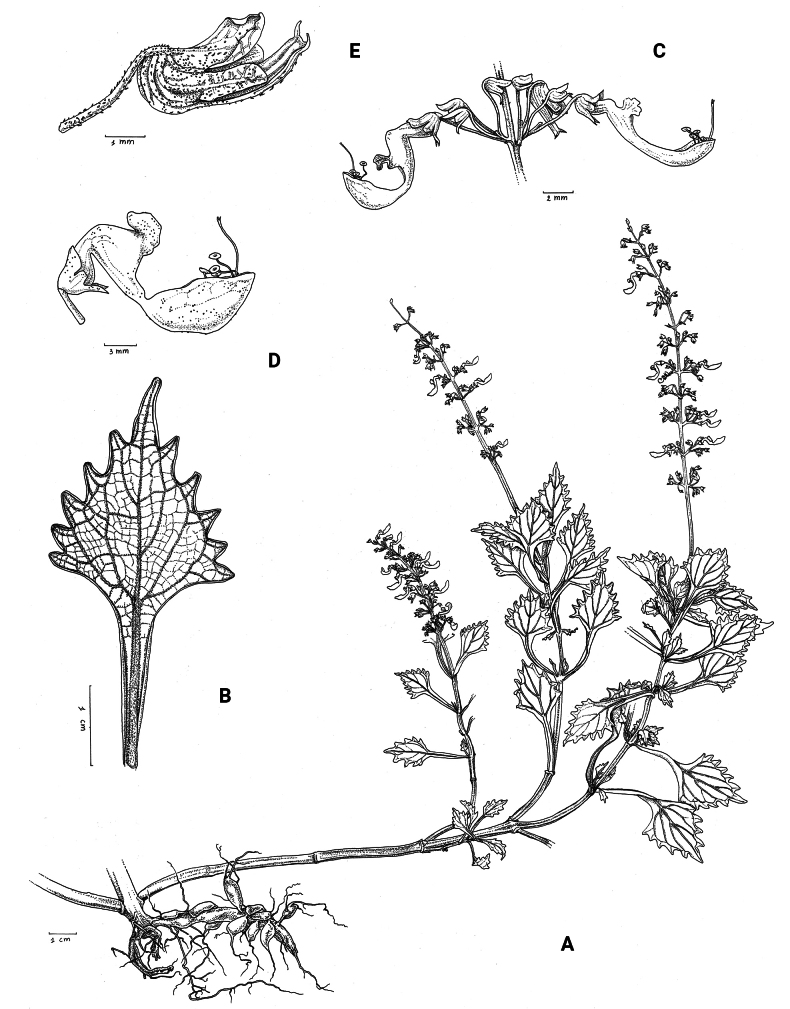
*Coleuspseudoschizophyllus* Meerts & A.J.Paton **A** habit **B** leaf **C** detail of inflorescence **D** flower **E** fruiting calyx **A** (except roots) **B–D***L.Dubois 1140***A** (roots only) **E***P.J.J.Vanden Brande 55*. Drawn by Hilde Orye. Scale bars: 1 cm (**A, B**); 2 mm (**C**); 3 mm (**D**); 1 mm (**E**).

##### Etymology.

The species superficially resembles *Coleusschizophyllus* (Baker) A.J.Paton on account of the deeply lobed leaves.

##### Distribution.

Endemic of SE DR. Congo (Haut-Katanga, Marungu Massif).

##### Habitat and ecology.

Rock crevices on mountains, scrub, steppic savannah on rocky soil (rhyolite); ca. 1600–2450 m elev.

##### Additional specimens.

DR. Congo, ***Haut-Katanga***, Marungu, Ndawa, 2200 m elev., Apr 1944, *L.Dubois 1140* (BR); Marungu Massif, Katomia, 18 Apr 1939, *P.J.J.Vanden Brande 67* (BR); Marungu, Katuba, broussailles sur affleurement de rhyolithes, 27 Jun 1957, *P.Duvigneaud 3744L* (BRLU); Marungu, Env. Kasiki, Mont Kilonge, 12 Jun 1969, *S.Lisowski, F.Malaisse, J.-J.Symoens 6518* (POZG); Marungu, 10 km NNE Kasiki, sommet Lusale, 2450 m elev., 26 Nov 1969, *S.Lisowski, F.Malaisse, J.-J.Symoens* 8460 & 8460a (POZG); Marungu, 7 km WSW de Luonde, 16 Feb 1970, *S.Lisowski, F.Malaisse, J.-J.Symoens 9768* (POZG); Marungu, Mont Zuiba, steppe, 21 Feb 1970, *S.Lisowski, F.Malaisse, J.-J.Symoens* 10483 (POZG).

#### ﻿ Coleus pseudospeciosus

Taxon classificationPlantaeLamialesLamiaceae

(Buscal. & Muschl.) A.J.Paton, Phytokeys 129: 87. 2019.

30D5ADC5-2283-57D5-96E8-1BEB2F88CAF0

≡ Pycnostachyspseudospeciosa Buscal. & Muschl., Bot. Jahrb. Syst. 49: 486. 1913. Type: Zambia, Lake Bangweulu, s.d, *E.D’Aosta 1002* (lectotype K [K000405996] fragment, designated by Ryding [2001]).
= Pycnostachysballotoides Perkins, Notizbl. Bot. Gart. Berlin-Dahlem 8: 72. 1921. Type: DR. Congo, Katanga, Mt Senga, May 1908 [“1906” in protologue], *T.Kassner 2930a* (holotype B destroyed).
= Pycnostachysmausaensis De Wild., Notes Fl. Katanga 7: 47. 1921. Type: DR. Congo, Katanga, Mt Senga, mt. slope, 31 May 1908, *T.Kassner* 2920a (holotype BR [BR0000013409814]; isotype K [K000405742]).

##### Description.

Paton et al. (2013: 326), as *Pycnostachyspseudospeciosa* Buscal. & Muschl.

##### Distribution.

S DR. Congo to N. Zambia.

##### Habitat and ecology.

Steppic savannah on seasonally set soil (dilungu), pond margin, more rarely dry woodland; 1400–1750 m elev.

##### Additional specimens.

DR. Congo, ***Haut-Katanga***, Au-delà de Kakera vers Baudouinville [Moba], 29 Jun 1957, *P.Duvigneaud 3773* (BRLU); Buyé Bula, affl. Muye, 30 Mar 1948, *G.F.de Witte 3595* (BR, WAG); Mont Senga, 31 May 1908, *T.Kassner 2920a* (K); Près de la rivière Mutungulu, 16 May 1971, *F.Malaisse 1357* (BR).

#### ﻿ Coleus repens

Taxon classificationPlantaeLamialesLamiaceae

Gürke, Bot. Jahrb. Syst. 19: 213. 1894.

43BF860A-DFC3-519A-B688-0EAACA2157AE

##### Type.

Cameroon, Mt Kupe, Nyasoso, nature trail above the Government High School, *B.Pollard 83* (neotype K [K000051084]; isoneotype KUPE, WAG, YA, designated by Pollard [2005]).

##### Description.

Paton (2022: 53).

##### Distribution.

W & WC Tropical Africa.

##### Habitat and ecology.

Rain forest; ca. 500 m elev.

##### Additional specimens.

DR. Congo, ***Forestier Central***, Katako Kombe, Jan 1910, *J.Claessens 406* (BR).

##### Note.

This species is known from a single collection in DR. Congo, ca. 1000 km east of nearest localities in the Republic of Congo; the materials from DR. Congo departs from typical materials in having lateral calyx teeth obtuse at tip, not acuminate.

#### ﻿ Coleus rhodesianus

Taxon classificationPlantaeLamialesLamiaceae

(N.E.Br.) A.J.Paton, Phytokeys 129: 88. 2019 (“ rhodesianum”).

B7CDB4F8-F386-5EFB-880C-C43816A1AB52

≡ Englerastrumrhodesianum N.E.Br., Bull. Misc. Inform. Kew 1922: 31. 1922. Type: Zambia, Mumbwai, *M.A.Macaulay 637* (syntype K), & Zambia, Livingstone, *F.A.Rogers 7205* (syntype, not seen).
= Englerastrumschweinfurthii Briq., Bot. Jahrb. Syst. 19: 178. 1894, non Coleusschweinfurthii Vatke. Type: South Sudan (“Ghasallquellengebiet, Lande der Bongo”), Addai, 19 Oct 1869, *G.Schweinfurth 2532* (K, P, PRE, W).
= Plectranthusdjalonensis (A.Chev.) A.J.Paton, Fl. Trop. E. Afr., Lamiac.: 286. 2009. Type: Guinea, sur les plateau ferrugineux arides entre Timbo et Kouria, Sept 1907, *A.Chevalier s.n.* (holotype P [P00466389]; isotype K).

##### Description.

Paton et al. (2009: 286), Paton et al. (2013: 236), as *Plectranthusdjalonensis* (A.Chev.) A.J.Paton.

##### Distribution.

Tropical Africa to Caprivi Strip.

##### Habitat and ecology.

River-bank, ruderal, open vegetation on wet soil, savannah, palm oil plantation, mostly on wet soil; forests, rarely on copper rich soil; very broad ecological amplitude in terms of light and soil moisture; 100–1750 m elev.

##### Additional specimens.

DR. Congo, ***Mayombe***, Lukula, 1 Sep 1913, *J.Bequaert 671* (BR); Luki, 3 Apr 1947, *R.Devred 3363* (BR, POZG); ***Bas-Congo***, Kisantu, 4 Mar 1959, *L.Pauwels 2005* (BR, WAG); Dolo [Ndolo], Jun 1899, *R.Schlechter 12490* (AMD, BM, BR, E, G, L, P); ***Kasaï***, Lusambo, 15 Mar 1939, *P.Casier 54 & 56* (BR); Suka, Jul 1975, *M.Dujardin 401* (BR); ***Bas-Katanga***, Mwene-Ditu, Kele, 13 May 1957, *L.Liben 2946* (BR); Gandajika, 18 Mar 1954, *S.Risopoulos 196* (BR); ***Forestier Central***, Yangambi, Ile Tukutu, 10 Jul 1963, *D.Bolema 1178* (BR); Kisangani, Ile Kongolo, 3 Mar 1978, *J.Lejoly 2747* (BR, BRLU, K, WAG); Yoambole, entre Lileko et Basoko, 28 Sep 1938, *J.Louis 11437* (BR); Bikoro, Gombe, 20 May 1959, *L.Toka 79* (BR, WAG); ***Ubangi-Uele***, Doruma, Oct 1936, *A.M.De Graer 754* (BR, K, WAG); Camp Garamba, 10 Oct 1955, *M.Micha 296* (BR, WAG); ***Lacs Edouard et Kivu***, Ruzizi, May 1967, *J.Loumaye s.n*. (BR); ***Haut-Katanga***, Upemba, riv. Manda, 20 Apr 1948, *G.F.de Witte 3736* (BR); Entre Mulubi et Kashika, 29 Apr 1926, *J.Lebrun 2119* (BR); Kwatebala, 24 Apr 2006, *F.Malaisse, E.Kisimba, L.Saad 21* (BR).

Rwanda, Route Nyamasheke-Kibuye, km 19, 29 Mar 1972, *G.Bouxin 1527* (BR); Mushao, May 1929, *H.Humbert 8460* (BR); Rwinkwavu, Plaine de Matinza, 21 May 1969, *G.Bouxin & M.Radoux 450* (BR).

Burundi, Aérodrome de Bujumbura, 16 Mar 1967, *J.Lewalle 1680* (BR, MO); Kabuyekere, 5 Jun 1980, *M.Reekmans 9282* (BR, MO, US); Kigwena, 13 May 1982, *M.Reekmans 11199* (BR, MO, WAG).

#### ﻿ Coleus rotundifolius

Taxon classificationPlantaeLamialesLamiaceae

(Poir.) A.Chev. & Perrot, Veg. Ut. Afr. Trop. Franç. 1: 101. 1905.

B6DCF94C-CE36-502C-A1F9-D0A9944B3D01

≡ Germanearotundifolia Poir. in J.B.A.M.de Lamarck, Encycl. 2: 763. 1788.
≡ Plectranthusrotundifolius (Poir.) Spreng., Syst. Veg., ed. 16. 2: 690 (1825). Type: Mauritius (“Isle de France”), *P.Commerson s.n.* (holotype P [P00152706]; isotype FI).

##### Description.

Paton et al. (2009: 330); Paton et al. (2013: 269), as *Plectranthusrotundifolius* (Poir.) Spreng.

##### Habitat and ecology.

Fallow field; ca. 400 m; in Africa, always in cultivation or as a relic of old cultivation.

##### Additional specimens.

DR. Congo, ***Bas-Congo***, Kasangulu, 22 Apr 1960, *P.Compère 1991* (BR).

Rwanda, Nyabarsingo, *Simpson s.n*. (TCD, not seen, cited in Suddee et al. 2004).

##### Notes.

1. New species record for DR. Congo.

2. This species belongs in the difficult group of *C.bojeri-C.welwitschii*, in which identification requires carefully collected underground organs. The collecting notes on the label of *P.Compère 1991* mention the presence of tubers, but these have not been collected. Apart from tubers, the species differs from *C.bojeri* in having somewhat thicker and more pubescent leaves.

3. See note under *C.welwitschii*.

#### ﻿ Coleus ruandensis

Taxon classificationPlantaeLamialesLamiaceae

(De Wild.) A.J.Paton, Phytokeys 129: 90. 2019.

514F6BE5-6194-5823-A3A2-0683586DFB8D

≡ Pycnostachysruandensis De Wild., Pl. Bequaert. 4: 401. 1928. Type: Rwanda, between Kirinda and Lubengera, 6 Jun 1926. *W.Robyns 2449* (holotype BR [BR0000008910424], [BR0000008909770]; isotype K [K000405731], P [P00541261]).

##### Description.

Paton et al. (2009: 389), Paton et al. (2013: 318), as *Pycnostachysruandensis* De Wild.

##### Distribution.

Uganda, Burundi, Rwanda, DR. Congo and Malawi.

##### Habitat and ecology.

Rainforest, riparian forest, fallow field, savannah; 900–2460 m elev.

##### Additional specimens.

DR. Congo, ***Lacs Edouard et Kivu***, Kabare, Ludaha, s.d., *Gilon 3*3 (BR); Nya Kaziba, 17 Apr 1952, *J.F.Laurent 462* (BR); ***Haut-Katanga***, Upemba, Mbuye-bala, 15 Apr 1948, *G.F.de Witte 3748* (BR, K, WAG); Marungu, Kaboto, Apr 1944, *L.Dubois 1126* (BR, WAG); Marungu, 3 km W de Kasiki, 12 Jun 1969, *S.Lisowski, F.Malaisse, J.-J.Symoens 6147* (POZG).

Rwanda, Murambi-Kivumu, 14 May 1983, *F.-X.Ayobangira 1618* (BR).

Burundi, Nyakakaro, 30 Jun 1993, *C.Carème s.n.* (BR); Mwaro, Mont Mugero, 27 Apr 1966, *J.Lewalle 733* (BR, LSHI, MO); Bujumbura, 19 May 1971, *J.Lewalle 5733* (BR); Kisozi, 3 Jun 1934, *J.B.H.Lejeune 86* (BR, K, WAG); Butare, Mosso, Pont de Musasa, 13 May 1981, *M.Reekmans 10273* (BR, US); Kiganda, 24 May 1981, *M.Reekmans 10373* (BR, MO, WAG).

#### ﻿ Coleus ruziziensis

Taxon classificationPlantaeLamialesLamiaceae

Meerts & A.J.Paton sp. nov.

1B510F2A-8C7A-5D63-8CC9-0A91B6FE37CF

urn:lsid:ipni.org:names:77347706-1

Fig. 17A–F

##### Type.

Burundi. Usumbura [Bujumbura], 800 m elev., Dec 1934. *A.Becquet 834* (holotype BR [BR0000016830684]; isotype K).

##### Diagnosis.

Differing from all other *Coleus* species by the following combination of traits: leaves grouped in the lower third of the shoot, almost forming a rosette, roots produced into fusiform tubers, cymes (3–)4-flowered.

##### Description.

Perennial herb, somewhat succulent, aromatic (scent reminiscent of *Lavandula*), 0.2–0.5 m high; roots dilated into fusiform tubers up to 40 × 8 mm. Stem erect, leafy in the lower third, quadrangular, sulcate, with patent eglandular hairs and shorter glandular hairs, these denser in the inflorescence, simple or sparingly branched in the inflorescence. Leaves present at flowering, at three to four nodes in the lower part of the shoot, with short internodes and often almost rosulate, spreading, with or without fascicles of small leaves in the axils; blade ovate-elliptic to elliptic or obovate-elliptic, (3–)6–13 × (1–)2–7 cm, base obtusely cuneate to attenuate, apex obtuse to rounded, margin undulate to shallowly crenate, ca. 6 secondary veins on either side diverging at an acute angle, main veins flattened on lower surface, upper surface velvety pubescent, lower surface tomentose to villous and with red sessile glands; petiole canaliculate, 2–4(–5) cm long, long pubescent to villous. Inflorescence terminal, lax, (9–)12–28 cm from lowermost node, simple or branched at the lowermost node, verticils laxly disposed, spaced 1–2(–4) cm, cymes sessile, 3–4-flowered; bracts broadly ovate, rounded at tip, ca. 2 × 2 mm, soon caducous, not forming a coma; pedicels 1–2 mm long, elongating to 4–5 mm in fruit, ascending to patent, curved near apex, shortly pubescent and with sparse glandular hairs, eccentrically inserted on calyx in front of upper lobe. Flower: calyx campanulate, at anthesis ca. 2 mm long, in fruit 4–5 mm, pubescent with broad-based short hairs on the veins and with sparse orange or red sessile glands, teeth margin ciliolate, inner side papillate, with pedicel attached asymmetrically in front of upper lip, throat truncate to slightly oblique, posterior lip broadly obovate to almost rounded, 2–3 × 2–3 mm, apex rounded to apiculate or emarginate, curving upwards, not or shortly decurrent, lateral lobes midway between upper lip and median lobes of lower lip, triangular, ca. 1.2 mm long, margin narrowly recurved, median lobes narrowly triangular, ca. 3 mm long, curving upwards in fruit; corolla pale mauve to blue, (10–)12–15 mm long, sparsely pubescent, obliquely hanging, tube almost straight to slightly sigmoid, subglabrous, 5–6 mm long, progressively dilated near throat, upper lip 2–3 mm long, shortly pubescent, lower lip shortly pubescent, 5–7 mm long, ca. 2 mm deep, enclosing stamens, anthers ca. 0.5 mm long, round; style bifid. Nutlets brown, shiny, rounded, slightly compressed, ca. 1 mm diam.

**Figure 17.**
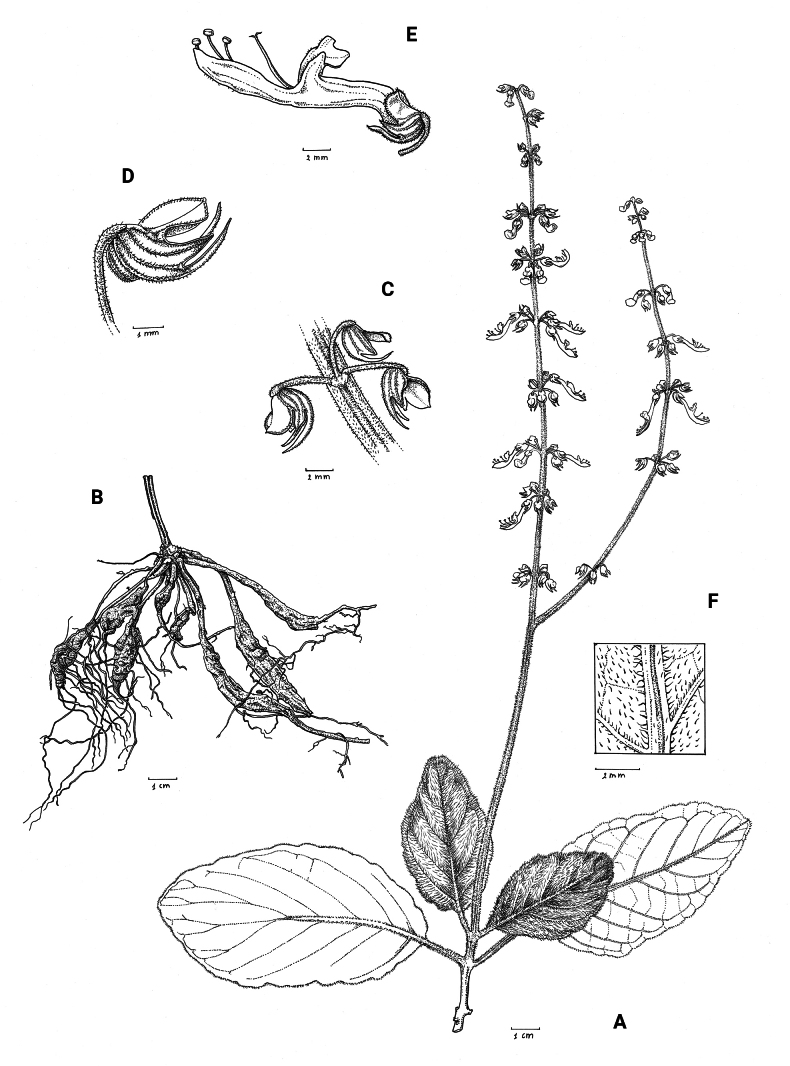
*Coleusruziziensis* Meerts & A.J.Paton **A** habit **B** roots with tubers **C** detail of inflorescence **D** fruiting calyx **E** flower **F** detail of pubescence of leaf undersurface **A***A.Becquet 834*, *M.Reekmans 149*, *H.Humbert 7314***B, C, D***A.Becquet 834***E***J.Lewalle 2401***F***J.Lewalle 2401*). Drawn by Hilde Orye. Scale bars: 1 cm (**A, B**); 2 mm (**C, E, F**); 1 mm (**D**).

##### Etymology.

All collections of the species originate from the Ruzizi River plain.

##### Distribution.

E DR. Congo (Kivu) and W Burundi; endemic of the Ruzizi River plain.

##### Habitat and ecology.

Savannah, steppe, often on rocky or sandy soil; association à *Loudetiasimplex* et *Crabbaeavelutina*; 800–950 m elev.; flowers in the first half of the rain season.

##### Additional specimens.

DR. Congo, ***Lacs Edouard et Kivu***, Plaine de la Ruzizi, route Costermansville-Uvira, 20 Feb 1950, *R.Germain 6159* (BR); Plaine de la Ruzizi, savane à *Heteropogon-Chloris*, 24 Feb 1950, *R.Germain 6234* (BR); Plaine de la Ruzizi, Nyakikumba, env. 900 m elev., savane arborée brûlée, Nov. 1948, *F.L.Hendrickx 5678* (BR); Plaine de la Ruzizi, ca. 800 m elev., env. d’Uvira, Jan. 1929, *H.Humbert 7314* (BR, P); Matiaso, 28 Dec 1950, *P.Liégeois 347* (BR).

Burundi, Ferme de la Randa, 03°07'S, 29°22'E, 950 m elev., prairie en pente faible, pâturée, 14 Nov 1965, *J.Lewalle 16* (BR, MO); Ruzizi, km. 35, prairie sableuse, 25 Nov. 1967, *J.Lewalle 2401* (BR); Plaine de la Ruzizi, Ruziba, 900 m elev., savane arbustive, 5 Dec1969, *J.Lewalle 4152* (BR); Bubanza, Cibitoke, 900 m elev., steppe rocheuse, 21 Feb 1971, *M.Reekmans 149* (BR); Bubanza, Cibitoke, steppe rocheuse, 21 Nov 1971, *M.Reekmans 1183* (BR); Bujumbura, Randa, savane, 900 m elev., 30 Jan 1972, *M.Reekmans 1499* (BR); Bubanza, Kagongwe, vallée Katunguru, 26 Nov 1972, *M.Reekmans 2136* (BR, K).

##### Notes.

1. *C.ruziziensis* has a very original combination of characters, i.e. fusiform tubers, a rosette of ovate leaves and 4-flowered cymes. Calyx characters are close to *C.hadiensis*. It superficially resembles *Orthosiphonallenii* in having a rosette and tubers.

2. Vernacular name: mwendekere (in kirundi); katjudju, mutuzo (in bashi).

#### ﻿ Coleus schliebenii

Taxon classificationPlantaeLamialesLamiaceae

(Mildbr.) A.J.Paton, Phytokeys 129: 92. 2019.

CAED1199-60F5-565B-98FE-43F378078BB6

≡ Pycnostachysschliebenii Mildbr., Notizbl. Bot. Gart. Berlin-Dahlem 11: 405. 1932. Type: Tanzania, Njombe District: Lupembe, stromgebiet des oberen Ruhudje, landschaft Lupenbe, nördlich des Flusses, Apr 1931, *H.J.Schlieben 713* (holotype B destroyed: isotype BM [BM000884009], BR [BR0000006410650], G [G00435269], K [K000405738] fragment, MA [MA384632], P [P00541260]).

##### Description.

Paton et al. (2009: 410), Paton et al. (2013: 330), as *Pycnostachysschliebenii* Mildbr.

##### Distribution.

Tanzania to Zambia and E DR. Congo.

##### Habitat and ecology.

Mountain forest with *Arundinaria*, *Erica* scrub, river bank, more rarely wooded savannah; in Burundi also in savannah and fallow fields, (950–)1750–2500 m elev.

##### Additional specimens.

DR. Congo, ***Lacs Edouard et Kivu***, Katana, 10 Jul 1959, *Cambridge Congo Expedition 80* (BR, US); Chaîne des Mitumba, W Lac Edouard, piste Mangi-Kipesa, 19 Jan 1956, *J.de Wilde 623* (BR); Entre Kasindi et Lubango, Dec 1931, *J.Lebrun 4690* (BR); Kalehe, Mont Kahuzi, 7 Jul 1971, *J.Ntakiyimana 92* (BR); ***Haut-Katanga***, Upemba, galerie de la Kalumengongo, 18 Apr 1947, *G.F.de Witte 2518* (BR); Marungu, env. Kasiki, fourré de la source de la rivière Lunanga, 12 Jun 1969, *S.Lisowski, F.Malaisse, J.-J.Symoens 6429* (POZG); Upemba, tête de source de la riv. Kadidika, près de la piste Lusinga-Mitwaba, 11 May 1949, *van Meel* in *de Witte 6321* (BR).

Burundi, Mont Manga, 21 Apr 1982, Missumba, 4 Jun 1970, *J.Lewalle 4676* (BR); Rushubi, Mont Bona, 26 Apr 1977, *M.Reekmans 6025* (BR, WAG); Kitwe Rumonge, 12 May 1977, *M.Reekmans 6211* (BR, WAG); Kabuyekere, 5 Jun 1980, *M.Reekmans 9287* (MO, WAG); Bujumbura, Mont Manga, 21 Apr 1982, *M.Reekmans 1099*4 (BR, WAG); Bukemba-Muzye, 12 May 1981, *M.Reekmans 10220* (BR, MO, WAG).

##### Notes.

1. This species is variable in Burundi; a particular morphotype occurring in savannah has long ciliate bracts, calyx tube with a ring of hairs inside the throat, short inflorescence (1 cm at anthesis) and short calyx teeth (e.g. *J.Lewalle 1912, G.Michel 2408, 2715, 2956, M.Reekmans 486, 5074, 6211, 7035, 9287, 10220, J.Saintenoy 158*); such plants were identified *in schedis* as “*P.bruceae* Gatheri” (unpublished designation); further work is needed to assess their taxonomic status.

2. In the Marungu Massif (Haut-Katanga), the species is represented by a large-flowered phenotype (corolla up to 20 mm long) e.g. *S.Lisowski, F.Malaisse, J.-J.Symoens 6428* (POZG) which could deserve taxonomic recognition; further work is needed on this polymorphic species.

3. The specimens *J.F.Laurent 489* and *Bytebier & Luke 3128* (BR, EA) could represent *Coleuslivingstonei* A.J.Paton, a species not yet recorded in Central Africa; however, the materials are poor and the presence of this species needs confirmation.

#### ﻿ Coleus scruposus

Taxon classificationPlantaeLamialesLamiaceae

A.J.Paton, Phytokeys 129: 92. 2019.

A19041EC-F93B-53AF-9AE4-718F18469FE7

≡ Pycnostachyskassneri De Wild., Contr. Fl. Katanga: 172. & Ann. Soc. Sci. Bruxelles 41(2): 54. 1921., non Coleuskassneri Robyns & Lebrun.
= Pycnostachyscongensis Gürke, Bull. Herb. Boissier 4: 819. 1896. Type: DR. Congo, Lusambo, 1891, *G.Descamps 27* (lectotype Z [Z-000021015], **designated here**).
≡ Coleuscongensis (Gürke) A.J.Paton, Phytokeys 129: 35. 2019.

##### Type.

DR. Congo, Katanga, West Kundelungu, 17 May 1908, *T.Kassner 2794* (holotype BR [BR0000009824614]; isotype BM [BM000838018], HBG, K [K000405735]).

##### Description.

Paton et al. (2009: 390), Paton et al. (2013: 318), as *Pycnostachyskassneri* De Wild.

##### Distribution.

Tanzania to Zambia.

##### Habitat and еcology.

Savannah, riparian forest, scree, dry woodland; 950–1650 m elev.

##### Additional specimens.

DR. Congo, ***Kasaï***, Sandoa, près de Kambungu, 23 Apr 1959, S.Risopoulos 970 (BR, K, WAG); ***Bas-Katanga***, Samba, 1891, *G.Descamps 28* (BR); Kamina, Lovoi, Mar 1932, *P.Quarré 2971* (BR); ***Haut-Katanga***, Upemba, Mont Muye, 3 May 1948, *G.F.de Witte 3774* (BR); Vallée de la Kalumengongo, près des chutes, 12 Apr 1949, *G.F.de Witte 6071* (BR, K, WAG); Mugila, 23 May 1908, *T.Kassner 2967* (K); Kundelungu, 17 km NW Sampwe, 24 Apr 1970, *S.Lisowski, F.Malaisse, J.-J.Symoens 11072* (BR, POZG); Route Kolwezi-Kamina, km 75, 5 Apr 1948, *A.Schmitz 1780* (BR).

##### Notes.

1. Calyx teeth length is variable; specimens from DR. Congo and Zambia with very short calyx teeth (< 2 mm) also tend to have calyx throat that is tomentose inside (e.g. *A.Schmitz 1780*, *J.-J.Symoens 10239*). Further work is needed to decide if this represents a distinct taxon or just one extreme of a continuous variation.

2. Lectotypification of *Pycnostachyscongensis* Gürke. Gürke (1896) cited two syntypes of *Pycnostachyscongensis* Gürke (*G.Descamps 27* (syntype B destroyed; isosyntype Z), DR. Congo, Lusambo & *G.Descamps 28* (syntype B destroyed; isotype BR), DR. Congo, Samba, 1891. We select *G.Descamps 27* (Z) as the lectotype because it comprises a whole plant.

#### ﻿ Coleus seretii

Taxon classificationPlantaeLamialesLamiaceae

De Wild., Bol. Soc. Ibér. Ci. Nat. 19: 122. 1920.

BFEF9835-8D97-5D16-8FE5-FC4933C27E23

≡ Plectranthusseretii (De Wild.) Vollesen, Opera Bot. 59: 84. 1980. Type: DR. Congo, route Buta–Bima (Bali), 13 Oct 1905, *F.Seret 68* (lectotype BR [BR0000006262600], **designated here**).

##### Description.

Paton et al. (2009: 313), as *Plectranthusseretii* (De Wild.) Vollesen.

##### Distribution.

Ethiopia, DR. Congo, Tanzania, NW. Madagascar.

##### Habitat and еcology.

Secondary forest, road verge; 450–600 m elev.

##### Additional specimens.

DR. Congo, ***Bas-Katanga***, Kasongo, Mobanga, 18 Jun 1952, *R.Germain 7695* (BR); ***Forestier Central***, Route Benalia-Buta, 1 Nov 1945, *R.Germain 4267* (BR); Route Buta-Banalia, km 38, 23 May 1976, *S.Lisowski 42922* (POZG); Entre Buta et Lekada, 9 Jan 1926, *W.Robyns 1320* (BR).

##### Note.

Lectotypification of *Coleusseretii* De Wild. De Wildeman (1920) cites two syntypes (*F.Seret 68 & 420* (erroneously cited as “120” in the protologue), both matching the protologue; *F.Seret 68* (sheet [BR0000006262600] is selected here as the lectotype because it has better preserved leaves. Remaining syntype: *F.Seret 420* (syntype BR; isosyntype K), Suronga Forest, 26 Dec 1905.

#### ﻿ Coleus shirensis

Taxon classificationPlantaeLamialesLamiaceae

Gürke, Bot. Jahrb. Syst. 19: 216. 1894.

94284EB1-C581-50AF-96A8-792D3DB21BC9

≡ Plectranthusshirensis (Gürke) A.J.Paton, SABONET Rep. Ser. 31: 189. 2005. Type: Malawi, 1891, *J.Buchanan 376* (lectotype K [K000070492]; isolectotype E [E00193509], designated by Mathew [1976]).

##### Description.

Paton et al. (2009: 336), Paton et al. (2013: 272), as *Plectranthusshirensis* (Gürke) A.J.Paton.

##### Distribution.

SW & S Tanzania to S Tropical Africa.

##### Habitat and еcology.

Riparian forest, swamp savannah, forest margins; 1000–1800 m elev.

##### Additional specimens.

DR. Congo, ***Bas-Congo***, Kinkosi, 26 Feb 1959, *L.Pauwels 1600* (BR); ***Kasaï***, Entre Kwango-Lufuna, 4 Aug 1944, *R.Germain 2643* (BR); Kwango, 21 Jul 1955, *R.Devred 2290* (BR); ***Bas-Katanga***, Gandajika, 20 Jun 1951, *Chalon 326* (BR); Kindele, Sep 1951, *R.Desenfans 2013* (BR, BRLU); 10 km SW Gandajika, 9 Aug 1945, *F.Luxen 534* (BR); Lovoi, Kamina, Mar 1932, *P.Quarré 2929* (BR); ***Haut-Katanga***, Mulumbi, riv. Dona, 30 Aug 1953, *R.Desenfans 3731* (BRLU); 14 km N de Mitwaba, 16 Jan 1960, *P.Duvigneaud 5077Co* (BRLU); Kundelungu, 6 km NNW poste Katshupa, 29 Jul 1966, *F.Malaisse 4213a* (BR; LSHI); Kisanga, Aug 1933, *P.Quarré 3466* (BR); Upemba, Lusinga, route de Mitwaba, 14 Sep 1948, *W.Robyns 3590* (BR); Keyberg, 24 Jun 1947, *A.Schmitz 732* (BR).

##### Note.

At the northern limit of the distribution range, some specimens are unusual in lacking long hairs on pedicel and calyx (*Herman 2308*, *M.Schaijes 1885, P.Quarré 2929*).

#### ﻿ Coleus sphaerocephalus

Taxon classificationPlantaeLamialesLamiaceae

(Baker) A.J.Paton, Phytokeys 129: 98. 2019.

19B83560-41AC-5A1B-B7EB-C9F106EAC1C0

≡ Pycnostachyssphaerocephala Baker, Bull. Misc. Inform. Kew 1898: 162. 1898. Type: Malawi, Nyika Plateau, Jul 1896, *A.Whyte 139* (holotype K [K000406000]).

##### Description.

Paton et al. (2009: 402), Paton et al. (2013: 327), as *Pycnostachyssphaerocephala* Baker.

##### Distribution.

Tanzania to Zambia.

##### Habitat and еcology.

Near springs, ca. 2200 m elev.

##### Additional specimens.

DR. Congo, ***Haut-Katanga***, Marungu, Musipi, Apr 1945, *L.Dubois 1402* (BR); Mont Mugila, 23 May 1908, *T.Kassner 2991a* (HBG, K).

#### ﻿ Coleus stachyoides

Taxon classificationPlantaeLamialesLamiaceae

(Oliv.) E.A.Bruce, Bull. Misc. Inform. Kew 1934: 306. 1934.

D9EB21AD-4D03-5710-99B0-B71FBD80F2E1

≡ Plectranthusstachyoides Oliv., Trans. Linn. Soc. London 29: 136. 1875. Type: Uganda, West Nile District: Madi, Dec 1862, *J.A.Grant 732* (holotype K [K000431982]).
= Plectranthuscylindrostachys Robyns & Lebrun, Rev. Zool. Bot. Africaines 16: 356. 1928. Type: Burundi, Irubura, Akanguru Valley, 31 May 1926, *W.Robyns 2403* (holotype BR [BR0000006263294]; isotype K [K000431886]).

##### Description.

Paton et al. (2009: 323), as *Plectranthusstachyoides* Oliv.

##### Distribution.

Central African Rep. to E Tropical Africa.

##### Habitat and еcology.

*Hyparrhenia-Loudetia* savannah, wooded savannah, sclerophyllous scrub, rock crevices; 800–3045 m elev.

##### Additional specimens.

DR. Congo, ***Bas-Katanga***, Kasengi, May 1947, *W.Mullenders 2405* (BR); Kisamba, Jan 1931, *P.Quarré 2377* (BR); ***Lacs Edouard et Kivu***, Ruzizi, Tsamate, Apr 1950, *R.Germain 6458* (BR); Mont Muhi, 31 Jul 1955, *U.Kinet 93* (BR); ***Haut-Katanga***, Kando, Mar-Apr 1931, *G.F.de Witte 204* (BR); Marungu, Kasiki, Mont Kilonge, 12 Jun 1969, *S.Lisowski, F.Malaisse, J.-J.Symoens 6388* (POZG); Muhila, Mont Mwango, 12 May 1971, *S.Lisowski 23526* (POZG).

Rwanda, S Kagera, Rwinkwavu, 13 Apr 1966, *J.Lewalle 686* (BR, MO); Rubona, 25 Apr 1958, *G.Michel 5311* (BR, MO); Kagera, colline Rwanyerajana, 5 Apr 1958, *G.Troupin 6833* (BR, LWI); Kibungu, Parc National Kagera, colline Gwenjange, 17 Jun 1958, *G.Troupin 7470* (BR, WAG); Kibungo, Mbuye, 28 Jun 1978, *G.Troupin 16166* (BR). BURUNDI, Gitega, route vers Karuzi, 27 Apr 1971, *J.Lewalle 5793* (BR, WAG); Kininya Mosso, 12 Jun 1952, *G.Michel 2785* (BR); Bubanza, Rugazi, 3 May 1981, *M.Reekmans 10091* (BR, WAG); Bukemba, Muzye, 12 May 1981, *M.Reekmans 10232* (WAG, MO, BR).

#### ﻿ Coleus stenostachys

Taxon classificationPlantaeLamialesLamiaceae

(Baker) A.J.Paton & Phillipson, Phytokeys 129: 100. 2019.

9342D3DB-6E0E-5587-9FA4-FE9554FBC500

≡ Pycnostachysstenostachys Baker in D.Oliver & auct. suc. (eds.), Fl. Trop. Afr. 5: 380. 1900. Type: Uganda, Bunyoro District: sides of Nile, Nov 1862, *J.A.Grant* in *J.H.Speke & J.A.Grant s.n.* (holotype K [K000405970]).
= Pycnostachyscoerulea Hook. Exot. Fl. 3: t. 202. 1826, non Coleuscoeruleus Gürke. Type: Madagascar, cultivated in Kew from seeds sent by *W.Bojer & Helsinger* (holotype K [K000406006]; isotype M [M0104752]).
= Pycnostachysbrevipetiolata De Wild., Pl. Bequaert. 4: 394. 1928. Type: DR. Congo, Kivu, rivière Rutshuru, Kaitafu, 4 Oct 1914, *J.Bequaert 5972* (holotype BR [BR0000008910363], [BR0000008909718]).

##### Description.

Paton et al. (2009: 393), Paton et al. (2013: 321), as *Pycnostachyscoerulea* Hook.

##### Distribution.

Ethiopia to South Africa, Madagascar.

##### Habitat and еcology.

River banks, lake shores, Papyrus marshland and swamps, *Pennisetum-Phragmites* savannah on moist soil; 900–1900 m elev.

##### Additional specimens.

DR. Congo, ***Lac Albert***, Nioka, 5 May 1952, *L.Liben 245* (BR, K); Nioka, rivière Duda, 22 Oct 1947, *A.Taton 666* (BR); ***Lacs Edouard et Kivu***, Kaitafu, rivière Rutshuru, 4 Oct 1914, *J.Bequaert 5972* (BR, K fragment); Lac Magera, 1 Mar 1934, *G.F.de Witte 1416* (BR); Lushadu, bord du Lac Kivu, 10 Jun 1960, *F.L.Hendrickx 7981* (BR, BRLU); Nyamunyunye, 22 Sep 1952, *R.Pierlot 391* (BR); Lac Lukulu, 22 Jul 1953, *D.van der Ben 654* (BR); ***Haut-Katanga***, Kapumfi, rive du Lac Moero, 31 Dec 1965, *J.-J.Symoens 12003* (BR, LSHI).

Rwanda, Lac Bulera, Butaro, 17 Feb 1972, *P.Auquier 2578* (BR); Butare, Jun 1933, *A.Becquet 714* (BR, WAG); Environs de Butare, rivière Nyamogari, 3 May 1957, *D.van der Ben 1547* (BR); Rubona, 16 Feb 1960, *G.Michel 6387* (BR); Akagera, marais Kajumbura, 30 Mar 1973, *G.Troupin 14897* (BR).

Burundi, Kanyanya, Lac Rwihinda, 18 Apr 1971, *J.Lewalle 5547* (BR); Gitwenge, 17 May 1978, *M.Reekmans 7056* (LG, WAG).

#### ﻿ Coleus stuhlmannii

Taxon classificationPlantaeLamialesLamiaceae

(Gürke) A.J.Paton, Phytokeys 129: 101. 2019.

506FED81-020C-56F8-8F36-D49AEE6BDD78

≡ Pycnostachysstuhlmannii Gürke in H.G.A.Engler, Pflanzenw. Ost-Afrikas, C: 345. 1895. Type: Tanzania, Bukoba District: Karagwe, Ngaramo, 6 Feb 1891, *F.Stuhlmann 1630* (holotype B destroyed; isotype K [K000405975] fragment).
= Pycnostachysbequaertii De Wild., Contr. Fl. Katanga: 171. & Ann. Soc. Sci. Bruxelles 41(2): 52. 1921. Type: DR. Congo, Katanga, Shinsenda, *J.Bequaert 425* (lectotype BR [BR0000008910103]; designated by Bramley in Paton et al. [2009]).
= Pycnostachyslongifolia De Wild., Contr. Fl. Katanga: 172. Ann. Soc. Sci. Bruxelles 41(2): 55. 1921. Type: DR. Congo, Katanga, Welgelegen, *J Bequaert 562* (holotype BR [BR0000008909787]; isotype K fragment).

##### Description.

Paton et al. (2009: 392), Paton et al. (2013: 319), as *Pycnostachysstuhlmannii* Gürke.

##### Distribution.

Kenya to S. Tropical Africa.

##### Habitat and еcology.

Marshland, dilungu savannah on moist soil, dambos, on organic soil; 850–1300 m elev.

##### Additional specimens.

DR. Congo, ***Haut-Katanga***, Shinsenda, 8 May 1912, *J.Bequaert 425* (BR, K fragment); Keyberg, 17 Apr 1957, *E.Detilleux 831* (BR); Dikuluwe, 10 May 1957, *P.Duvigneaud 3118P4* (BRLU), 1.5 km E of Kabiashia, dembo Kandale, 23 May 1969, *F.Malaisse 6435* (BR, LSHI, P); Route Lubumbashi-Kasenga, 2 km après Kumanua, 25 May 1985, *F.Malaisse & Goetghebeur 1144* (BR, K, P); Kipila, May 1929, *P.Quarré 1693* (BR, K, P); Env. Lubumbashi, May 1934, *H.Humbert 15906* (BR, P); Kipopo, 17 Apr 1962, *A.Schmitz 7700* (BR, K); Tumbwe, dembo de la Kasompa, 30 Apr 1960, *J.-J.Symoens 7608* (BR, LSHI).

BURUNDI, Lac Nyanza, 27 Jun 1971, *J.Lewalle 6050* (BR, K); Kinyinya Mosso, 5 Jun 1981, *M.Reekmans 10532* (BR, US, WAG).

#### ﻿ Coleus succulentus

Taxon classificationPlantaeLamialesLamiaceae

Pax, Bot. Jahrb. Syst. 39: 646. 1907.

672E5FC3-C323-5CD1-85BB-FD339462A4D2

= Plectranthuspseudomarrubioides R.H.Willemse, Kew Bull. 40: 93. 1985. Type: Ethiopia, Debre Libanos, 2 Nov 1965, *W.J.J.O.de Wilde & de Wilde-Duyfjes 8656* (holotype WAG [WAG0001746]; isotype BR [BR0000005232543]).

##### Type.

Ethiopia, Zuquala Mt., *O.Ryding & C.Puff 1657* (neotype UPS [V-057092]; isoneotype ETH, designated by Ryding [2000]).

##### Description.

Paton et al. (2009: 320), as *Plectranthuspseudomarrubioides* R.H.Willemse.

##### Distribution.

Ethiopia to N. Tanzania, Arabian Peninsula.

##### Habitat and ecology.

Saxicolous, near riparian forest, ca. 900 m elev.

##### Additional specimens.

DR. Congo, ***Haut-Katanga***, Kundelungu, Sampwe, galerie forestière de la rivière Mufungwe, 10 Apr 1949, *G.F.de Witte 6005* (BR).

##### Notes.

1. New species record for DR. Congo.

2. The locality in Katanga is remarkably disjunct, ca. 1000 km in the SW of the nearest locations in N Tanzania.

3. Another collection from Upemba National Park (Katanga) (*G.F.de Witte 6449*), is intermediate between *C.succulentus* and *C.cylindraceus*.

#### ﻿ Coleus sylvestris

Taxon classificationPlantaeLamialesLamiaceae

(Gürke) A.J.Paton & Phillipson, Phytokeys 129: 103. 2019.

11B3B807-A414-5979-8CCC-188A8C05F425

≡ Plectranthussylvestris Gürke, Bot. Jahrb. Syst. 19: 205. 1894. Type: Tanzania, Kilimanjaro, Rifinika Hill on Mawenzi, 14 Sep 1893, *G.Volkens 965* (holotype B destroyed; isotype BM [BM000999974], G).
= Coleusferrugineus Robyns, Bull. Jard. Bot. État Bruxelles 17: 77. 1943. Type: DR. Congo, Karisimbi, Feb 1932, *J.Lebrun 5005* (holotype BR [BR0000006262617]; isotype K).
= Plectranthusferrugineus (Robyns) Troupin & Ayob., Fl. Rwanda 3: 336. 1985., nom. inval.
= Coleussubulatus Robyns, Bull. Jard. Bot. État Bruxelles 17: 76. 1943. Type: DR. Congo, Kivu, Tschamugussa. 13 Aug 1934, *G.F.de Witte 1854* (holotype BR [BR0000006262952]; isotype LWI).

##### Description.

Paton et al. (2009: 295), Paton et al. (2013: 243), as *Plectranthussylvestris* Gürke.

##### Distribution.

Tropical Africa, Madagascar.

##### Habitat and ecology.

Mountain evergreen forest, often with *Hagenia* and bamboo; 2300–3400 m elev.

##### Additional specimens.

DR. Congo, ***Lacs Edouard et Kivu***, Karisimbi, vallée Visoke, 22 Jan 1955, *G.F.de Witte 11568* (BR); Kabara, Mikeno, 16 Jul 1934, *G.F.de Witte 1777* (BR, LWI); Mont Muhi, Jun 1948, *F.L.Hendrickx 5273* (BR); Mont Bukulumiza, 26 Jul 1955, *R.Pierlot 666* (BR); ***Haut-Katanga***, Marungu, Ndawa, Apr 1944, *L.Dubois 1165* (BR). Rwanda, Forêt de Nyungwe, vers km 100, 21 Aug 1969, *G.Bouxin & M.Radoux 716* (BR); Kareba, 10 Oct 1974, *P.Auquier 4514* (BR); Karisimbi, versant Sud, 27 Feb 1935, *G.F.de Witte 2255* (BR); Karisoke, 3 Jan 2006, *Luksenberg & A.Nsanzurwimo s.n*. (BR).

Burundi, Bukeye, Teza, 19 Jun 1971, *J.Lewalle 6024* (BR); Muramvya, bois sacré de Mpotsa, 9 Jun 1979, *M.Reekmans 8245* (BR, WAG).

#### ﻿ Coleus tenuicaulis

Taxon classificationPlantaeLamialesLamiaceae

Hook.f., J. Proc. Linn. Soc., Bot. 7: 211. 1864.

8DF4757E-6850-5FB2-A68D-4FFBEB002699

##### Type.

Cameroon, Mt Cameroon, Dec 1862, *G.Mann 1939* (holotype K [K000431854]).

##### Description.

Perennial herb 0.3–0.65(–1.50) m high, rootstock weakly rhizomatous. Stem erect, quadrangular, variously pubescent, often with short retrorse hairs, occasionally with patent eglandular hairs, short glandular hairs and papillae, densely glandular pubescent in the inflorescence, erect, branched. Leaves opposite, patent, often with young leaves in the axils, petiolate, upper ones subsessile; blade ovate-triangular, (narrowly ovate out of Central Africa), 1.5–6.5(–7.5) × 1.0–5.0(–6.5) cm, base broadly rounded, truncate or subcordate, more rarely cuneate, shortly attenuate in the petiole, apex acute, margin serrate, upper surface densely pubescent (very short papilliform hairs), lower surface pubescent on veins, ca. 4–5 pairs of secondary veins; petiole 1.0–3.5(–4.0) cm long, with very short retrorse hairs and often also long patent hairs. Inflorescence terminal, lax, 4–18 cm long, with 5–14 verticils spaced 7–30 mm, bracts narrowly ovate, ca. 3 mm long, acuminate, caducous or rarely persistent, cymes ascending, ca. 9–25-flowered, pedunculate, peduncle 1–15 mm long, with two opposite cincinni ca. 5–50 mm long, densely papillate, pedicel 1–4 mm long, widely spaced, inserted eccentrically. Flower: calyx 2 mm long at anthesis, densely papillate and with red sessile glands, ca. 5–6 mm long in fruit, tube slightly curved, slightly constricted at throat, upper lip ovate, ca. 2–3 mm long, markedly curved, subacute, slightly decurrent, lobes of lower lip narrowly triangular, sharply acute, the lateral ones 2 mm long, the middle ones 3 mm long. Corolla pale blue to violet, with pale sessile glands, ca. 10–15 mm long, tube strongly sigmoid, ca. 4 mm long, lower lip ca. 6–8 mm long, 3–4 mm deep, enclosing stamens; filaments fused, anther ca. 0.8 mm, style entire. Nucule pale brown, round, compressed, ca. 1 mm diam., dull, smooth.

##### Distribution.

W Tropical Africa to Cameroon, SW Tanzania to S Tropical Africa.

##### Habitat and ecology.

Dembo, wooded savannah, most often on moist soil; 800–1815 m elev.

##### Additional specimens.

DR. Congo, ***Kasaï***, Kwango, Twana, 12 Sep 1953, *H.Callens 4223* (BR); Kwango, Kibunda, 27 Apr 1953, *H.Callens 4001* (BR); 50 km W of Kimvula, 12 Apr 1948, *P.Duvigneaud 715* (BRLU); ***Haut-Katanga***, Upemba, riv. Lusinga, 21 Oct 1948, *L.van Meel* in *de Witte 4566* (BR); Upemba, 29 Jul 1949, *L.van Meel* in *G.F.de Witte 7128* (BR); Kasombo, 22 Jan 1957, *E.Detilleux 457* (BR); Kundelungu, 6 km NNW poste de Katshupa, rivière Luanza, 29 Jul 1966, *F.Malaisse 4213b* (BR, LSHI); Kundelungu, rivière Kabunda, 28 Mar 1971, *S.Lisowski 23387* (POZG); Dembo de la Katuba, Feb 1934, *P.Quarré 3833* (BR).

##### Notes.

1. New species record for DR. Congo.

2. *P.Quarré 3833* (BR) and *Lisowski, Malaisse & Symoens 4930* (POZG) are unusual in having lower leaf surface and stem tomentose.

#### ﻿ Coleus tetradenifolius

Taxon classificationPlantaeLamialesLamiaceae

(A.J.Paton) A.J.Paton, Phytokeys 129: 104. 2019.

212664E9-8340-5E69-9205-F7DFDC3BA8FB

≡ Plectranthustetradenifolius A.J.Paton, Fl. Trop. E. Afr., Lamiac.: 304. 2009. Type: Uganda, Karamoja District, Mt Moroto, Jun 1963, *E.Tweedie 2665* (holotype K [K000430736]).

##### Description.

Paton et al. (2009: 304), as *Plectranthustetradenifolius* A.J.Paton.

##### Distribution.

Cameroon, S. Sudan to E. Tropical Africa and NE DR. Congo.

##### Habitat and ecology.

Savannah with *Exothecaabyssinica* (out of Central Africa: rocky slopes and cliffs); ca. 2450 m elev. (out of Central Africa: 1350–2700 m elev.).

##### Additional specimens.

DR. Congo, ***Lac Albert***, Mont Aboro, 2450 m elev., 26 Mar 1958, *P.Bamps 147* (BR).

##### Notes.

1. New species record for DR. Congo.

2. The specimen collected in DR. Congo departs from the type in having the inflorescence axis without glandular hairs and a shorter corolla (ca. 6 mm vs. 8–10 mm).

#### ﻿ Coleus thyrsoideus

Taxon classificationPlantaeLamialesLamiaceae

Baker, Bot. Mag. 125: t. 7672 1899.

B91BD579-1FEA-53A3-9F95-13B9D5D9DD91

≡ Plectranthusthyrsoideus (Baker) B.Mathew, Kew Bull. 31: 174. 1976. Type: Plant cultivated at Kew, seed from herbarium specimen from N of Lake Malawi collected by Whyte, 3 Jan1899 (holotype K [K000430792]).

##### Description.

Paton et al. (2013: 258), as *Plectranthusthyrsoideus* (Baker) B.Mathew.

##### Distribution.

S Tropical Africa.

##### Habitat and ecology.

Savannah and shrub savannah on rocky slopes; 1200–1300 m elev.

##### Additional specimens.

DR. Congo, ***Haut-Katanga***, Territ. Sakania, SE de Kipushia, Mont Lukanga, 29 Apr 1971, *S.Lisowski 23388* (POZG); Env. Kasumbalesa, colline Kibwe I, 20 Mar 1971, *S.Lisowski 23315* (POZG).

##### Note.

New species record for DR. Congo.

#### ﻿ Coleus welwitschii

Taxon classificationPlantaeLamialesLamiaceae

Briq., Bot. Jahrb. Syst. 19: 185. 1895.

78748614-8B30-5397-B7C8-61C438456D7B

= Coleusdupuisii Briq., Bull. Soc. Roy. Bot. Belgique 37: 70. 1899.
≡ Plectranthusdupuisii (Briq.) A.J.Paton, Fl. Trop. E. Afr., Lamiac.: 329. 2009. Type: DR. Congo, Kasai, Mayumbe, Jul 1893, *Dupuis s.n.* (holotype BR [BR0000008109132]).
= Solenostemonthyrsiflorum (Lebrun & L.Touss.) Vollesen, Opera Bot. 59: 85. 1980., nom. superfl. Type: Rwanda, Kagera, Nyakayaga, Jan 1938, *J.Lebrun 9447* (holotype BR [BR0000006263003]; isotype K, P).
= ?Coleuslaurentii De Wild. (1920) 121. Bol. Soc. Ibér. Ci. Nat. 19: 121. 1920. Type: DR. Congo, Gombe, 14 Dec 1903, *E.&M.Laurent s.n.* (holotype BR [BR0000006258580] & [BR0000008109798]).

##### Type.

Angola, Pungo Andongo, *F.Welwitsch 5589* (syntypes BM [BM000564040], [BM000999976], C [C10001537], G, K, LISU [LISU220997], [LISU220998], MEL, P), & *A.von Mechow 75* (syntype, not seen).

##### Description.

Paton et al. (2009: 329), Paton et al. (2013: 268), as *Plectranthusdupuisii* (Briq.) A.J.Paton.

##### Distribution.

Ethiopia to S. Tropical Africa.

##### Habitat and ecology.

Savannah, steppic savannah, often on shallow rocky soil, xerophilous scrub, more rarely forest and woodland (termite mounds); 100–2200 m elev.

##### Additional specimens.

DR. Congo, ***Côtier***, Banana, Mar 1948, *H.Callens 1066* (BR); ***Bas-Congo***, Léopoldvile [Kinshasa], Matete, 5 Aug 1956, *A.Carlier 334* (BR); ***Ubangi-Uele***, Garamba, 14 May 1950, *H.De Saeger 511* (BR); Garamba, route Dungu-Bagbele, km 17 de Bagbele, 27 Aug 1952, *G.Troupin 2028* (BR); ***Lacs Edouard et Kivu***, Plaine de la Ruzizi, sommet Tsamate, Apr 1950, *R.Germain 6826* (BR).

Rwanda, Bugesera, 27 Mar 1970, *G.Bouxin & M.Radoux 1631* (BR); Rusumo, route vers Nyarubuye, 25 Jan 1980, *D.Bridson 299* (BR, WAG); Kibungo, Rusumo, 16 Oct 1974, *J.Lambinon 74/1574* (BR, LG); Route Nyamasheke-Kibuye, 29 Mar 1972, *G.Troupin 6510* (BR, WAG); Kitega, Bweru, Muhweza, 27 Nov 1957, *van der Ben 1727* (BR).

Burundi, Buterama, Gasorwe, 22 May 1959, *van der Ben 2558* (BR); Kameramagambo, 27 Dec 1965, *J.Lewalle 155* (BR, MO); Gitwenge, 2 Jan 1979, *M.Reekmans 7448* (BR); Kagoma, E de Gitwenge, 7 Feb 1979, *M.Reekmans 7639* (BR, WAG); Muramvya, Komwe, 24 May 1981, *M.Reekmans 10355* (BR, MO, WAG).

##### Notes.

1. *Coleuslaurentii* De Wild. is considered as a synonym of *C.welwitschii* by Paton et al. (2019). However, the type material (*E.& M.Laurent s.n.* [BR0000008109798], [BR0000006258580]) is remarkable in being almost glabrous in all vegetative parts; collecting notes on the label indicate the presence of white tubers (not collected); it could represent a different taxon (*C.rotundifolius*?).

2. *L.Liben 2855* is somewhat intermediate between *C.bojeri* and *C.welwitschii*, having robust lignified shoots, but lacking a true rhizome.

3. Many specimens from Rwanda, Burundi and W Tanzania have greyish-tomentose lower leaf surface and thicker leaves, corresponding to the type specimen of *Coleusthyrsiflorus*, but there is no real discontinuity with less pubescent forms.

4. See also note under *C.brazzavillensis*.

#### ﻿ Coleus zigzag

Taxon classificationPlantaeLamialesLamiaceae

Meerts & A.J.Paton sp. nov.

8BB89183-02EC-5B36-AC2A-FD4D42AB052B

urn:lsid:ipni.org:names:77347707-1

Fig. 18A–H

##### Type.

DR. Congo, Ubangi-Uele, Parc national de la Garamba, Ndelele, colline rocheuse, sol humifère dans les dépressions, 26 Aug 1951, *H.De Saeger 1413* (holotype BR [BR0000017707787]; isotype K).

##### Diagnosis.

Related to *Coleusbojeri* and other species formerly referred to the genus *Solenostemon* on account of lower calyx lobes fused into a lip, differing by the pedunculate cyme, subglabrous rachis, divaricate zigzagging cincinni, broadly cordiform to reniform foliar blade.

##### Description.

Annual or perennial herb, 0.5–1.0 m high, more or less tufted, not reported to be aromatic; tubers lacking or not collected. Stem erect or ascending, quadrangular, more or less lignified in lower part, sparingly branched, with a mixed indumentum of very short, papilliform hairs and sparse, long, patent, multicellular hairs, these sometimes almost lacking, also with sparse sessile red glands, becoming subglabrous to papillate in the inflorescence. Leaves opposite, spreading, petiolate, blade broadly ovate to cordiform or almost reniform, shorter than the petiole, (1.0–)2.0–4.5 × (0.8–)1.8–4.0 cm, apex rounded to subacute, base truncate (in the smallest leaves) to cordate, then shortly attenuate into the petiole, margin often recurved, purplish, strongly crenate to serrate, teeth rounded, ca. 4/cm, 4–5 secondary veins on either side, densely covered with very short papilliform hairs on both surfaces, also with sparse long hairs, lower surface also with many red sessile glands, reticulum prominent below; petiole 1.5–6 cm long, canaliculate, pubescent like the stem. Inflorescence unbranched or branching at lower nodes, 15–40 cm long, lax, nodes (5–)15–60 mm apart, verticils (15–)30–50-flowered, cymes all pedunculate, dichasial, peduncle 5–15 mm, with two divaricate cincinni up to 30 mm long in fruit, zigzagging, with 5–22 flowers, papillate, often purplish; pedicel 0.5–1 mm long in flower, ca. 2 mm in fruit, attached eccentrically behind calyx posterior lip. Flower: calyx ca. 1 mm long at anthesis, pubescent, with red sessile glands, fruiting calyx 2.5–3(–4) mm long, shortly tubular or campanulate, papillate and with red sessile glands, throat truncate, posterior lip obovate, ca. 1.5 mm long, obtuse to rounded, apiculate, slightly recurved, not decurrent, lateral lobes rectangular-oblong, truncate to rounded, median lobes of anterior lip linear, fused into a linear lower lip, straight, projecting well beyond the other lobes, with two fine points curving upwards 0.5–1 mm long. Corolla with red sessile glands, (3–)8–10 mm long, tube sigmoid, ca. 2 mm long, upper lobe 2 mm long, shortly pubescent, lower lip blue to purple, (2–)3–6 mm long, cucullate, 2–3 mm deep, enclosing stamens, shortly pubescent and with red sessile glands, stamens fused in lower half. Nutlets shiny brown, slightly compressed, 0.8–1 mm.

**Figure 18.**
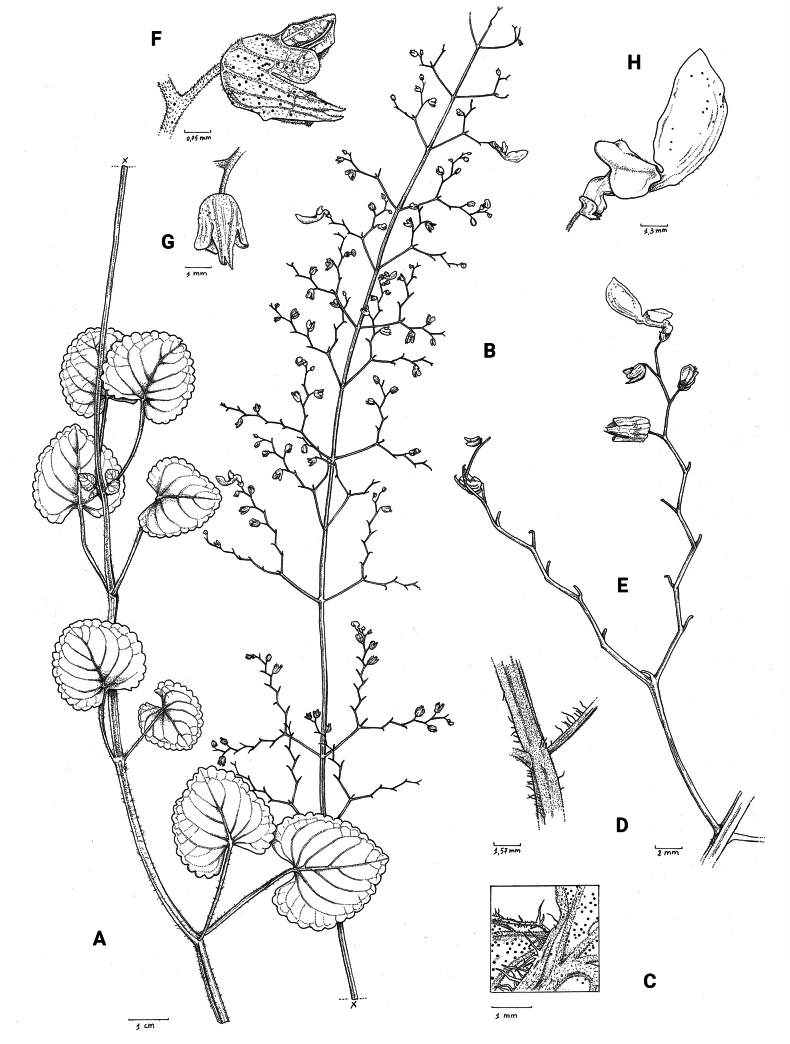
*Coleuszigzag* Meerts & A.J.Paton **A** stem and leaves **B** inflorescence **C** detail of petiole apex **D** detail of pubescence of inflorescence **E** cyme **F** fruiting calyx, side view **G** calyx seen from below **H** flower (**A***H.De Saeger 1413 & 3849***B–E***H.De Saeger 1413***F, G***H.De Saeger 3849*). Drawn by Hilde Orye.Scale bars: 1 cm (**A, B**); 1 mm (**C**); 1.57 mm (**D**); 2 mm (**E**); 0.75 mm (**F**); 1 mm (**G**); 1.3 mm (**H**).

##### Etymology.

The epithet refers to the characteristic zigzagging rachis of the cymes.

##### Distribution.

W Uganda and NE DR. Congo.

##### Habitat and ecology.

Rock crevices, savannah on shallow rocky soil; ca. 700–850 m elev.

##### Additional specimens.

DR. Congo, ***Ubangi-Uele***, Parc national de la Garamba, Ndelele, fissures et dépressions rocheuses, 27 Sep 1952, *H.De Saeger 3849* (BR); Mont Genze, sur la roche, 30 Sep 1953, *P.Gérard 847* (BR); Entre Faradje et Dungu, savane rocheuse au bord du Kibali, crevasses dans les roches, Aug 1931, *J.Lebrun 3458* (BR, K); Parc National de la Garamba, frontière du Soudan, près de Ndelele, affleurement rocheux, pelouse rase à *Cyanotis* et *Sporobolus*, 13 Aug 1952, *G.Troupin 1848* (BR); ***Forestier Central***, Haut-Zaïre, Ituri, env. de Nduye, Mont Mukonza, rochers, 8 Aug 1975, *S.Lisowski 40451* (POZG); Same locality, 5 Jan 1976, *S.Lisowski 41509, 41738* (POZG); Haut-Zaïre, Ituri, env. de Nduye, au-dessus du village Maitatu, Mont Mukonza, 12 Apr 1976, *S.Lisowski 42315, 42498* (POZG).

Uganda, West Nile Distr., Rokosa Hill, ¾ mile SE of Maracha [illegible] Camp, 6 Aug 1953, *R.J. Chancellor 113* (K).

##### Note.

The specimens from Ituri (region of Nduye), ca. 250 km south of the range of the species in the Garamba Region, match the type in all traits except for the almost straight, not zigzagging cincinni; we consider them as conspecific.

### ﻿﻿Unplaced names

*Coleuspoggeanus* Briq., Bot. Jahrb. Syst. 19(2–3): 182. 1894. Type: DR. Congo, Mussumba des Muata Jamwo [Mosumba Kekese], S08°1/2, Jan 1876, *P.Pogge 364* (holotype B destroyed).

*Coleusviridis* Briq., Bot. Jahrb. Syst. 19(2–3): 181. Type: DR. Congo, Mussumba des Muata Jamwo [Mosumba Kekese], S08°1/2, Jan 1876, *P.Pogge 365* (holotype B destroyed).

### ﻿﻿Species excluded

*Coleusschizophyllus* (Baker) A.J.Paton was reported from DR. Congo in error, based on *M.Schaijes 1889* (BR) and *F.Malaisse & E.Robbrecht 2356* (BR), which are *Equilabiumpulcherrimum* (A.J.Paton) Mwany. & A.J.Paton.

*Coleustetragonus* (Gürke) Robyns & Lebrun, was reported from DR. Congo in error, based on *A.Hock s.n*. [BR0000009824928], the type specimen of *Plectranthusdekindtianus* Gürke. However, this specimen clearly belongs in C.esculentusvar.densus (see note under that variety). The specimen *W.Robyns 2061* (BR) was also misidentified as *C.tetragonus*, while it is actually *C.conglomeratus*. A specimen of *C.tetragonus* was collected in Zambia very close to the DR. Congo border (*Brédo 3123* [BR]). However, no authentic materials of *C.tetragonus* have been seen from DR. Congo, Rwanda and Burundi so far.

## Supplementary Material

XML Treatment for Coleus affinis

XML Treatment for Coleus alpinus

XML Treatment for Coleus amboinicus

XML Treatment for Coleus articulatus

XML Treatment for Coleus autranii

XML Treatment for Coleus barbatus

XML Treatment for Coleus barbatus var. barbatus

XML Treatment for Coleus barbatus var. grandis

XML Treatment for Coleus batesii

XML Treatment for Coleus betonicifolius

XML Treatment for Coleus betonicifolius (Baker) A.J.Paton var. betonicifolius

XML Treatment for Coleus betonicifolius (Baker) A.J.Paton var. kasomenensis

XML Treatment for Coleus bojeri

XML Treatment for Coleus brazzavillensis

XML Treatment for Coleus buchananii

XML Treatment for Coleus calaminthoides

XML Treatment for Coleus caninus subsp. flavovirens

XML Treatment for Coleus celsus

XML Treatment for Coleus chevalieri

XML Treatment for Coleus collinus

XML Treatment for Coleus conglomeratus

XML Treatment for Coleus cylindraceus

XML Treatment for Coleus decimus

XML Treatment for Coleus decurrens

XML Treatment for Coleus deflexifolius

XML Treatment for Coleus defoliatus

XML Treatment for Coleus descampsii

XML Treatment for Coleus dewildemanianus

XML Treatment for Coleus duvigneaudii

XML Treatment for Coleus efoliatus

XML Treatment for Coleus elliotii

XML Treatment for Coleus eminii

XML Treatment for Coleus engleri

XML Treatment for Coleus erici-rosenii

XML Treatment for Coleus esculentus

XML Treatment for Coleus esculentus var. esculentus

XML Treatment for Coleus esculentus var. densus

XML Treatment for Coleus esculentus var. primulinus

XML Treatment for Coleus esculentus var. kolweziensis

XML Treatment for Coleus foliatus

XML Treatment for Coleus frederici

XML Treatment for Coleus globosus

XML Treatment for Coleus goetzenii

XML Treatment for Coleus gracilipedicellatus

XML Treatment for Coleus gracillimus

XML Treatment for Coleus guerkei

XML Treatment for Coleus hadiensis

XML Treatment for Coleus heterotrichus

XML Treatment for Coleus hildei

XML Treatment for Coleus homblei

XML Treatment for Coleus kaminaensis

XML Treatment for Coleus kapatensis

XML Treatment for Coleus kivuensis

XML Treatment for Coleus kundelunguensis

XML Treatment for Coleus lactiflorus

XML Treatment for Coleus lanuginosus

XML Treatment for Coleus linarioides

XML Treatment for Coleus lisowskii

XML Treatment for Coleus longipetiolatus

XML Treatment for Coleus maculosus

XML Treatment for Coleus maculosus (Lam.) A.J.Paton, subsp. maculosus

XML Treatment for Coleus maculosus subsp. edulis

XML Treatment for Coleus mannii

XML Treatment for Coleus marunguensis

XML Treatment for Coleus melleri

XML Treatment for Coleus meyeri

XML Treatment for Coleus mirabilis

XML Treatment for Coleus minusculus

XML Treatment for Coleus mitwabaensis

XML Treatment for Coleus modestus

XML Treatment for Coleus monostachyus

XML Treatment for Coleus monostachyus (P.Beauv.) A.J.Paton subsp. monostachyus

XML Treatment for Coleus mystax

XML Treatment for Coleus parvifolius

XML Treatment for Coleus pengbelensis

XML Treatment for Coleus penicillatus

XML Treatment for Coleus piscatorum

XML Treatment for Coleus prittwitzii

XML Treatment for Coleus pseudoschizophyllus

XML Treatment for Coleus pseudospeciosus

XML Treatment for Coleus repens

XML Treatment for Coleus rhodesianus

XML Treatment for Coleus rotundifolius

XML Treatment for Coleus ruandensis

XML Treatment for Coleus ruziziensis

XML Treatment for Coleus schliebenii

XML Treatment for Coleus scruposus

XML Treatment for Coleus seretii

XML Treatment for Coleus shirensis

XML Treatment for Coleus sphaerocephalus

XML Treatment for Coleus stachyoides

XML Treatment for Coleus stenostachys

XML Treatment for Coleus stuhlmannii

XML Treatment for Coleus succulentus

XML Treatment for Coleus sylvestris

XML Treatment for Coleus tenuicaulis

XML Treatment for Coleus tetradenifolius

XML Treatment for Coleus thyrsoideus

XML Treatment for Coleus welwitschii

XML Treatment for Coleus zigzag

## ﻿﻿Additional information

### Author ORCIDs

Pierre J. Meerts https://orcid.org/0000-0003-4215-027X

Alan J. Paton https://orcid.org/0000-0002-6052-6675

### Data availability

All of the data that support the findings of this study are available in the main text or Supplementary Information.

## Supplementary materials

﻿Supplementary material 1 Herbarium specimens examined Data type xlsx https://binary.pensoft.net/file/1126726This dataset is made available under the Open Database License (http://opendatacommons.org/licenses/odbl/1.0/). The Open Database License (ODbL) is a license agreement intended to allow users to freely share, modify, and use this Dataset while maintaining this same freedom for others, provided that the original source and author(s) are credited.Pierre J. Meerts, Alan J. Paton
